# Terminal glucose as a receptor for adeno-associated virus 44.9

**DOI:** 10.1128/jvi.00254-25

**Published:** 2026-03-23

**Authors:** Giovanni Di Pasquale, Hemon Tehrani, Ida Shinder, Sandra Afione, John A. Chiorini

**Affiliations:** 1 Adeno-Associated Virus Biology Section, National Institute of Dental and Craniofacial Research, National Institutes of Health 2511, Bethesda, Maryland, USA; College of Agriculture & Life Sciences, University of Arizona, Tucson, Arizona, USA

**Keywords:** adeno-associated virus, gene therapy, glycan, salivary gland, eye, AAV44.9

## Abstract

The isolation of novel adeno-associated virus (AAV) serotypes has led to significant advances in our understanding of parvovirus biology and vector development for gene therapy by identifying isolates with unique cell tropism and increased efficiency of gene transfer to target cells. AAV44.9 is a natural isolate originally found as a contamination of the laboratory stock of SV15 adenovirus. Its sequence homology places it between current clades in a small cluster with AAVrh.8R. Recent studies have suggested that AAV44.9 is a promising candidate for photoreceptor-targeted tissues, salivary glands, and other tissues. To better understand its tropism, glycan arrays and competition experiments were used to define its binding requirements for interacting with glucose and glucosamine on N-linked cell surface carbohydrates. Subsequent studies confirmed the role of these glycans in AAV44.9 transduction. Mutagenesis experiments were also used to identify amino acids responsible for glycan recognition and specificity.

**IMPORTANCE:**

Adeno-associated virus (AAV) vector technology is rapidly advancing and becoming the leading vector platform not only in the field of gene therapy but also a useful tool for functional genomic studies of novel proteins. The characterization of the biologic activities of these vectors is critical to the further application of these vectors in gene therapy and understanding their natural tropism. In this study, we have identified terminal glucose as a unique glycan receptor for AAV 44.9 vectors. We have confirmed this binding activity via multiple methods and showed that the interaction is critical to vector transduction of AAV44.9. Furthermore, by comparing its sequence and difference in binding activity to another closely related but distinct vector AAVrh.8R, we have defined amino acids on the surface of the capsid that impact the interaction between AAV and terminal glucose.

## INTRODUCTION

A critical first step in virus infections is the attachment of the virus to a cell. This first step is initiated by contact between cell surface molecules that act as receptors for the virus and thus determine host range and govern susceptibility of specific cell types. The process of attachment is highly virus specific and may involve single or multiple receptors. Many viruses interact with cell surface glycans and adhere to the cell via low-affinity interactions, where they bind to an additional receptor with high affinity leading to viral entry (1). The identities of the low-affinity attachment factors are not known for many viruses.

Sialylated glycans and heparan sulfate proteoglycans often serve as viral attachment receptors to the cell surface due to their strong electrostatic charge. Heparan sulfate is reported as the primary receptor for Herpesviridae (2), as well as some adenoviruses (3). Similarly, interactions between glycans containing terminal sialic acid moieties and members of the Orthomyxoviridae, Reoviridae, and Polyomaviridae families are also reported (4). Interestingly, the *Parvoviridae* family is reported to bind a wide array of carbohydrates. Several adeno-associated virus (AAV) serotypes bind heparan sulfate proteoglycans, including AAV2 (5), AAV3B, AAV13, and AAV6 (1), whereas others utilize sialic acid for cell surface binding and entry, including AAV4 and AAV5 (6), AAV1/6 (7), and bovine AAV and AAV12 (8, 9). More recently, other AAV glycan interactions have been reported. AAV9 requires terminal galactose for binding and transduction (10, 11), and AAVrh.10 binds to lactosamine on glycan arrays (12). These results suggest that at least two members of the *Dependoparvovirus* genus of the *Parvoviridae* family may have a broader set of interacting molecules for cell attachment than previously recognized. Understanding these initial cell surface interactions is critical to defining the cell tropism and for the future development of improved vectors with engineered interactions.

Clustering the diverse number of reported AAV genomes based on sequence homology has begun to identify AAV subgroups. Members of one AAV subgroup that falls between the larger clades of AAV6 and AAV7 are composed of similar but independently identified isolates reported as AAVrh.8, AAVrh.8R, and AAV44.9 (13, 14). Functional characterization of these isolates has found robust transduction of the CNS, eye, and salivary gland (14, 15). While their phylogenetic clustering suggests novel interaction that could underlie their distinctive pattern of transduction, little is reported on the virus cell surface receptor attachment of this group of AAVs.

In this study, we employed glycan microarray analyses, biochemical, and enzymatic assays to characterize a novel dependovirus glycan interaction between AAV44.9 and terminal glucose or glucosamine containing glycans. Mutagenesis studies with the phylogenetically related AAVrh.8R confirmed this binding specificity and identified critical amino acid contact residues responsible for these interacting domains on the virus capsid.

## RESULTS

### AAV44.9 transduction is protein and glycan dependent but sialic acid and heparin independent

**Fig 1:**
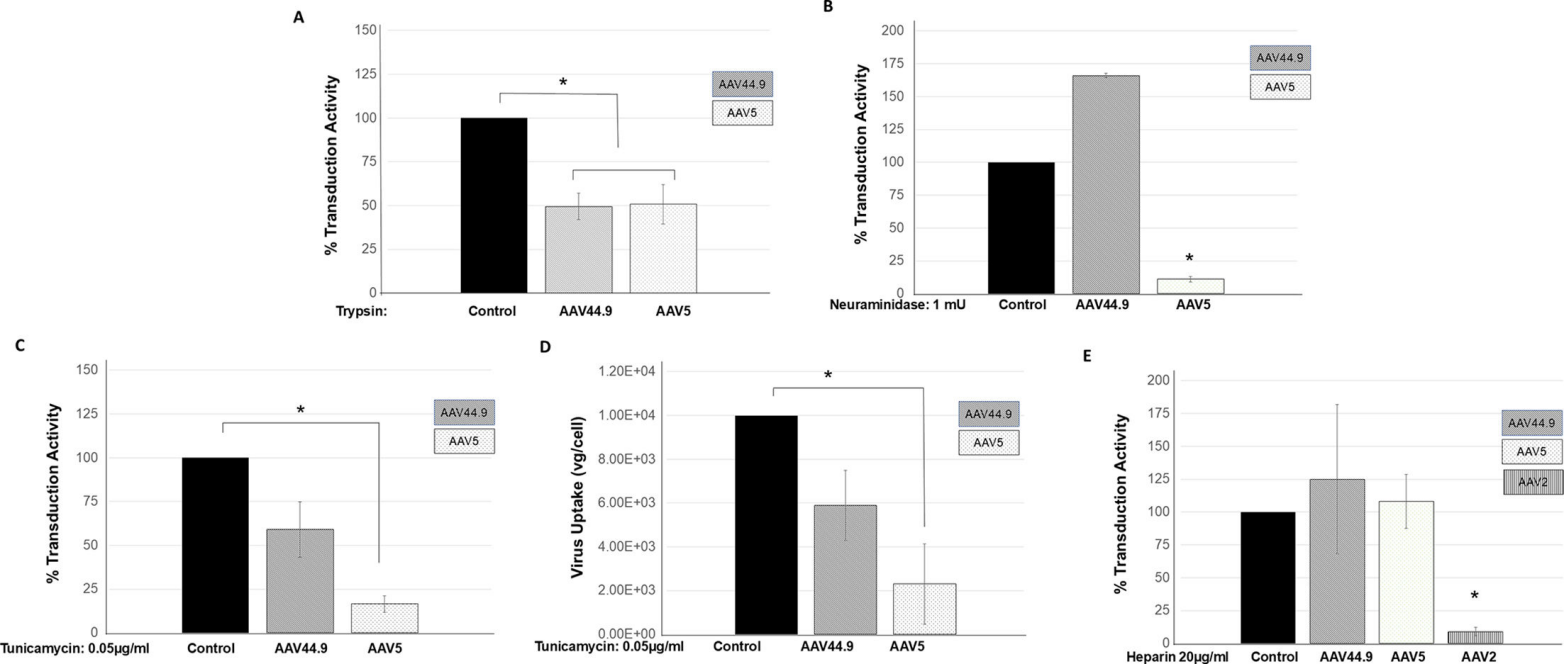
AAV44.9 transduction is dependent on cell surface proteins and glycans. (**A**) Cos-1 cells were digested proteolytically with trypsin before incubation with AAV44.9 or AAV5 CMV-*Gaussia*. Transduction, quantified by *Gaussia* luciferase activity, of AAV44.9 or AAV5 was compared to that of untreated cells, and it was used as control and was arbitrarily assigned as 100%. Results are the mean of four independent experiments. Error bars represent standard deviations (SDs). (**B**) Effect of neuraminidase (SA) on AAV44.9 transduction. Cos cells were pretreated with neuraminidase for 2 h before incubation with AAV44.9 or AAV5. Transduction was assayed, quantified, and compared as above. Results are the mean of three independent experiments. (**C**) Effect of N-linked inhibitors on AAV44.9 transduction. Cos cells were pretreated with tunicamycin for 24 h before incubation with AAV44.9 or AAV5. Transduction was assayed, quantified, and compared as in panel A. Results are the mean of five independent experiments. (**D**) Effect of N-linked inhibitors on AAV44.9 cell internalization. Cos cells were pretreated with tunicamycin and incubated with AAV44.9 or AAV5. Quantification of cell-internalized recombinants was determined by DNA cell extraction followed by viral DNA quantitative PCR. The amount of virus uptake was expressed as vg/cell. Results are the mean of five independent experiments. (**E**) Effect of soluble heparin sulfate on AAV44.9 transduction. AAV44.9, AAV5, and AAV2 were incubated in PBS with or without 20 µg of heparin sulfate/mL for 1 h, respectively. Then, the mixture was added to Cos cells and incubated for 1 h. After washing, cells were incubated for 48 h, and transduction was quantified and compared as in panel A. *P < 0.05 was considered statistically significant.

Despite the impressive transduction activity of AAV44.9 in the eye and the salivary gland, there is currently little known about the vector-cell interactions that are required for AAV44.9 transduction. Phylogenetic analysis demonstrates that evolutionary AAVrh.8R is on the same branch of a tree as AAV44.9 and is between clades A (AAV6) and D (AAV7) (Fig. S1) differing by four amino acids (Fig. S2). Since most AAVs are reported to require cell surface protein interactions for transduction (16), we explored the potential role of protein binding of AAV44.9 transduction. Preincubation of Cos cells with trypsin prior to the addition of AAV44.9 reporter vector encoding *Gaussia* luciferase decreased transduction compared to untreated controls (Fig. 1A). The decrease in transduction profile was similar to that of AAV5 (Fig. 1A). Transduction of a number of AAVs, including BAAV, AAV1, AAV4, AAV5, and AAV6, is reported to be sensitive to competition with soluble sialic acid and requires cell surface sialic acid glycans for efficient transduction (17). Incubation of Cos-1 cells with neuraminidase from *V. cholerae*, compared with untreated control cells, led to an increase in transduction (Fig. 1B). In contrast, neuraminidase treatment significantly inhibited AAV5 transduction compared with control cells (Fig. 1B). Additionally, incubation of cells with the N-linked glycosylation inhibitor tunicamycin significantly inhibited AAV44.9 transduction compared with untreated cells, suggesting that glycans were involved in AAV44.9 transduction (Fig. 1C). To confirm this loss of transduction, which was associated with a change in cell surface interactions and not non-specific trafficking effects of tunicamycin, the attachment of an AAV44.9 vector was quantified on cells treated with and without tunicamycin and then chilled to 4°C to prevent internalization. Later, the cells were washed, and the bound virus was measured by qPCR (Fig. 1D). Similarly to the observed decrease in transduction, treatment with tunicamycin also inhibited cell surface binding of AAV44.9. Heparan sulfate proteoglycan is the carbohydrate receptor of AAV2, AAV3B, AAV6, and AAV13, and it is necessary for transduction. To explore the role of surface heparan sulfate proteoglycan as a potential receptor, competition experiments were performed with soluble heparin. The results showed that AAV44.9 transduction was not inhibited, whereas AAV2 transduction was inhibited by soluble heparan (Fig. 1E), suggesting that AAV44.9 transduction is also independent of heparan sulfate proteoglycans. Taken together, these experiments suggest that AAV44.9 requires cell surface proteins and glycans for transduction, but its glycan-binding requirements are distinct from AAV5 (sialic acid sensitive) or AAV2 (heparin sensitive) and likely involve a novel glycan-binding receptor.

### Glycan microarray suggests AAV44.9 uses terminal glucose or galactose glycans for virus-cell interactions

**Fig 2:**
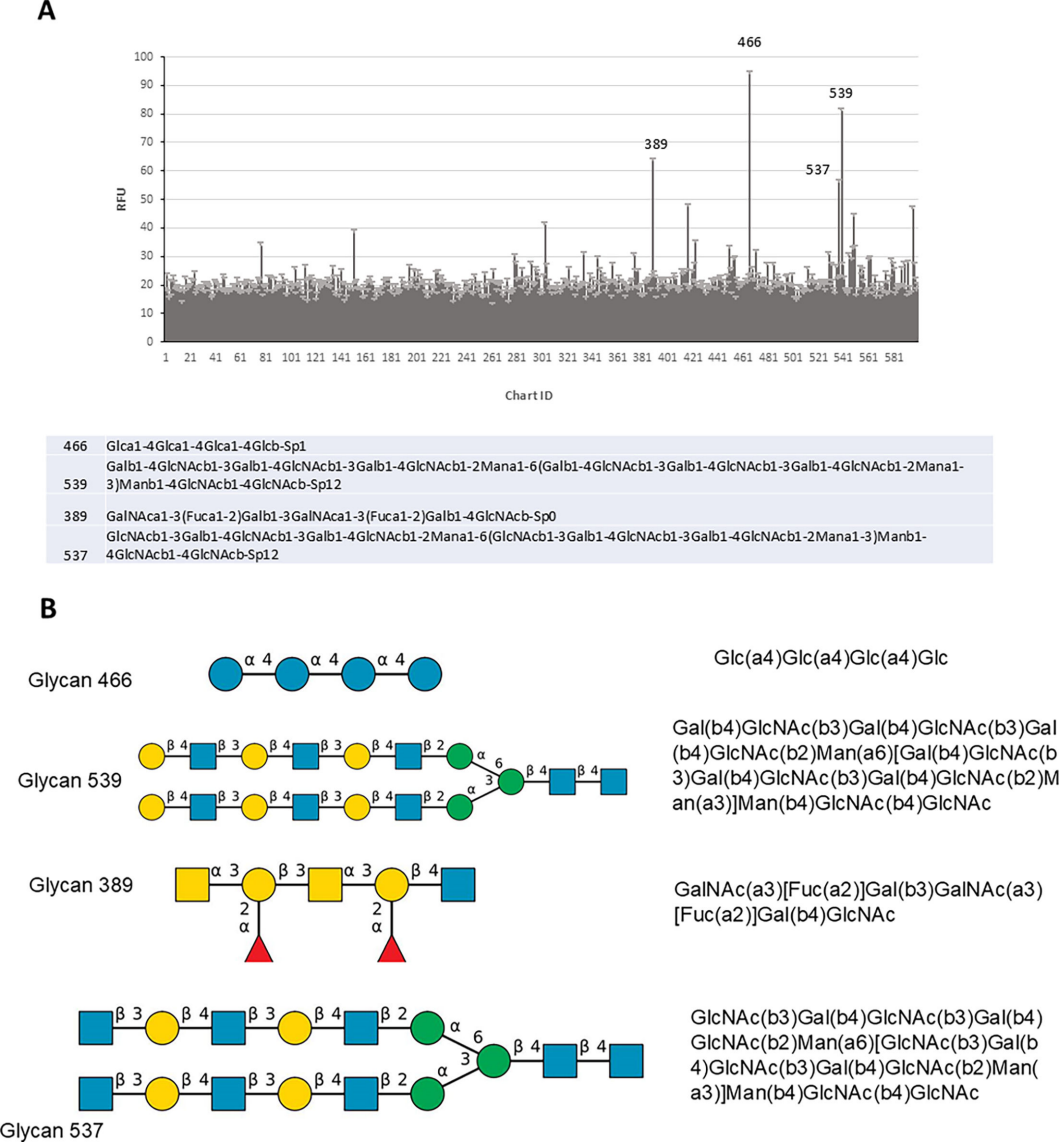
AAV44.9 binds glycans on a glycan microarray. (**A**) A glycan array slide containing ~600 glycans was screened to identify stable AAV44.9 carbohydrate interactions. The plot shows the average RFU with the SD measurement for each glycan versus glycan number. The values for the top four hit numbers and test nomenclature are listed in the figure. All four are terminal Gal and Glc N-linked glycans. (**B**) Structure, according to color code used in the Symbol Nomenclature for Glycans, of the four AAV44.9-binding candidates.

To better define the carbohydrate interactions that are necessary for AAV44.9 transduction, a glycan microarray composed of a panel of carbohydrate moieties commonly found on the mammalian cell surface was probed with the AAV44.9 vector. For these studies, the AAV44.9 vector was genetically tagged with an HA epitope after amino acid 137 of the VP1 and within the VP2 open reading frame. Bound virus was then detected using an FITC-conjugated anti-HA antibody, and virus-glycan binding was quantified as relative fluorescence unit (RFU). The most significant binding for AAV44.9 was detected with four glycans. Two of these glycans corresponding to #466 and #537 contain a terminal glucose (Glc), whereas the other glycans #539 and #589 contain a terminal galactose (Gal) (Fig. 2A and B). The results of this glycan screening also confirm that AAV44.9 transduction is heparan or sialic acid independent and suggests terminal galactose and/or glucose as the relevant glycan.

### Mutant CHO cells with terminal N-acetylglucosamine are more permissive for AAV44.9 transduction

**Fig 3:**
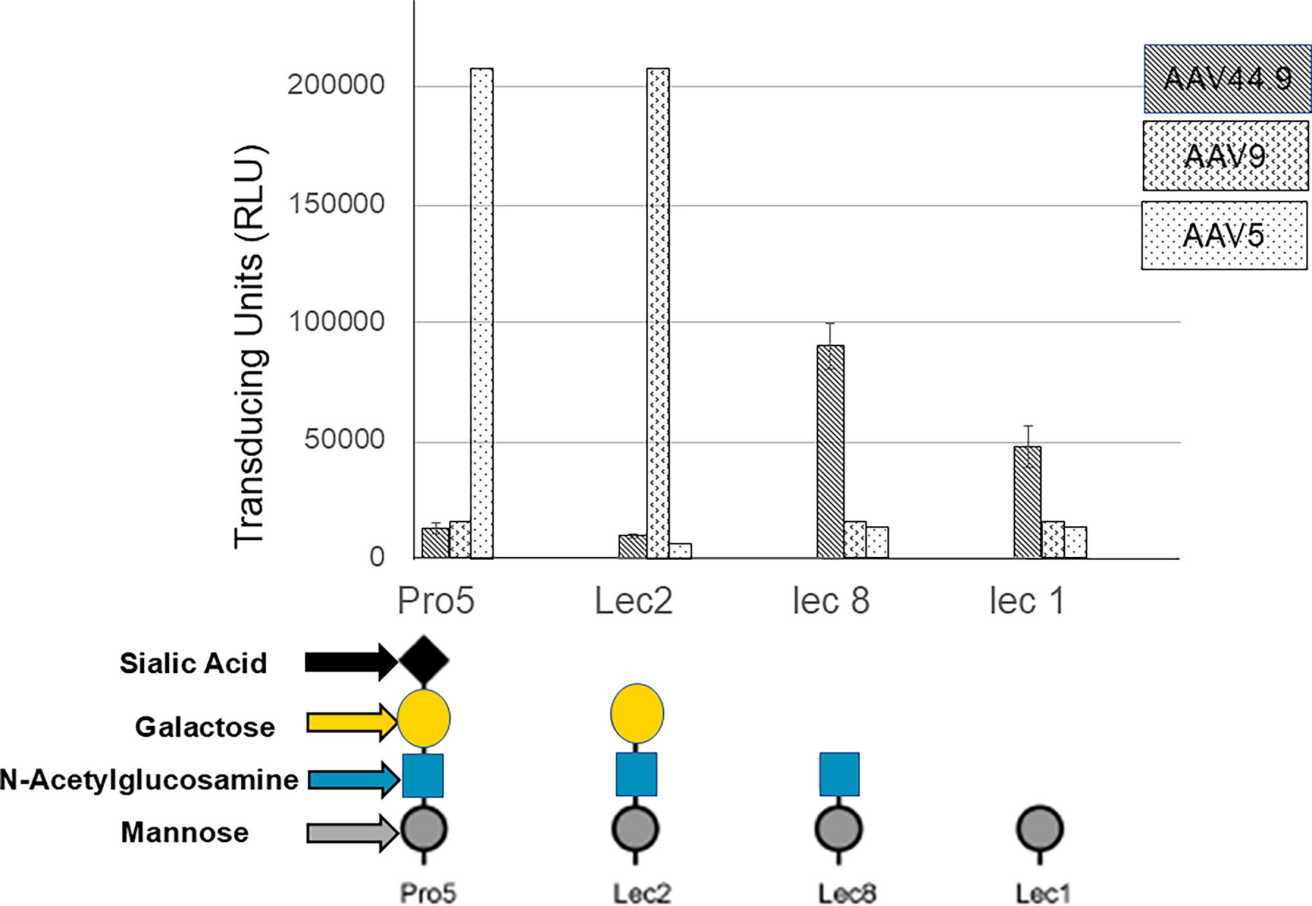
AAV44.9 transduction shows peak transduction on cells with terminal glucosamine. CHO Pro5 cells and three mutant cell lines were tested for transduction with AAV44.9 encoding *Gaussia* luciferase. AAV5 or AAV9 *Gaussia* luciferase was used as controls. The vector was incubated with the four different CHO cell lines for 2 h at 4^o^ C. After washing, *Gaussia* luciferase was quantified at 48 h post incubation. Transduction is presented as transducing units. Results are the mean of three independent experiments performed in quadruple.

To explore further the role of core glycans in AAV44.9 transduction, the CHO Pro5 cell line and three additional mutant cell lines derived from the parental Pro5 line that have enzymatic deficiencies in N-linked glycosylation (18) were used. These cell lines also were used previously to demonstrate AAV5 or AAV9 binding to the terminal sialic acid or galactose as glycan cell surface receptor, respectively (10, 11, 19). For our experiments, the AAV44.9 encoding *Gaussia* luciferase was incubated with Pro5 (terminal sialic acid), Lec2 (terminal galactose), Lec8 (terminal N-acetylglucosamine), and Lec1 (terminal mannose) cell lines, and transduction activity was measured (Fig. 3). AAV5 and AAV9, which are reported to require terminal sialic acid or galactose, respectively, were included as controls. As shown in Fig. 3, the AAV44.9 vector had limited transduction activity with the Pro5 and Lec2 cells compared with Lec8 cells, suggesting a role for terminal N-acetylglucosamine as an important molecule for interaction. Deficiency of the terminal N-acetylglucosamine in the Lec1 cells reduced transduction. In contrast, AAV5 efficiently transduced only the sialic acid-containing Pro5 cells, while the lack of sialic acid in Lec2 cells enhanced AAV9 transduction (Fig. 3). Taken together, these data support the role of N-acetylglucosamine in AAV44.9 transduction.

### Cell surface glucose-containing glycans impact AAV44.9 transduction 

**Fig 4:**
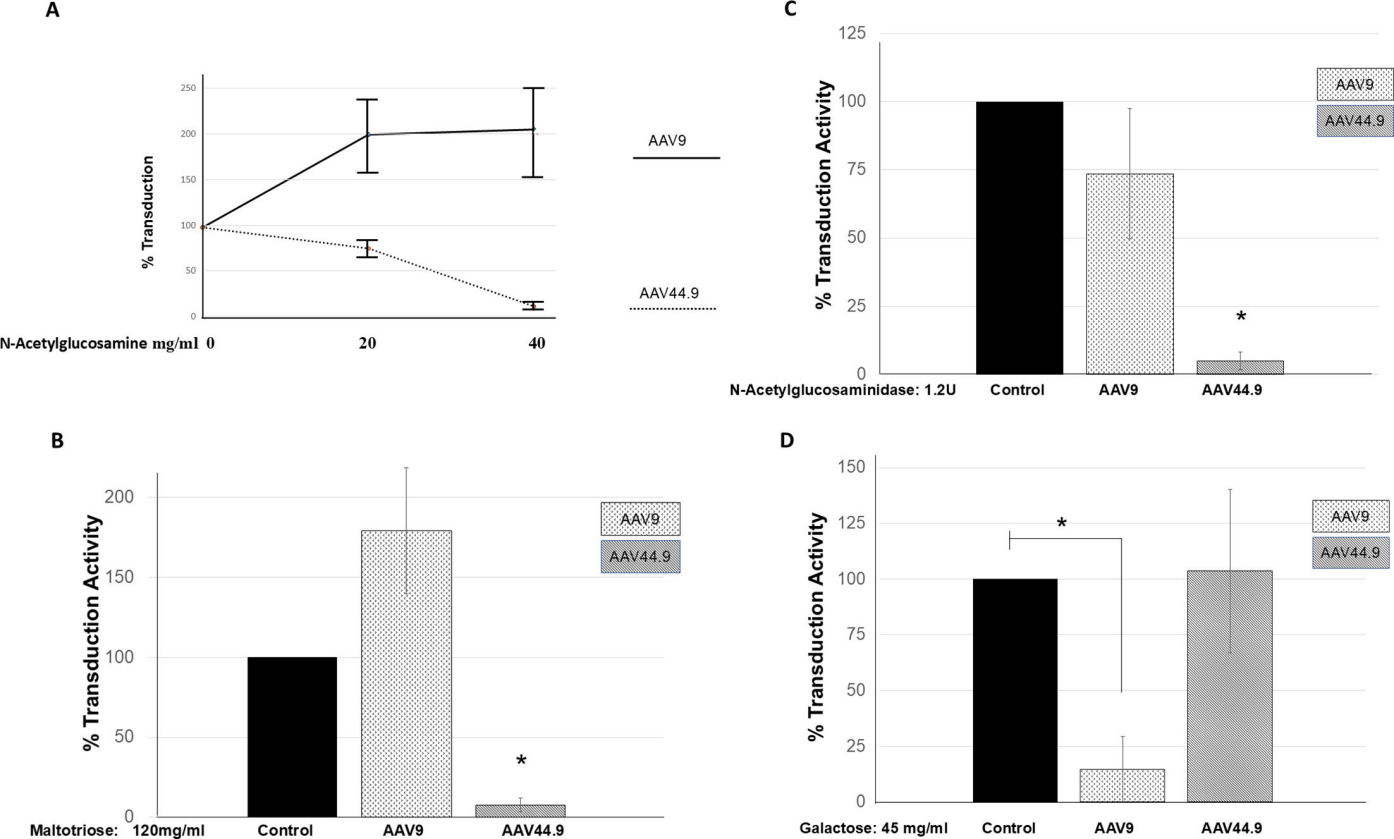
AAV44.9 transduction is sensitive to competition with soluble glycans. Following a 30-min preincubation with a serial dilution of glycan and a constant amount of vector, virus-glycan complexes are incubated for 2 h at 37°C with Cos cells and then removed, and *Gaussia* luciferase activity is quantified at 48 h post incubation. AAV9 is used as control. The data are normalized relative to control transduction without glycan. (**A**) Competition with N-acetylglucosamine. (**B**) Competition with maltotriose. (**C**) Competition with N-acetylglucosaminidase. (**D**) Competition with galactose. Results are the mean of three independent experiments performed in quadruple. ^*^P < 0.05 was considered statistically significant.

Next, we assessed if terminal N-acetylglucosamine was necessary for AAV44.9 transduction. For these experiments, the interactions between cell surface N-acetylglucosamine and AAV44.9 were either blocked by competition with soluble N-acetylglucosamine or removed from the cell surface enzymatically. Incubation of Cos-1 cells with soluble N-acetylglucosamine together with AAV44.9 inhibited transduction by more than 80% compared with control (Fig. 4A). In contrast, AAV9 transduction increased approximately two-fold (Fig. 4A). In a similar competition experiment with maltotriose, a trimer of glucose monosaccharides, 90% of the AAV44.9 transduction was inhibited compared to control cells treated with AAV44.9 alone, while AAV9 transduction increased. (Fig. 4B). Additional experiments by enzymatic removal of N-acetylglucosamine with specific glycosidase further supported a role for N-acetylglucosamine in AAV44.9 transduction. Here, treatment of Cos-1 cells with N-acetylglucosaminidase followed by incubation with AAV44.9 inhibited its transduction by more than 95% (Fig. 4C). In contrast, N-acetylglucosaminidase only had a minimal effect on AAV9 transduction (Fig. 4C).

The above data support the important role played by glucose or glucosamine in AAV44.9 transduction. AAV44.9 binding to galactose was tested by competition with soluble galactose and compared to the effect of galactose on AAV9, which requires galactose as a primary receptor. Incubating AAV44.9 with soluble galactose did not result in any change in AAV44.9 transduction compared with control cells transduced with AAV44.9 alone (Fig. 4D). On the other hand, the transduction of AAV9, a galactose-binding virus, was inhibited by more than 85% by incubation with soluble galactose (Fig. 4D). Altogether, these data suggest that either terminal glucose or N-acetylglucosamine is necessary for AAV44.9 transduction at the cell surface. These results also clearly demonstrate that AAV44.9 likely has a distinct receptor from other glycan-binding AAVs.

### Removal of cell surface N-acetylglucosamine reduces AAV44.9 cellular binding

**Fig 5:**
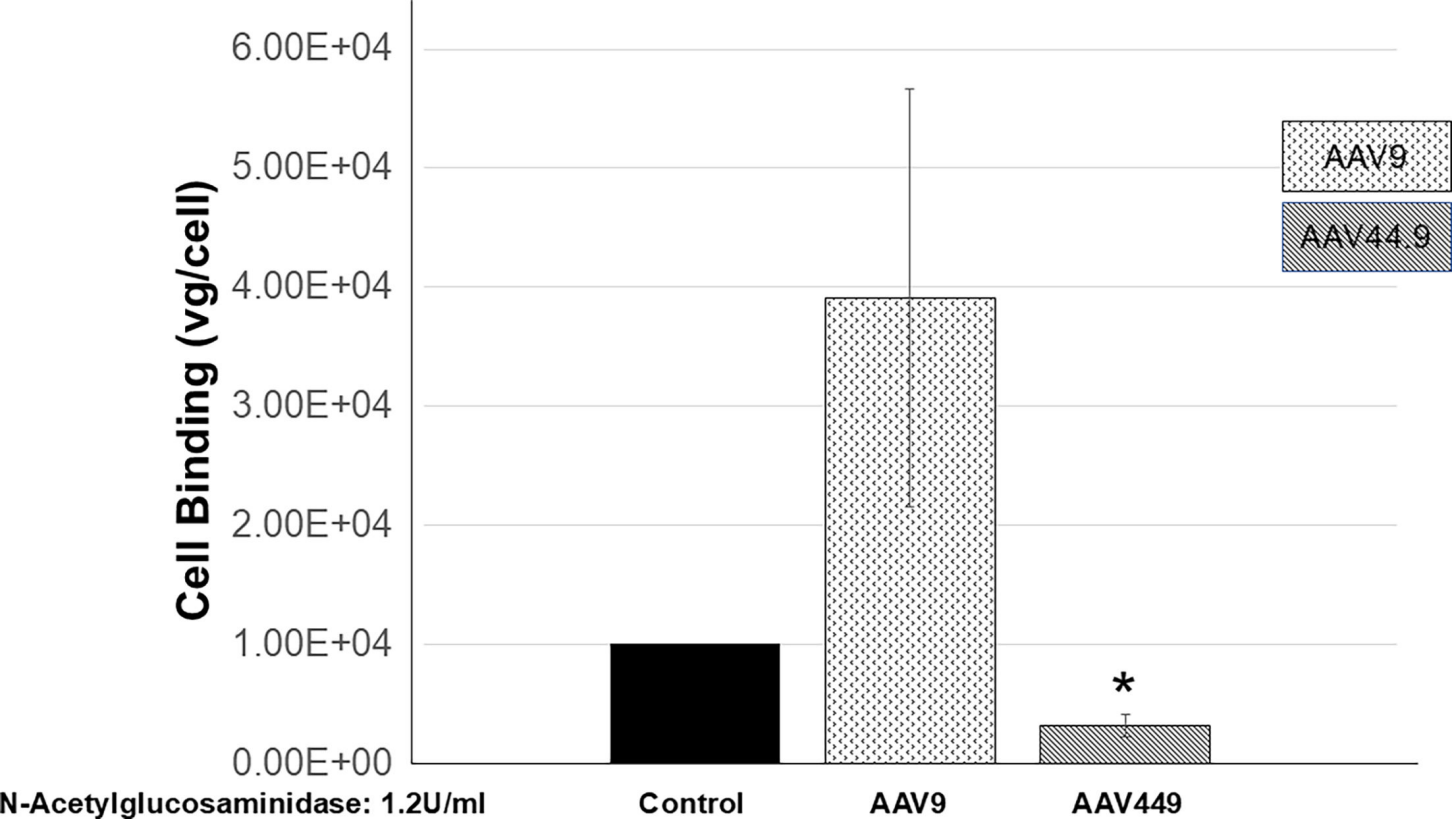
Treatment with N-acetylglucosaminidase inhibits AAV44.9 cell surface binding and internalization. Cell binding is expressed as vg/cell. Cos cells were treated with N-acetylglucosaminidase as above in Fig 4C. After washing, the cells were chilled at 4°C and incubated for 1 h with 44.9 vector. AAV9 is used as a control. After removing the unbounded particles by washing at 4°C, 48 h post incubation at 37°C, internalized vector DNA was quantified by qPCR from DNA cell extracts. DNA quantification is normalized relative to bounded particles without cell enzyme treatment. Results are the mean of three independent experiments in duplicate. *P < 0.05 was considered statistically significant.

AAV44.9 microarray glycan-binding assay suggested a physical interaction between the viral particle and N-acetylglucosamine residues. To further corroborate this interaction, cell surface-binding experiments were performed. Cos-1 cells chilled to 4°C were treated with N-acetylglucosaminidase to remove the glycan and then incubated with AAV44.9. After washing to remove the unbound virus, cell-associated vector was quantified by qPCR. As shown in Fig. 5, treatment of cells with N-acetylglucosamine decreased AAV44.9 binding to cells when compared with the untreated control Cos-1 cells. Compared with AAV44.9, cell binding of AAV9 was unaffected by the N-acetylglucosaminidase treatment, implying a distinct role for glucosamine in AAV44.9 transduction (Fig. 5). In summary, these experiments further support a direct physical interaction between AAV44.9 and glucose core containing glycans, and the effect is at the early stage of binding to the cell surface.

### Single amino acid changes on AAV44.9 lead to a loss of N-acetylglucosamine-mediated transduction

**Fig 6:**
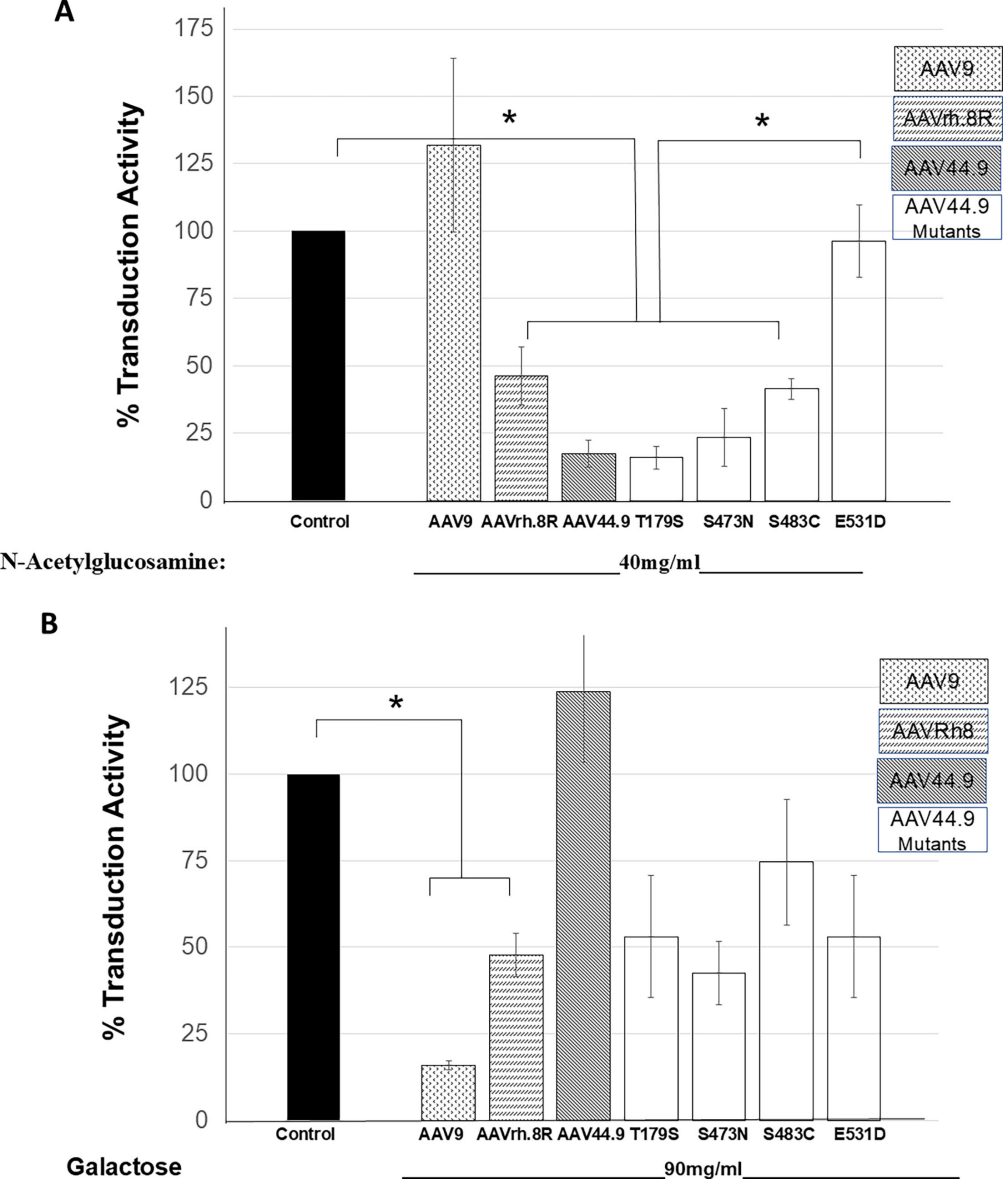
Amino acid substitutions alter glycan transduction sensitivity. Following a 30-min preincubation with a serial dilution of glycan and a constant amount of vector, virus-glycan complexes are incubated for 2 h at 37°C with Cos cells and then removed, and *Gaussia* luciferase activity is quantified 48 h post incubation. AAV9 is used as a control. The data are normalized relative to control transduction without glycan. (**A**) Incubation with N-acetylglucosamine. (**B**) Incubation with galactose. Results are the mean of three independent experiments performed in quadruple. *P < 0.05 was considered statistically significant.

AAV44.9 shares 99% homology with AAVrh.8R, differing in the capsid protein by four amino acids (Fig. S2). Although the amino acid 179 and 483 residues have not been reported to have an effect on AAV transduction, amino acids at or close to residues 473 and 531 are important in AAV9, AAV3, AAV6, and AAV13 transduction (17). Due to the location of these amino acids, we hypothesized that the substitution of AAVrh.8R amino acids at these positions into AAV44.9 could impact glycan binding or specificity. Thus, the following mutants were generated: AAV44.9-T179S, S473N, S483C, and E531D (Table 1). The transduction efficiency of AAV44.9, AAVrh.8R, and the mutants was compared in a competition assay on Cos-1 cells incubated in the presence or absence of N-acetylglucosamine or galactose, respectively (Fig. 6A and B). Despite the high-sequence homology between AAV44.9 and AAVrh.8R, soluble N-acetylglucosamine had relative effects on AAVrh.8R compared to AAV44.9, where transduction was significantly inhibited (Fig. 6A). Like AAV44.9, two of the mutants, T179S and S473N, were also inhibited by incubation with N-acetylglucosamine. On the other hand, the mutants S483C and E531D were not as strongly inhibited as AAV44.9. In fact, the E531D mutant lost inhibitor sensitivity to N-acetylglucosamine (Fig. 6A). Transduction competition assays with galactose further confirmed differences between AAV44.9 and AAVrh.8R. Lastly, incubation with galactose inhibited more than 50% of AAVrh.8R transduction activity compared to controls without treatment but had little effect on AAV44.9 transduction (Fig. 6B). Surprisingly, transduction of all four AAV44.9 mutants was affected by galactose, similar to that of AAVrh.8R (Fig. 6B). The transduction data established a difference in glycan usage and specificity between AAV44.9 and AAVrh.8R and identified critical amino acids involved in glycan binding in these evolutionarily related isolates.

**TABLE 1:** Mutants generated

Residue in AAV44.9	Amino acid position	Residue in AAVrh.8R
Thr (T)	179	Ser (S)
Ser (S)	473	Asn (N)
Ser (S)	483	Cys (C)
Glu (E)	531	Asp (D)

### Amino acid substitutions impact glycan direct physical interaction specificity

**Fig 7:**
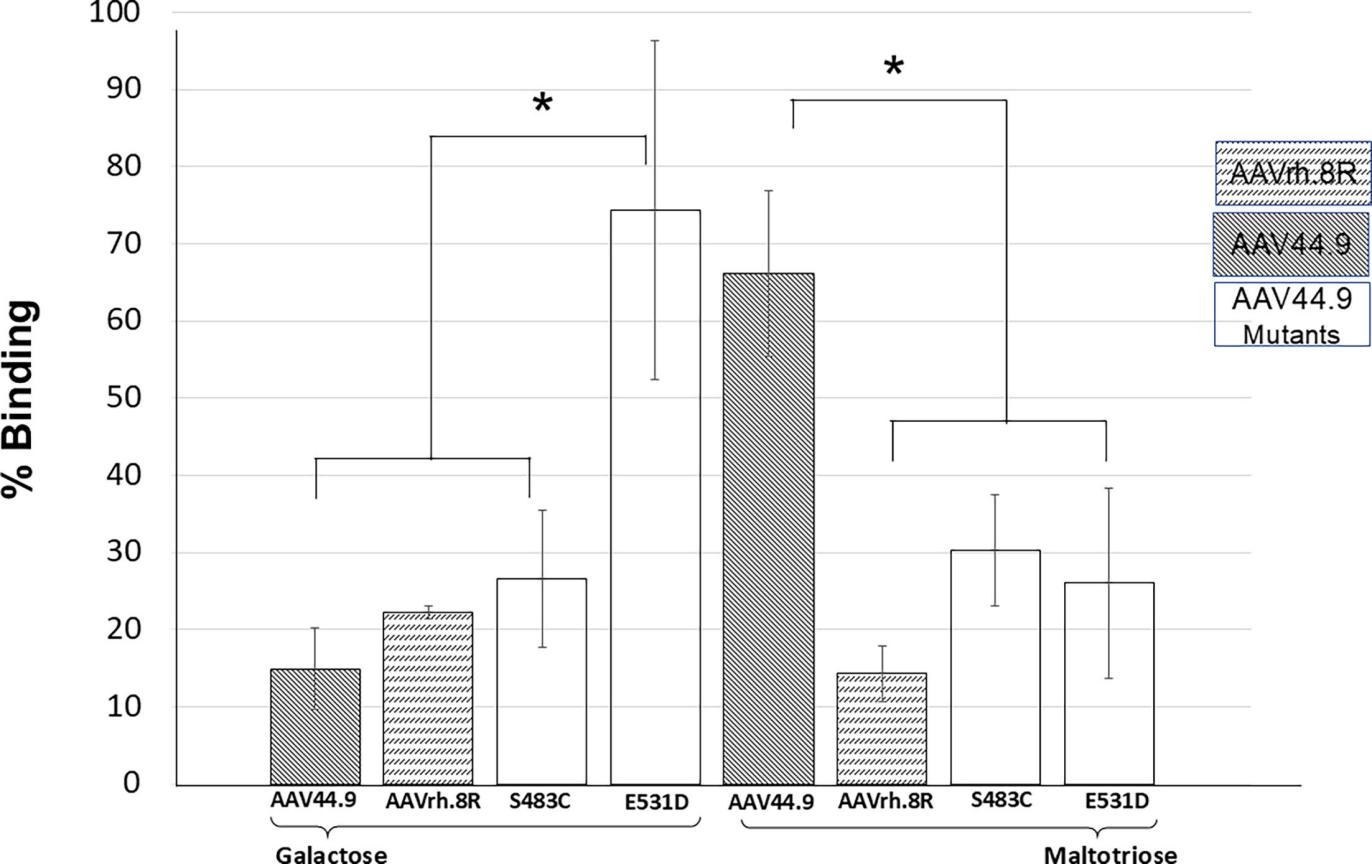
Pull-down assays were conducted with AAV44.9, AAVrh.8R, AAV44.9 S483C, and AAV44.9 E531D mutant viruses incubated with either galactose or maltotriose beads followed by washing and quantification of bound vector DNA. Results are an average of four independent experiments in triplet. *P < 0.05 was considered statistically significant.

A virus binding and pull-down assay with glycan-coated beads was developed to study physical interactions between the viruses and the glycans. AAV44.9, AAVrh.8R, S483C, and E531D mutants, respectively, were incubated with galactose- or maltotriose-coated beads. After stringent wash conditions, the viral vector that remained attached to the beads was quantified by qPCR. DNA quantification is represented as percentage of viral input recovered. These results showed that little AAV44.9 was bound and recovered from galactose-coated beads, yet significantly more AAVrh.8R was recovered (Fig. 7). Interestingly, more than 70% of mutant E531D was recovered from galactose beads. On the other hand, a high percentage of AAV44.9 was recovered from maltotriose-coated beads, whereas recovery of AAVrh.8R was less than 20% (Fig. 7). Interestingly, recovery of the S483C or E531D mutant viruses was less than 50% of that of AAV44.9 but was much higher than that of AAVrh8.R (Fig. 7).

Taken together, the data strongly support direct physical interaction between AAV44.9 and maltotriose but not with galactose, whereas for the mutants S483C, E531D, and AAV44.9, the maltotriose physical interaction is substantially decreased. In contrast, the mutant E531D exhibited a robust physical interaction with galactose. These physical interactions confirm differences in AAV44.9 and AAVrh.8R and support critical amino acid residues in cell glycan binding in these evolutionarily related virus isolates.

## DISCUSSION

Although over 200 AAV capsid sequences have been reported (Fig. S1), our understanding of the functional interaction with the host cell surface is very limited. To date, cell surface glycans have been proposed as a primary attachment receptor for a number of AAVs, and these fall into three categories: heparan sulfate proteoglycans, sialylated glycans, and terminal galactose. Sialic acid and galactose share a common synthesis pathway and are often structurally linked, with galactose being the penultimate residue in N- and O-link glycans before the terminal sialic acid. This study identifies cell surface glucose or glucosamine residues for AAV.449 binding and transduction, a new AAV-glycan interaction. Structurally, these glycans are the anti-penultimate residue in N-linked glycosylation. This property is shared with the related virus AAVrh.8R but with lower binding activity. Amino acid swapping between AAV44.9 and AAVrh.8R suggests that amino acids 483 and 531 can impact glycan binding and specificity. Based on the published cryo-EM structure for AAVrh.8R, these amino acids roughly fall in the three-fold domain, which is associated with glycan binding in AAV3, AAV6, and AAV13. This region represents a new functional domain on the surface of the AAV particles and provides a location for mutagenesis and manipulation of vector tropism.

As observed in this study, changing a single amino acid at a critical location in the AAV capsid can alter the biologic activity of the particle. Examples of this in other AAVs include AAV5 L587T, which results in the preferential binding of an acylated form of sialic acid instead of the sulfonated form preferred by the wild-type particle (20). Changing single amino acids on the capsid altered the glycan recognition *in vitro* and is reported to alter the spread of vector *in vivo* (20). Mutation of any region of the capsid may also alter immune recognition and antibody-mediated neutralization. Further studies will be required to investigate the seroprevalence of neutralizing antibodies to AAV44.9.

This manuscript describes the impact of cell surface glycans on the tropism of 44.9, specifically the importance of terminal glucose and O-linked-N-acetylglucosamine (O-GlcNAc) modification. O-GlcNAc modifications are associated with cardiac cell functions, suggesting that cardiac cells may be a good target for 44.9. O-GlcNAc is cardioprotective under acute stress (e.g., ischemia/reperfusion) (21). In propionic acidemia, which is characterized by lethal metabolic decompensation and cardiomyopathy, AAV44.9-mediated therapy showed high cardiac transgene expression (22). It is an open question whether this cardiotropic transduction is the result of changes in O-GlcNAc modifications in cardiomyocytes and will require additional investigation.

After the initial addition of a nascent N-linked glycan core to a new polypeptide, the immature core terminates with three glucose residues, creating an immature GlcMan9GlcNAc2 N-glycan in the ER (23). The composition of this core is highly conserved. Typically, these terminal glucose residues are removed and replaced by other glycans to create a mature protein. However, if the process of maturation is interrupted or the glycans are not modified further, these nascent core molecules will be released and trafficked to the cell surface, which could serve as receptors for AAV44.9. If maturation continues in the Golgi, additional modifications of N-linked core result in mature, biantennary, and complex N-glycans, which contain many branches with terminal N-acetylglucosamine residues.

Although glucose and glucosamine are often found in the core of N- and O-linked glycans and GPI-anchored proteins, they are not reported to be involved in virus infection. Glucosamine is the product of glucose and glutamine, which serves several important roles at the cell surface of fungi in the form of N-acetylglucosamine. One critical role is in the formation of the cell wall, the innermost layer of which is composed of chitin, a polymer of *β*-(1,4)-linked GlcNAc. Chitotriose is a polymer of *β*-(1,4)-linked GlcNAc found on the endosomal protein gp96 and utilizes its activity for transcytosis across barrier epithelia (24). Although the binding of AAV44.9 to the terminal glucose glycan #466 was weak compared to BAAV interaction with chitotriose, the coefficient of variance (%CV = 4) suggests high specificity. Furthermore, the binding to the terminal glucose likely also has a structural component as there were two other glycans on the array that have a terminal Glca1-4; however, they were ranked much farther down on the array (i.e., the closest was 195 ranked #40 and 196 ranked #245). Based on the original data from the array, it is surprising that AAV44.9 does not also interact with galactose like AAV9. This may be partially explained by the fact that the structurally glucose and galactose differ in the orientation of the hydroxyl group attached to the C4 carbon of the hexose ring. Furthermore, the average RFU is not significant for the second highest RFU glycan #539, and the overall %CV is high for #539 compared with #466.

In summary, our finding supports an important role of cell surface glycans in the binding and infection of AAVs. Unlike other virus families, AAVs do not rely on one type of glycan but utilize a wide array of interactions. As we identify more of these types of interactions and map them to the surface of capsids, a better picture may emerge regarding the molecular mechanism this interaction plays in the internalization and uncoating of the AAV particles in a permissive cell. Finally, by identifying this critical interaction, it opens the possibility to alter the cell surface and make them more permissive to vector uptake and transduction.

## MATERIALS AND METHODS

### Cell culture

African green monkey kidney Cos and (The American Type Culture Collection [ATCC], Manassas, VA) and 293T cells (human embryonic kidney cells) were cultured in Dulbecco’s modified Eagle’s medium (DMEM) supplemented with 10% fetal bovine serum (FBS; HyClone, Logan, UT), 2 mM L-glutamine, 100 U of penicillin/mL, and 0.1 mg of streptomycin/mL (Invitrogen, Carlsbad, CA). Cells were maintained at 37°C under a 5% CO_2_ humidified atmosphere. Pro 5 Cho cells (ATCC) were grown in DMEM/F12 and supplemented as above.

### Protease and glycosidase treatments

#### Trypsin treatment

Cos cells were cultured in a 15-cm-diameter dish until they were 80% confluent. Cells were then washed twice with 1× PBS, scraped, and resuspended in 10 mL of 1× PBS and incubated with 0.05% trypsin (Life Technologies) or mock medium (control) for 15 min at 37°C. Cells were then centrifuged at 500 g for 5 min and washed twice with medium without serum and seeded at a density of 1 × 10^4^ cells/well in a 96-well dish. Following cell attachment (~3 h) at 37°C, cells were incubated with AAV44.9 or AAV5 (1.10^4^ particles/cell) carrying a CMV-*Gaussia* reporter for 2 h. After removing the vector by washing, transduction efficiency was tested 24 h later by the *Gaussia* luciferase assay.

#### Neuraminidase treatment

Cos cells were pretreated with or without neuraminidase in DMEM supplemented with 5% FCS for 2 h. After washing, cells were incubated with AAV44.9 or AAV5 CMV-*Gaussia*. Two hours later, remaining particles were removed by washing, and the cells were incubated for 24 h followed by the *Gaussia* luciferase assay.

#### Tunicamycin treatments

For transduction experiments, Cos cells were seeded in 96-well plates (8.10^3^/well). After cell attachment, the cultures were incubated overnight with or without tunicamycin (0.05 µg/mL) in serum-free DMEM. Sixteen hours later, recombinant AAV44.9 or AAV5 CMV-*Gaussia* was added (1.10^4^ particles/cell) with or without tunicamycin (Ab120296) for another 2 h of incubation. Finally, cells were washed and incubated in DMEM 10% FBS media. Transduction efficiency was tested 24 h later by the *Gaussia* luciferase assay. In the tunicamycin internalization assay, Cos-1 cells were plated in 12-well plates (1 × 10^5^ cells/well) in 1 mL of complete media. Once cells attached, either control or 0.05 µg/mL of tunicamycin in serum-free media was added overnight. After 16 h, recombinant AAV44.9 or AAV5 was added (1.10^4^ particles/cell) in serum-free media in the presence or absence of tunicamycin for 5 h to allow internalization. Cells were washed gently with 1× PBS and then treated with 800 µL trypsin (0.05%) to detach and remove only the surface-bound virions but not the internalized virions. Cells were transferred to microcentrifuge tubes and pelleted by 500 *g* centrifugation for 5 min at room temperature. Trypsin was carefully removed by washing three times with 400 µL 1× PBS. After the final wash, the cell pellet was resuspended in 200 µL 1× PBS. DNA was isolated using the QIAGEN DNeasy Blood and Tissue Kit (Qiagen, Germany), following the manufacturer’s instruction. Finally, the DNA was eluted from columns in 50-µL molecular-grade water, and the genomes of cell-associated virions were determined by quantitative real-time PCR using specific primers to the *Gaussia* CMV promoter.

#### N-acetylglucosaminidase treatment

Transduction: Cos cells plated in a 96-well plate were incubated for 30 min at 37°C with 2/3 serial dilution in DMEM with N-acetylglucosaminidase. After cell washing, the recombinant DNA particles were added and incubated for 2 h at 37°C. After recombinant DNA removal and cell washes, transduction values were quantified 48 h later by the *Gaussia* luciferase assay. Binding: Cos cells plated in 12-well plates were treated with 1.2 U/mL of N-acetylglucosaminidase as above. After washing, cells were chilled at 4°C and incubated for 1 h with recombinant. After removing the unbounded particles by washing at 4°C, recombinant DNA was quantified by qPCR from DNA cell extracts as above.

### Glycan treatment for viral transduction competition or binding

#### Heparin treatment 

Exponentially growing Cos cells were plated at a density of 1.10^4^ per well in a flat-bottom 96-well plate. AAV vectors were preincubated with either media alone (control) or with 20 µg heparin/mL (Sigma H-3149). After 1-h incubation at room temperature, the mixture was added to cells and incubated for another hour at 37°C. Then, the mixture was removed, and the cells were rinsed three times with the medium. Forty-eight hours later, the *Gaussia* luciferase activity was quantified (Pierce #16160).

#### N-acetylglucosamine, maltotriose, or galactose treatments

Prior to the glycan treatment, the amount of vector was optimized to yield ~2.10^5^ relative light units (RLU) in a *Gaussia* luciferase 48 h post incubation on Cos cells. To test the glycan effect on transduction, serial dilutions of glycans and vector were prepared on separate 96-well plates, and after 1-h incubation at room temperature, the recombinants containing serial dilution glycans were transferred to a 96-well plate with Cos cells plated. Cell/mixture was incubated for 2 h and then removed. Cells were washed three times and left for 48 h in the medium with serum, and then the *Gaussia* luciferase assay was performed.

### Production and purification of AAV recombinants

Recombinants AAV2, AAV5, AAV9, AAVrh.8R, and AAV44.9 wt and four mutants were obtained using a three (AAV2, AAV9, and AAVrh.8R) or four-plasmid (AAV5, AAV44.9, and mutants) transfection procedures as previously described (13). Human embryonic kidney 293T cells were obtained from the ATCC (Manassas, VA, USA). 293T cells were grown at 37°C under a 5% CO_2_ humidified atmosphere in DMEM supplemented with 10% FBS, 2 mM l-glutamine, 100 U of penicillin/mL, and 0.1 mg of streptomycin/mL. Briefly, 293T cells were transfected by calcium phosphate with three or four plasmids as follows: an adenovirus helper plasmid (pAd12) containing VA RNA and coding the E2 and E4 proteins, one AAV plasmid containing rep and cap or two AAV plasmids containing either the AAV rep or the AAV capsid gene, and a vector plasmid including the AAV2 inverted terminal repeats flanking the reporter gene expression cassette. The cells were harvested 48 h post transfection, and a crude viral lysate was obtained after one freeze-thaw cycle. The lysate was treated with 0.5% DOC and 100 U/mL DNase (Benzonase) for 30 min at 37°C. The vector particles present in the clarified lysate (obtained by further low-speed centrifugation) were further purified by CsCl gradient ultracentrifugation, and the vector titer was determined by quantitative real-time PCR (Applied Biosystems, Foster City, CA, USA). The vector doses were dialyzed against 0.9% NaCl using Slide-A-Lyzer 10K cassettes (Thermo Fisher Scientific). Vector was concentrated using a centrifugal filter unit (Amicon Ultra). AAVr particles were quantified by qPCR. One microliter of diluted recombinant preparation was added to a PCR containing 1× SYBR Green Master Mix (ABI) and 0.25 pmol/µL forward and reverse primers. Amplification was detected using QuantStudio 3 (AB). Specific primers for AAV CBA-*Gaussia* or AAV CMV were used: HGLuc. forward 5′-CACGCCCAAGATGAAGAAGT-3′, HGLuc. reverse 5′-GAACCCAGGAATCTCAGGAATG-3′, CMV forward 5′-CATCTACGTATTAGTCATCGCTATTACCAT-3′, and CMV reverse 5′-TGGAAATCCCCGTGAGTCA-3′. Following denaturation at 96°C for 10 min, cycling conditions were 96°C for 15 s and 60°C for 1 min for 40 cycles. The viral DNA in each sample was quantified by comparing the fluorescence profiles with a set of DNA standards.

### Glycan microarray

AAV44.9-HA particles were produced as described above and concentrated to a titer of ∼1 × 10^14^ vg/mL. The concentrated virus was then dialyzed into a Tris buffer (Tris pH 7.5 with 150 mM NaCl) and used to probe a printed glycan array (PA V2) following procedures developed by cores D and H of the Consortium for Functional Glycomics (CFG; an NIH National Institute of General Medical Sciences initiative: for identifying specific carbohydrate-binding partners for protein and viral lectins (25). Briefly, a printed slide containing ~600 sialylated and non-sialylated glycans with different linkages and modifications was previously useful in characterizing AAV virus particle:carbohydrate interactions. In this study, a similar slide was incubated with AAV44.9 particles (at 200 µg/mL) and overlaid with a FITC-conjugated anti-HA tag (Molecular Probes Invitrogen) at 5 µg/mL. The fluorescence intensity was detected using a ScanArray 5000 (PerkinElmer Inc.) confocal scanner. The image was analyzed using the IMAGENE image analysis software (Bio-Discovery, El Segundo, CA). The data were plotted using Microsoft Excel software and are included as Table S1 in the supplemental material.

### Plasmids

AAV44.9-HA plasmid containing the capsid DNA gene with the HA tag (YPYDVPDYA) added to AAV44.9 after amino acid 137 in the VP1 was obtained by custom DNA synthesis (VectorBuilder). Mutations in AAV44.9 were generated using the Site-Directed Mutagenesis Kit (Stratagene) with the following sets of primers:

Mutant T179S, FWR 5′-CAGACTGGCGACTCAGAGTCAGTCC-3′, REV, 5′-GGACTGACTCTGAGTCGCCAGTCTG-3′. Mutant S473N, FWR 5′-CCTAGCTCAATGGCCAACCAGGCTAGAAACTG-3′, REV 5′-CAGTTTCTAGCCTGGTTGGCCATTGAGCTAGG-3′. Mutant S483C, 5′-GGTGCCCGGACCGTGCTACCGGCAGCAG-3′, REV, 5′-CTGCTGCCGGTAGCACGGTCCGGGCACC-3′. Mutant E531D, FWR 5′-CACAAGGATGACGACGACCGCTTCTTCCC-3′, REV, 5′-GGGAAGAAGCGGTCGTCGTCATCCTTGTG-3′.

### *Gaussia* luciferase assay

Ten to twenty milliliters of supernatant medium of each well was mixed with 200-mL substrate buffer, GLuc Glow Assay (NanoLight Technology), and immediately quantified for luminescence using a multi-well reader luminometer Victor X2 (PerkinElmer).

### AAV reporter titration

Each AAV containing CAG-*Gaussia* luciferase reporter gene was first tested on Cos cells, (1.5.10^4^ plated in 96-well plates) the day before experiment, to achieve ~1 0.10^5^ RLU in a *Gaussia* luciferase assay, after 2 h virus cell incubation. The luciferase assay was performed 48 h post incubation.

### Cho Pro5 and mutant cell line transduction assay

Cho Pro5 and three mutant cell lines were incubated with AAV5, AAV9, or AAV44.9 CMV-*Gaussia* luciferase, respectively, for 2 h at 4°C. Cells were washed with media at 4°C. After 48 h incubation at 37°C, *Gaussia* luciferase was performed.

### Preparation of glycan beads

Glycan bound beads of interest were purchased from a commercial supplier or created by coupling glycan to CNBr-activated Sepharose (Sigma) beads following the manufacturer’s recommendations. Glycan beads are stored in 0.02% sodium azide or 20% ethanol. The beads were washed several times with 3× volume of wash buffer (1 mM MgCl_2_/0.5× PBS) prior to use.

### Pull-down binding assay

One hundred fifty microliters of each glycan bead was incubated with virus at 4°C for 1 h. The tubes were gently vortexed and mixed every 10 min during the incubation period. Afterward, the beads were washed four times with wash buffer to remove any unbound virus. Finally, all the buffer was removed, and the beads were collected for downstream analysis.

### DNA extraction—complex recovery to detect single-particle interactions

To each Eppendorf tube, 40 µL phenol:chloroform and 40 µL of water were added. The tubes were vortexed for 5 min, followed by benchtop centrifugation. Then, 10 µL of the top aqueous phase containing DNA was carefully pipetted out and transferred to a fresh PCR tube.

### Quantification of DNA

The extracted DNA was quantified using qPCR against a prepared standard using AAV2 GFP CMV DNA. Relative CMV primers were synthesized and used alongside Power Syber Green Master Mix. The remaining DNA was stored in −20°C until further analysis.

### Data analysis

Statistical analysis was performed to determine standard deviation and significance between different experimental conditions. A *P*-value less than 0.05 was considered statistically significant.

### Statistical analysis

Data were analyzed using Excel software. Continuous variables with normal distributions are shown as mean ± SD.

## SUPPLEMENTAL MATERIAL

Fig. S1 jvi.00254-25-s0001.pdf AAV phylogenetic tree.

Fig. S2 jvi.00254-25-s0002.pdf Amino acid sequence alignment of AAV44.9 and AAVrh.8R capsid proteins.

Table S1 jvi.00254-25-s0003.pdf Glycan microarray data.

ASM does not own the copyrights to Supplemental Material that may be linked to, or accessed through, an article. The authors have granted ASM a non-exclusive, world-wide license to publish the Supplemental Material files. Please contact the corresponding author directly for reuse.
