# Supplementary material for: Terminal glucose as a receptor for adeno-associated virus 44.9
Source: J Virol. 2026 Mar 23;100(4):e00254-25. doi: 10.1128/jvi.00254-25 (PMC13098211; doi:10.1128/jvi.00254-25)
Supplement: Fig. S1 — AAV phylogenetic tree. [file jvi.00254-25-s0001.pdf]

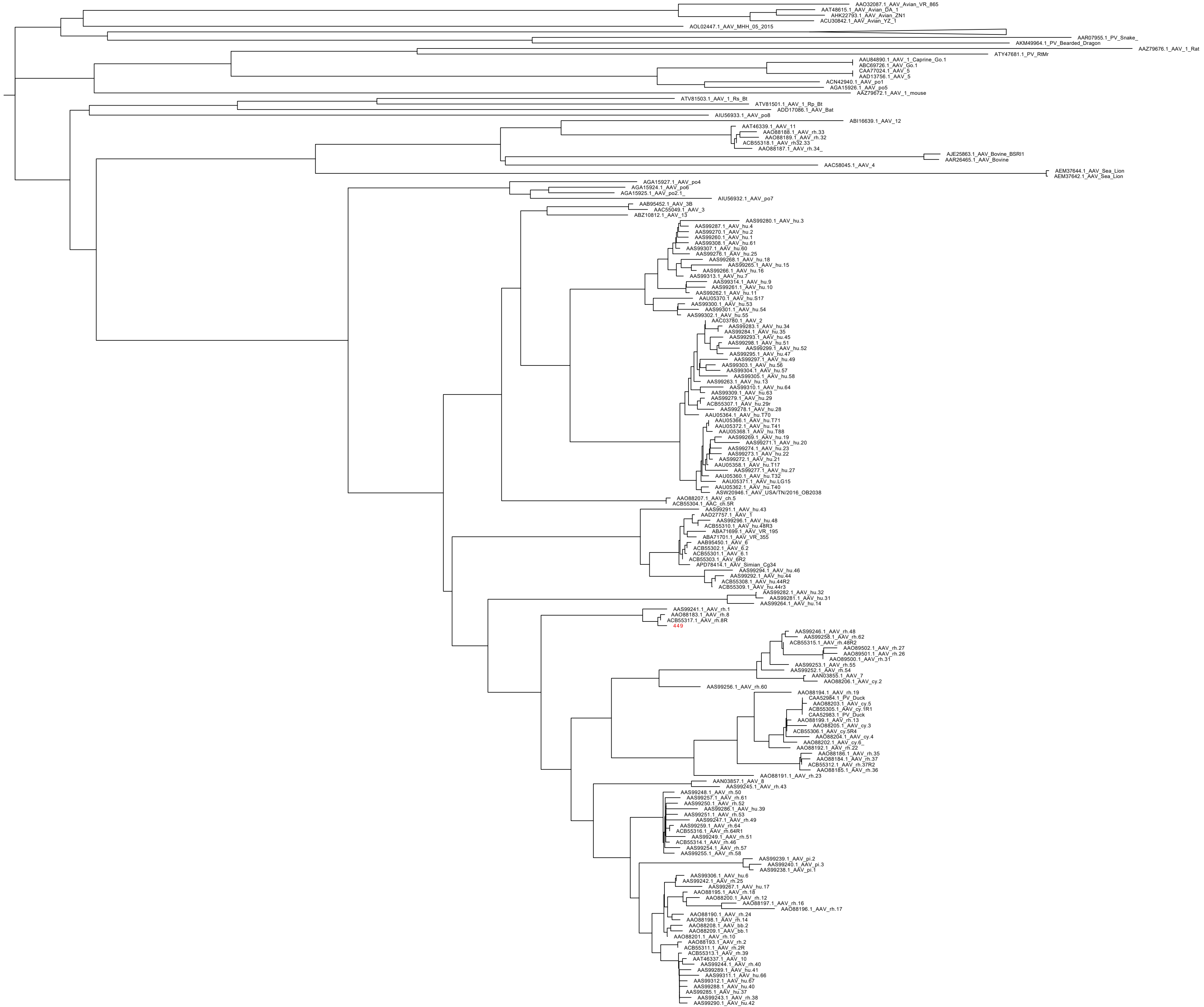
AAO32087.1_AAV_Avian_VR_865
AAT48615.1_AAV_Avian_DA_1
AHK22793.1_AAV_Avian_ZN1
ACU30842.1_AAV_Avian_YZ_1
AOL02447.1_AAV_MHH_05_2015
AAR07955.1_PV_Snake_
AKM49964.1_PV_Bearded_Dragon
AAZ79676.1_AAV_1_Rat
ATY47681.1_PV_RtMr
AAU84890.1_AAV_1_Caprine_Go.1
ABC69726.1_AAV_Go.1
CAA77024.1_AAV_5
AAD13756.1_AAV_5
ACN42940.1_AAV_po1
AGA15926.1_AAV_po5
AAZ79672.1_AAV_1_mouse
ATV81503.1_AAV_1_Rs_Bt
ATV81501.1_AAV_1_Rp_Bt
ADD17086.1_AAV_Bat
AIU56933.1_AAV_po8
ABI16639.1_AAV_12
AAT46339.1_AAV_11
AAO88188.1_AAV_rh.33
AAO88189.1_AAV_rh.32
ACB55318.1_AAV_rh32.33
AAO88187.1_AAV_rh.34_
AJE25863.1_AAV_Bovine_BSRI1
AAR26465.1_AAV_Bovine
AAC58045.1_AAV_4
AEM37644.1_AAV_Sea_Lion
AEM37642.1_AAV_Sea_Lion
AGA15927.1_AAV_po4
AGA15924.1_AAV_po6
AGA15925.1_AAV_po2.1_
AIU56932.1_AAV_po7
AAB95452.1_AAV_3B
AAC55049.1_AAV_3
ABZ10812.1_AAV_13
AAS99280.1_AAV_hu.3
AAS99287.1_AAV_hu.4
AAS99270.1_AAV_hu.2
AAS99260.1_AAV_hu.1
AAS99308.1_AAV_hu.61
AAS99307.1_AAV_hu.60
AAS99276.1_AAV_hu.25
AAS99268.1_AAV_hu.18
AAS99265.1_AAV_hu.15
AAS99266.1_AAV_hu.16
AAS99313.1_AAV_hu.7
AAS99314.1_AAV_hu.9
AAS99261.1_AAV_hu.10
AAS99262.1_AAV_hu.11
AAU05370.1_AAV_hu.S17
AAS99300.1_AAV_hu.53
AAS99301.1_AAV_hu.54
AAS99302.1_AAV_hu.55
AAC03780.1_AAV_2
AAS99283.1_AAV_hu.34
AAS99284.1_AAV_hu.35
AAS99293.1_AAV_hu.45
AAS99298.1_AAV_hu.51
AAS99299.1_AAV_hu.52
AAS99295.1_AAV_hu.47
AAS99297.1_AAV_hu.49
AAS99303.1_AAV_hu.56
AAS99304.1_AAV_hu.57
AAS99305.1_AAV_hu.58
AAS99263.1_AAV_hu.13
AAS99310.1_AAV_hu.64
AAS99309.1_AAV_hu.63
AAS99279.1_AAV_hu.29
ACB55307.1_AAV_hu.29r
AAS99278.1_AAV_hu.28
AAU05364.1_AAV_hu.T70
AAU05366.1_AAV_hu.T71
AAU05372.1_AAV_hu.T41
AAU05368.1_AAV_hu.T88
AAS99269.1_AAV_hu.19
AAS99271.1_AAV_hu.20
AAS99274.1_AAV_hu.23
AAS99273.1_AAV_hu.22
AAS99272.1_AAV_hu.21
AAU05358.1_AAV_hu.T17
AAS99277.1_AAV_hu.27
AAU05360.1_AAV_hu.T32
AAU05371.1_AAV_hu.LG15
AAU05362.1_AAV_hu.T40
ASW20946.1_AAV_USA/TN/2016_OB2038
AAO88207.1_AAV_ch.5
ACB55304.1_AAC_ch.5R
AAS99291.1_AAV_hu.43
AAD27757.1_AAV_1
AAS99296.1_AAV_hu.48
ACB55310.1_AAV_hu.48R3
ABA71699.1_AAV_VR_195
ABA71701.1_AAV_VR_355
AAB95450.1_AAV_6
ACB55302.1_AAV_6.2
ACB55301.1_AAV_6.1
ACB55303.1_AAV_6R2
APD78414.1_AAV_Simian_Cg34
AAS99294.1_AAV_hu.46
AAS99292.1_AAV_hu.44
ACB55308.1_AAV_hu.44R2
ACB55309.1_AAV_hu.44r3
AAS99282.1_AAV_hu.32
AAS99281.1_AAV_hu.31
AAS99264.1_AAV_hu.14
AAS99241.1_AAV_rh.1
AAO88183.1_AAV_rh.8
ACB55317.1_AAV_rh.8R
449
AAS99246.1_AAV_rh.48
AAS99258.1_AAV_rh.62
ACB55315.1_AAV_rh.48R2
AAO89502.1_AAV_rh.27
AAO89501.1_AAV_rh.26
AAO89500.1_AAV_rh.31
AAS99253.1_AAV_rh.55
AAS99252.1_AAV_rh.54
AAN03855.1_AAV_7
AAO88206.1_AAV_cy.2
AAS99256.1_AAV_rh.60
AAO88194.1_AAV_rh.19
CAA52984.1_PV_Duck
AAO88203.1_AAV_cy.5
ACB55305.1_AAV_cy.1R1
CAA52983.1_PV_Duck
AAO88199.1_AAV_rh.13
AAO88205.1_AAV_cy.3
ACB55306.1_AAV_cy.5R4
AAO88204.1_AAV_cy.4
AAO88202.1_AAV_cy.6_
AAO88192.1_AAV_rh.22
AAO88186.1_AAV_rh.35
AAO88184.1_AAV_rh.37
ACB55312.1_AAV_rh.37R2
AAO88185.1_AAV_rh.36
AAO88191.1_AAV_rh.23
AAN03857.1_AAV_8
AAS99245.1_AAV_rh.43
AAS99248.1_AAV_rh.50
AAS99257.1_AAV_rh.61
AAS99250.1_AAV_rh.52
AAS99286.1_AAV_hu.39
AAS99251.1_AAV_rh.53
AAS99247.1_AAV_rh.49
AAS99259.1_AAV_rh.64
ACB55316.1_AAV_rh.64R1
AAS99249.1_AAV_rh.51
ACB55314.1_AAV_rh.46
AAS99254.1_AAV_rh.57
AAS99255.1_AAV_rh.58
AAS99239.1_AAV_pi.2
AAS99240.1_AAV_pi.3
AAS99238.1_AAV_pi.1
AAS99306.1_AAV_hu.6
AAS99242.1_AAV_rh.25
AAS99267.1_AAV_hu.17
AAO88195.1_AAV_rh.18
AAO88200.1_AAV_rh.12
AAO88197.1_AAV_rh.16
AAO88196.1_AAV_rh.17
AAO88190.1_AAV_rh.24
AAO88198.1_AAV_rh.14
AAO88208.1_AAV_bb.2
AAO88209.1_AAV_bb.1
AAO88201.1_AAV_rh.10
AAO88193.1_AAV_rh.2
ACB55311.1_AAV_rh.2R
ACB55313.1_AAV_rh.39
AAT46337.1_AAV_10
AAS99244.1_AAV_rh.40
AAS99289.1_AAV_hu.41
AAS99311.1_AAV_hu.66
AAS99312.1_AAV_hu.67
AAS99288.1_AAV_hu.40
AAS99285.1_AAV_hu.37
AAS99243.1_AAV_rh.38
AAS99290.1_AAV_hu.42

Fig. 2 S

AAV44.9 Cap has 99% Identity with the Neurotrophic AAVRh8

```
AAV44.9 1    MAADGYLPDWLEDNLSEGIREWWDLKPGAPKPKANQQKQDDGRGLVLPGYKYLGPFNGLD  60
             MAADGYLPDWLEDNLSEGIREWWDLKPGAPKPKANQQKQDDGRGLVLPGYKYLGPFNGLD
AAVRh8  1    MAADGYLPDWLEDNLSEGIREWWDLKPGAPKPKANQQKQDDGRGLVLPGYKYLGPFNGLD  60

AAV44.1 61   KGEPVNAADAAALEHDKAYDQQLKAGDNPYLRYNHADAEFQERLQEDTSFGGNLGRAVFQ  120
             KGEPVNAADAAALEHDKAYDQQLKAGDNPYLRYNHADAEFQERLQEDTSFGGNLGRAVFQ
AAVRh8  61   KGEPVNAADAAALEHDKAYDQQLKAGDNPYLRYNHADAEFQERLQEDTSFGGNLGRAVFQ  120

AAV44.1 121  AKKRVLEPLGLVEEGAKTAPGKKRPVEQSPQEPDSSSGIGKTGQQPAKKRLNFGQTGDTE  180
             AKKRVLEPLGLVEEGAKTAPGKKRPVEQSPQEPDSSSGIGKTGQQPAKKRLNFGQTGD+E
AAVRh8  121  AKKRVLEPLGLVEEGAKTAPGKKRPVEQSPQEPDSSSGIGKTGQQPAKKRLNFGQTGDSE  180

AAV44.1 181  SVPDPQPLGEPPAAPSGLGPNTMASGGGAPMADNNEGADGVGNSSGNWHCDSTWLGDRVI  240
             SVPDPQPLGEPPAAPSGLGPNTMASGGGAPMADNNEGADGVGNSSGNWHCDSTWLGDRVI
AAVRh8  181  SVPDPQPLGEPPAAPSGLGPNTMASGGGAPMADNNEGADGVGNSSGNWHCDSTWLGDRVI  240

AAV44.1 241  TTSTRTWALPTYNNHLYKQISNGTSGGSTNDNTYFGYSTPWGYFDFNRFHCHFSPRDWQR  300
             TTSTRTWALPTYNNHLYKQISNGTSGGSTNDNTYFGYSTPWGYFDFNRFHCHFSPRDWQR
AAVRh8  241  TTSTRTWALPTYNNHLYKQISNGTSGGSTNDNTYFGYSTPWGYFDFNRFHCHFSPRDWQR  300

AAV44.1 301  LINNNWGFRPKRLNFKLFNIQVKEVTTNEGTKTIANNLTSTVQVFTDSEYQLPYVLGSAH  360
             LINNNWGFRPKRLNFKLFNIQVKEVTTNEGTKTIANNLTSTVQVFTDSEYQLPYVLGSAH
AAVRh8  301  LINNNWGFRPKRLNFKLFNIQVKEVTTNEGTKTIANNLTSTVQVFTDSEYQLPYVLGSAH  360

AAV44.1 361  QGCLPPFPADVFMVPQYGYLTLNNGSQALGRSSFYCLEYFPSQMLRTGNNFQFSYTFEDV  420
             QGCLPPFPADVFMVPQYGYLTLNNGSQALGRSSFYCLEYFPSQMLRTGNNFQFSYTFEDV
AAVRh8  361  QGCLPPFPADVFMVPQYGYLTLNNGSQALGRSSFYCLEYFPSQMLRTGNNFQFSYTFEDV  420

AAV44.1 421  PFHSSYAHSQSLDRLMNPLIDQYLYYLVRTQTTGTGGTQTLAFSQAGPSSMASQARNWVP  480
             PFHSSYAHSQSLDRLMNPLIDQYLYYLVRTQTTGTGGTQTLAFSQAGPSSMA+QARNWVP
AAVRh8  421  PFHSSYAHSQSLDRLMNPLIDQYLYYLVRTQTTGTGGTQTLAFSQAGPSSMANQARNWVP  480

AAV44.1 481  GPSYRQQRVSTTTNQNNNSNFAWTGAAKFKLNGRDSLMNPGVAMASHKDDEDRFFPSSGV  540
             GP YRQQRVSTTTNQNNNSNFAWTGAAKFKLNGRDSLMNPGVAMASHKDD+DRFFPSSGV
AAVRh8  481  GPCYRQQRVSTTTNQNNNSNFAWTGAAKFKLNGRDSLMNPGVAMASHKDDDDRFFPSSGV  540

AAV44.1 541  LIFGKQGAGNDGVDYSQVLITDEEEIKATNPVATEEYGAVAINNQAANTQAQTGLVHNQG  600
             LIFGKQGAGNDGVDYSQVLITDEEEIKATNPVATEEYGAVAINNQAANTQAQTGLVHNQG
AAVRh8  541  LIFGKQGAGNDGVDYSQVLITDEEEIKATNPVATEEYGAVAINNQAANTQAQTGLVHNQG  600

AAV44.1 601  VIPGMVWQNRDVYLQGPIWAKIPHTDGNFHPSPLMGGFGLKHPPPQILIKNTPVPADPPL  660
             VIPGMVWQNRDVYLQGPIWAKIPHTDGNFHPSPLMGGFGLKHPPPQILIKNTPVPADPPL
AAVRh8  601  VIPGMVWQNRDVYLQGPIWAKIPHTDGNFHPSPLMGGFGLKHPPPQILIKNTPVPADPPL  660

AAV44.1 661  TFNQAKLNSFITQYSTGQVSVEIEWELQKENSKRWNPEIQYTSNYYKSTNVDFAVNTEGV  720
             TFNQAKLNSFITQYSTGQVSVEIEWELQKENSKRWNPEIQYTSNYYKSTNVDFAVNTEGV
AAVRh8  661  TFNQAKLNSFITQYSTGQVSVEIEWELQKENSKRWNPEIQYTSNYYKSTNVDFAVNTEGV  720

AAV44.1 721  YSEPRPIGTRYLTRNL  736
             YSEPRPIGTRYLTRNL
AAVRh8  721  YSEPRPIGTRYLTRNL  736
```

179 S→T

IV

473 N→S

V

483 C→S

531 D→E

Fig. 1A

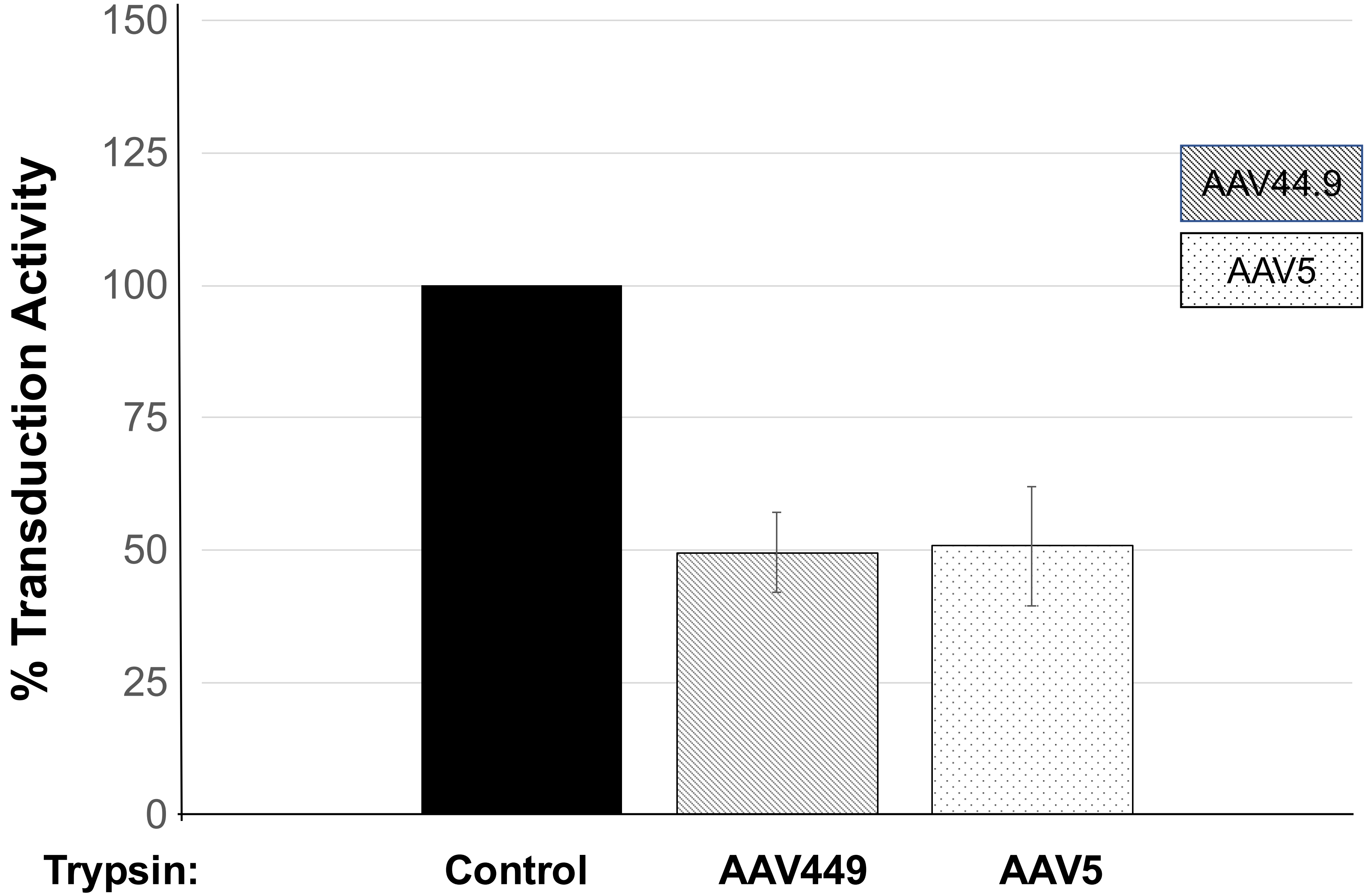
% Transduction Activity
150
125
100
75
50
25
0
AAV44.9
AAV5
Trypsin:
Control
AAV449
AAV5

Fig. 1B

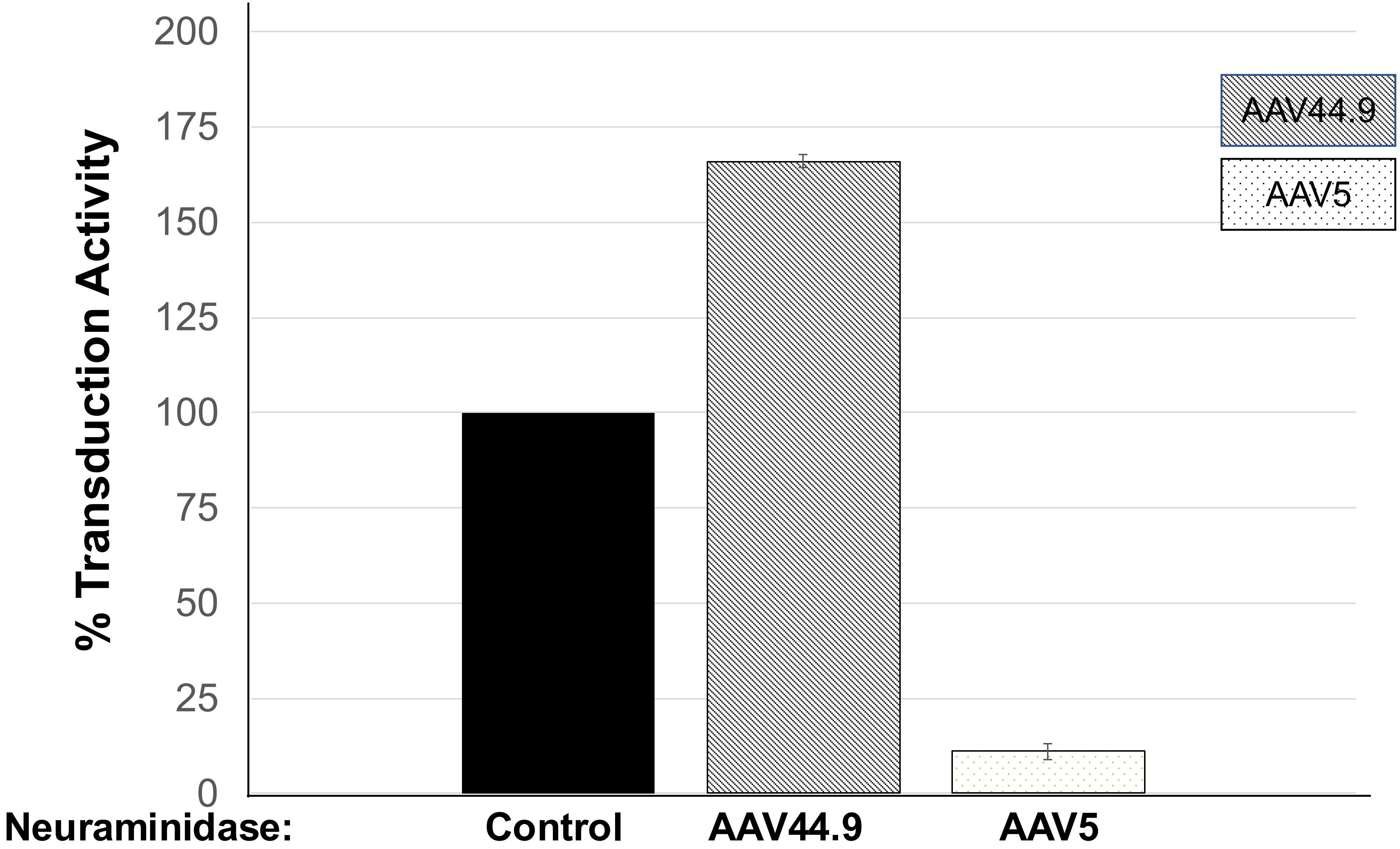

200
175
150
125
100
75
50
25
0
% Transduction Activity
AAV44.9
AAV5
Neuraminidase:
Control
AAV44.9
AAV5

Fig. 1C

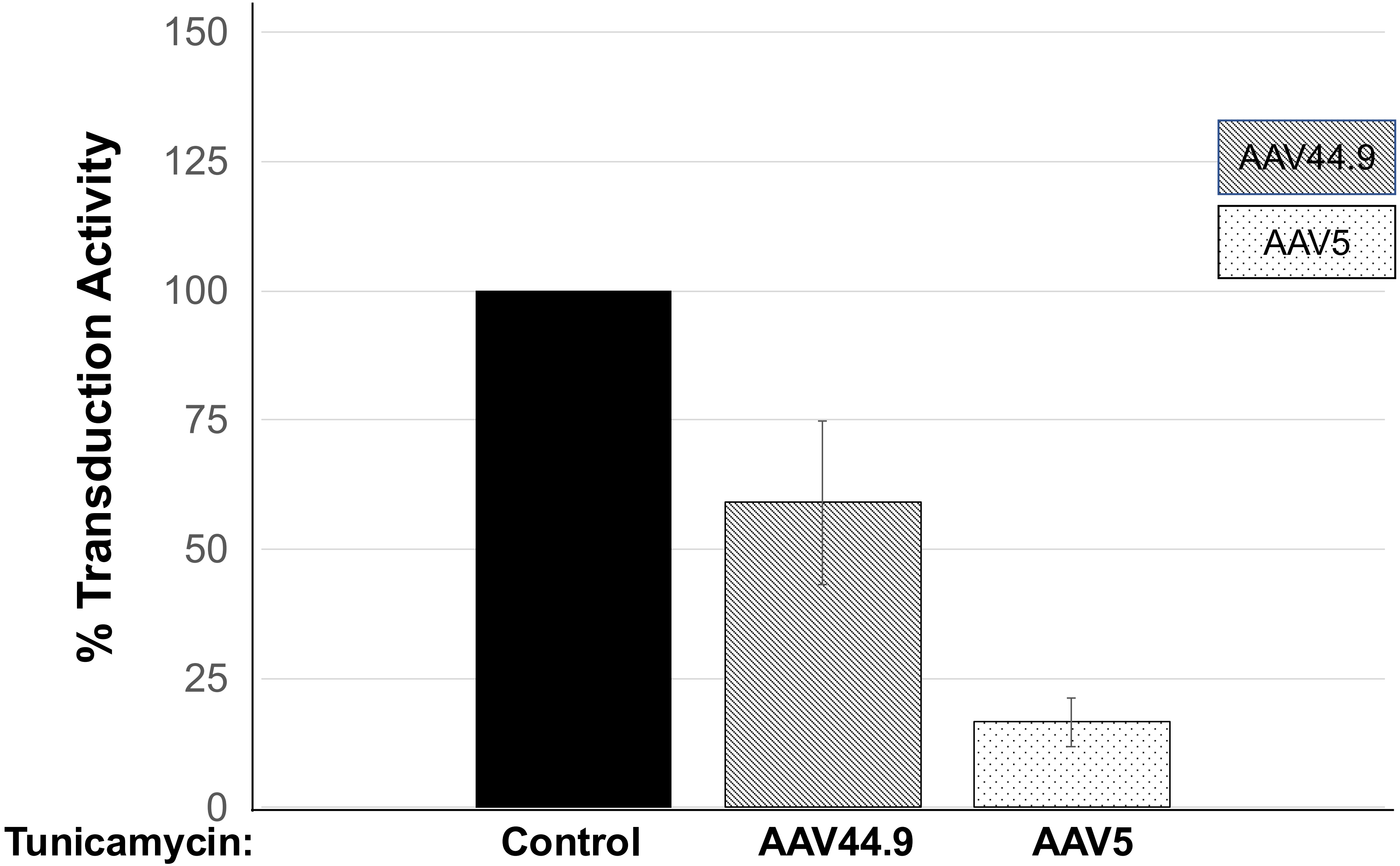

150
125
100
75
50
25
0
% Transduction Activity
AAV44.9
AAV5
Tunicamycin:
Control
AAV44.9
AAV5

Fig. 1D

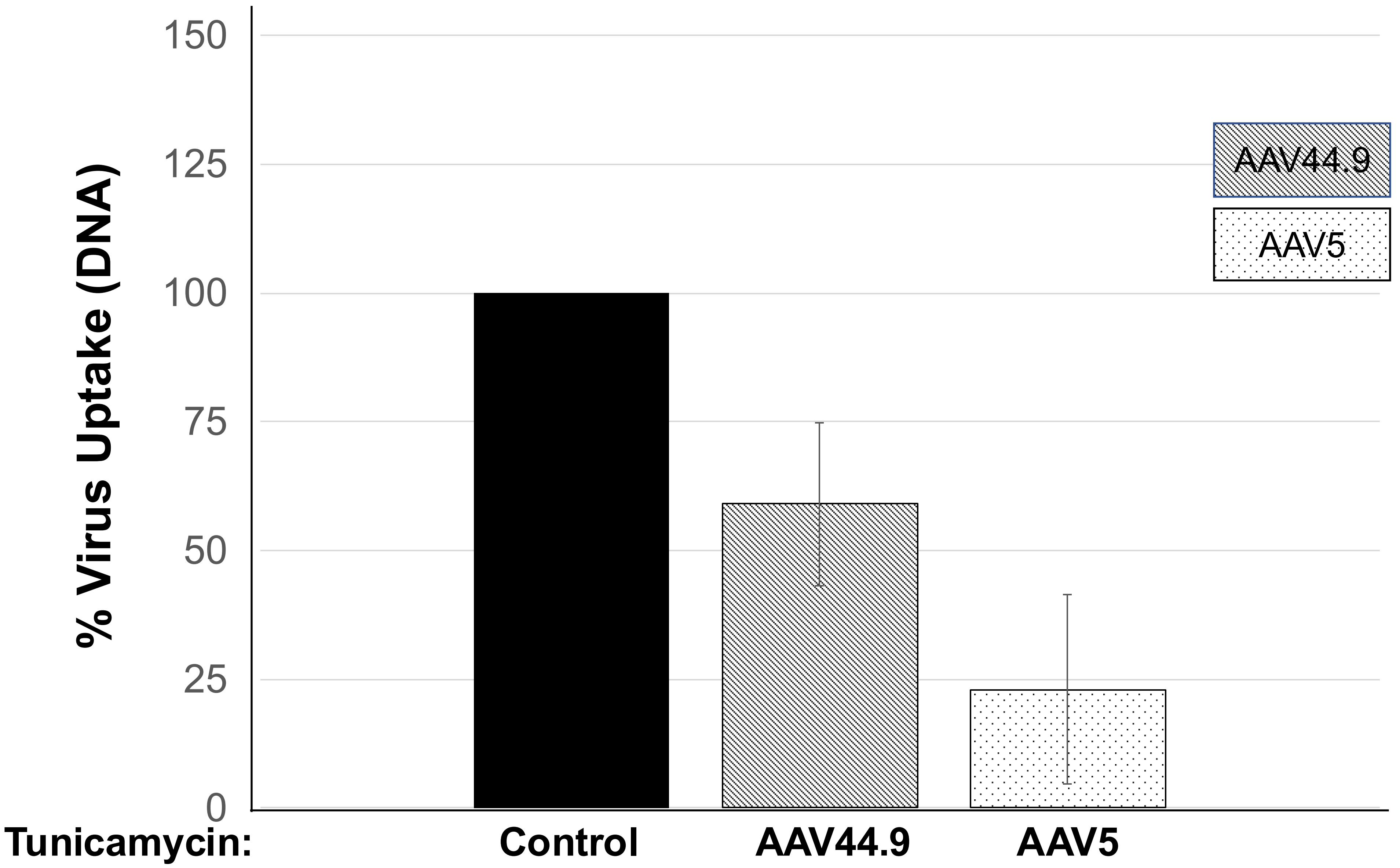
150
125
100
75
50
25
0
% Virus Uptake (DNA)
AAV44.9
AAV5
Tunicamycin:
Control
AAV44.9
AAV5

Fig. 1E

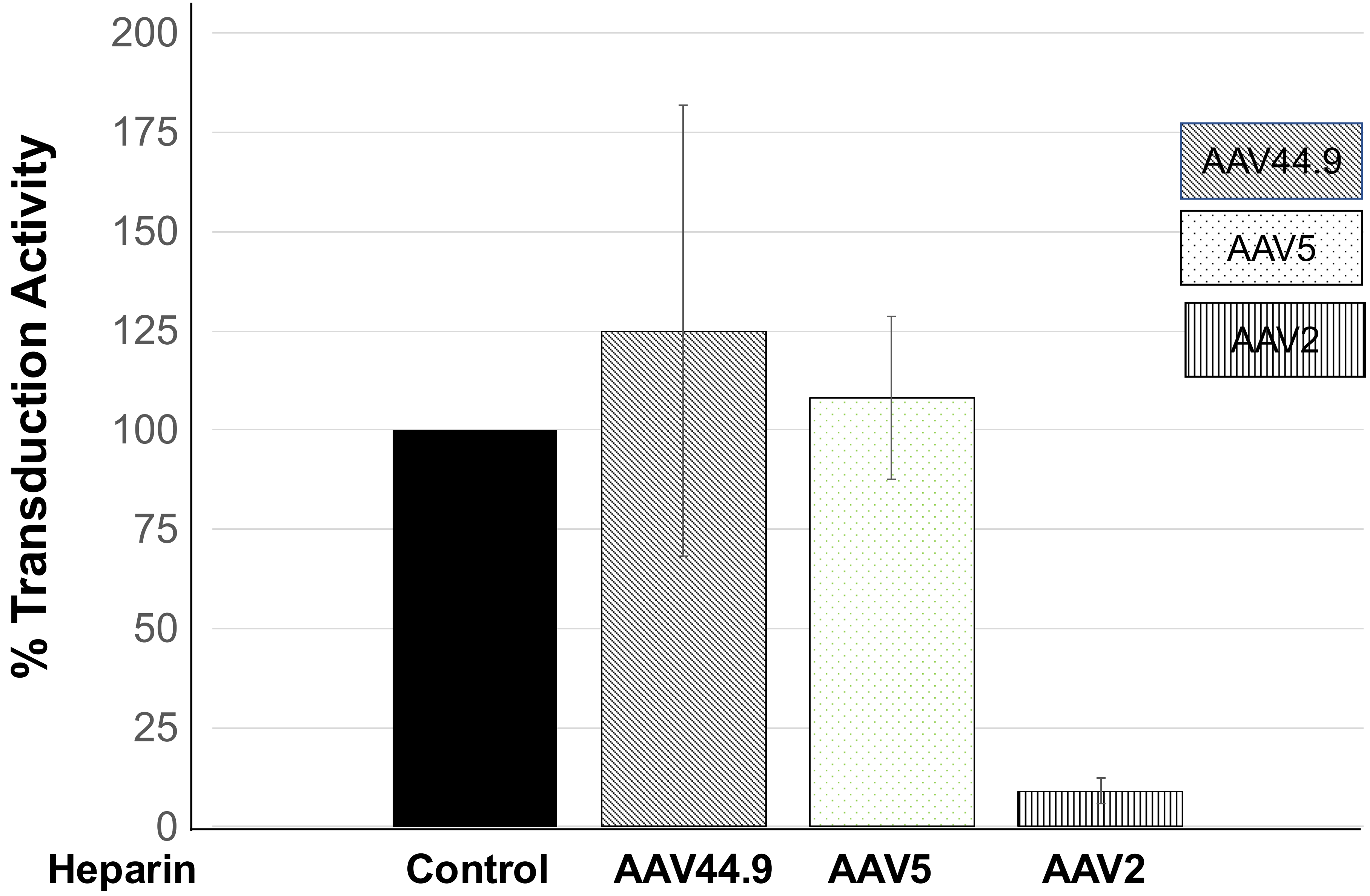

200
175
150
125
100
75
50
25
0
% Transduction Activity
AAV44.9
AAV5
AAV2
Heparin
Control
AAV44.9
AAV5
AAV2

Fig. 2 A

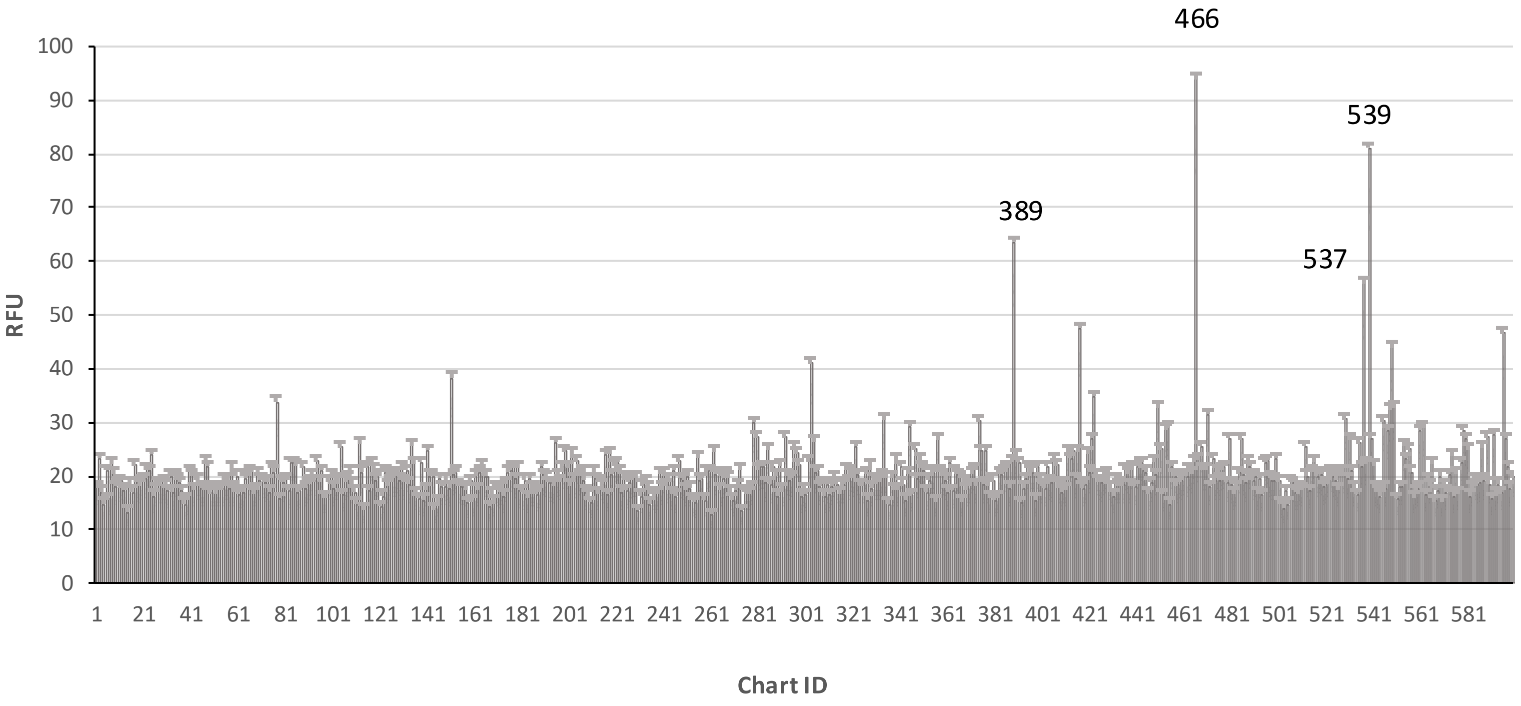

466	Glca1-4Glca1-4Glca1-4Glcb-Sp1
539	Galb1-4GlcNAcb1-3Galb1-4GlcNAcb1-3Galb1-4GlcNAcb1-2Mana1-6(Galb1-4GlcNAcb1-3Galb1-4GlcNAcb1-3Galb1-4GlcNAcb1-2Mana1-3)Manb1-4GlcNAcb1-4GlcNAcb-Sp12
389	GalNAca1-3(Fuca1-2)Galb1-3GalNAca1-3(Fuca1-2)Galb1-4GlcNAcb-Sp0
537	GlcNAcb1-3Galb1-4GlcNAcb1-3Galb1-4GlcNAcb1-2Mana1-6(GlcNAcb1-3Galb1-4GlcNAcb1-3Galb1-4GlcNAcb1-2Mana1-3)Manb1-4GlcNAcb1-4GlcNAcb-Sp12

Fig. 2B

Glycan 466

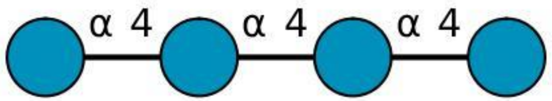

Glc(a4)Glc(a4)Glc(a4)Glc

Glycan 539

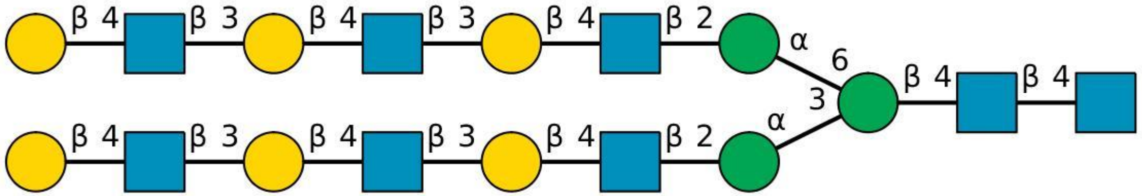

Gal(b4)GlcNAc(b3)Gal(b4)GlcNAc(b3)Gal(b4)GlcNAc(b2)Man(a6)[Gal(b4)GlcNAc(b3)Gal(b4)GlcNAc(b3)Gal(b4)GlcNAc(b2)Man(a3)]Man(b4)GlcNAc(b4)GlcNAc

Glycan 389

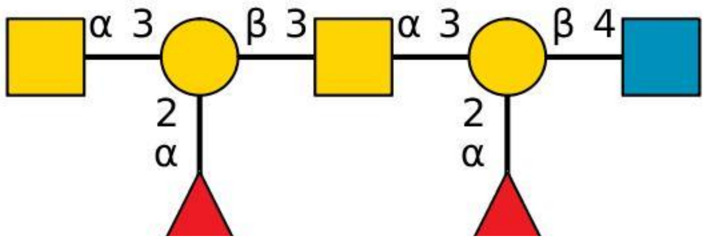

GalNAc(a3)[Fuc(a2)]Gal(b3)GalNAc(a3)[Fuc(a2)]Gal(b4)GlcNAc

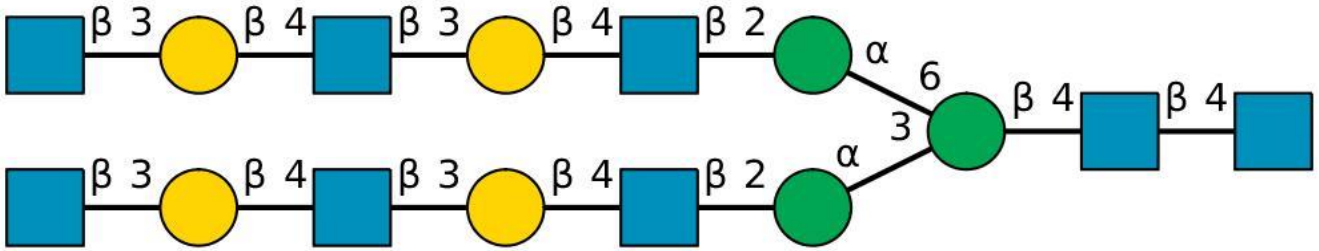

GlcNAc(b3)Gal(b4)GlcNAc(b3)Gal(b4)GlcNAc(b2)Man(a6)[GlcNAc(b3)Gal(b4)GlcNAc(b3)Gal(b4)GlcNAc(b2)Man(a3)]Man(b4)GlcNAc(b4)GlcNAc

Glycan 537

Fig. 3

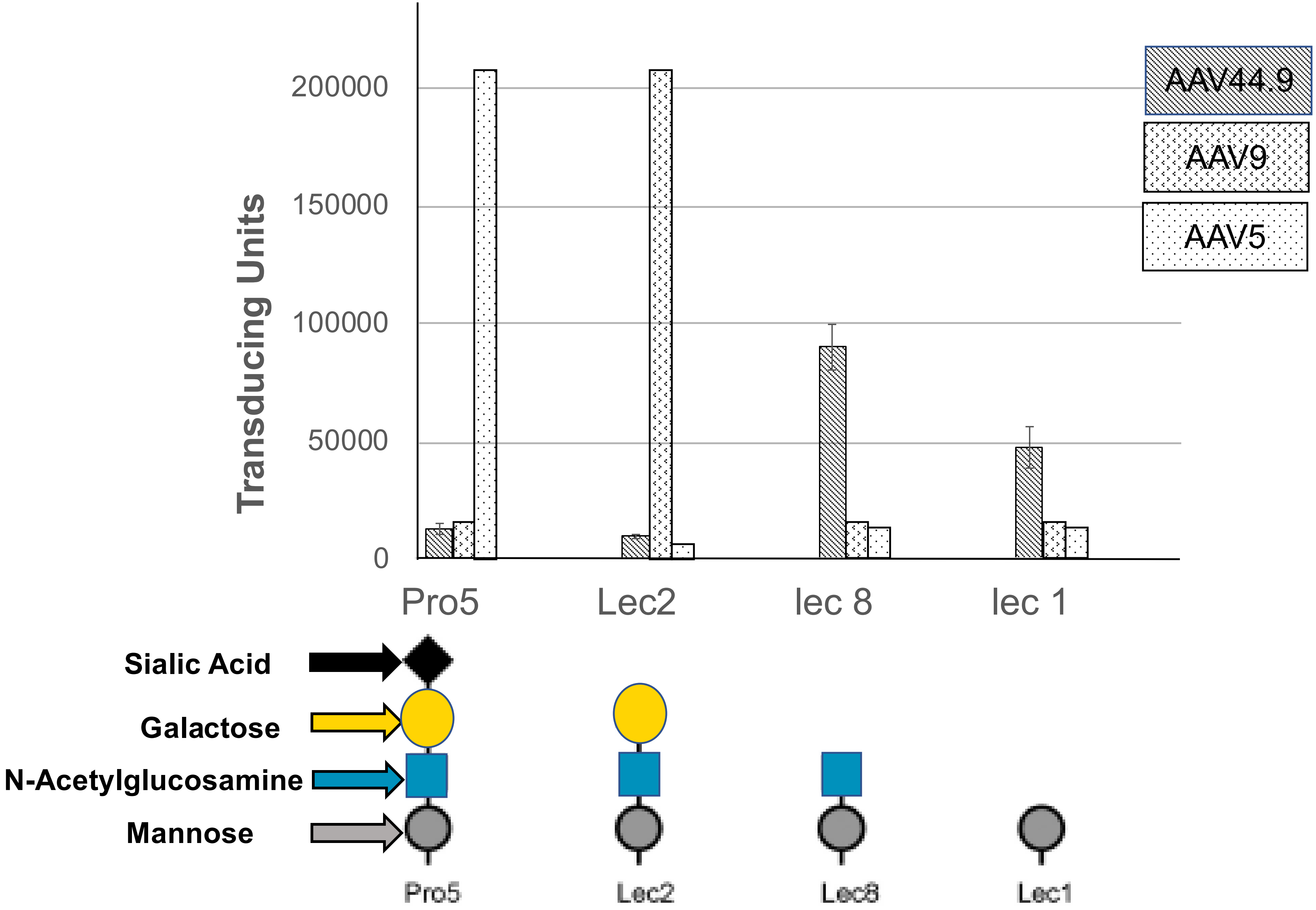

Transducing Units
200000
150000
100000
50000
0
AAV44.9
AAV9
AAV5
Pro5
Lec2
lec 8
lec 1
Sialic Acid
Galactose
N-Acetylglucosamine
Mannose
Pro5
Lec2
Lec8
Lec1

Fig. 4A

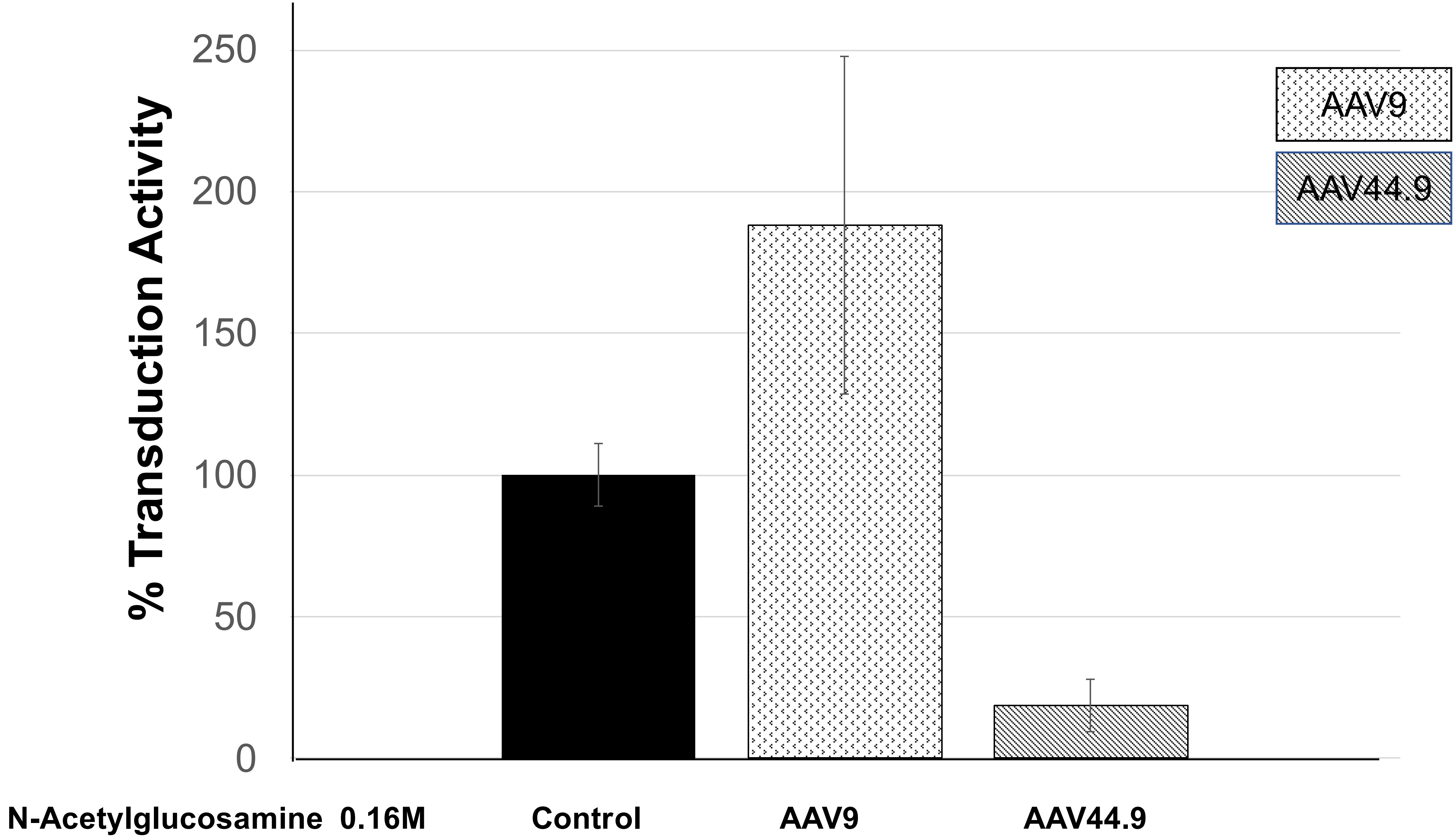
250
200
150
100
50
0
% Transduction Activity
AAV9
AAV44.9
N-Acetylglucosamine 0.16M
Control
AAV9
AAV44.9

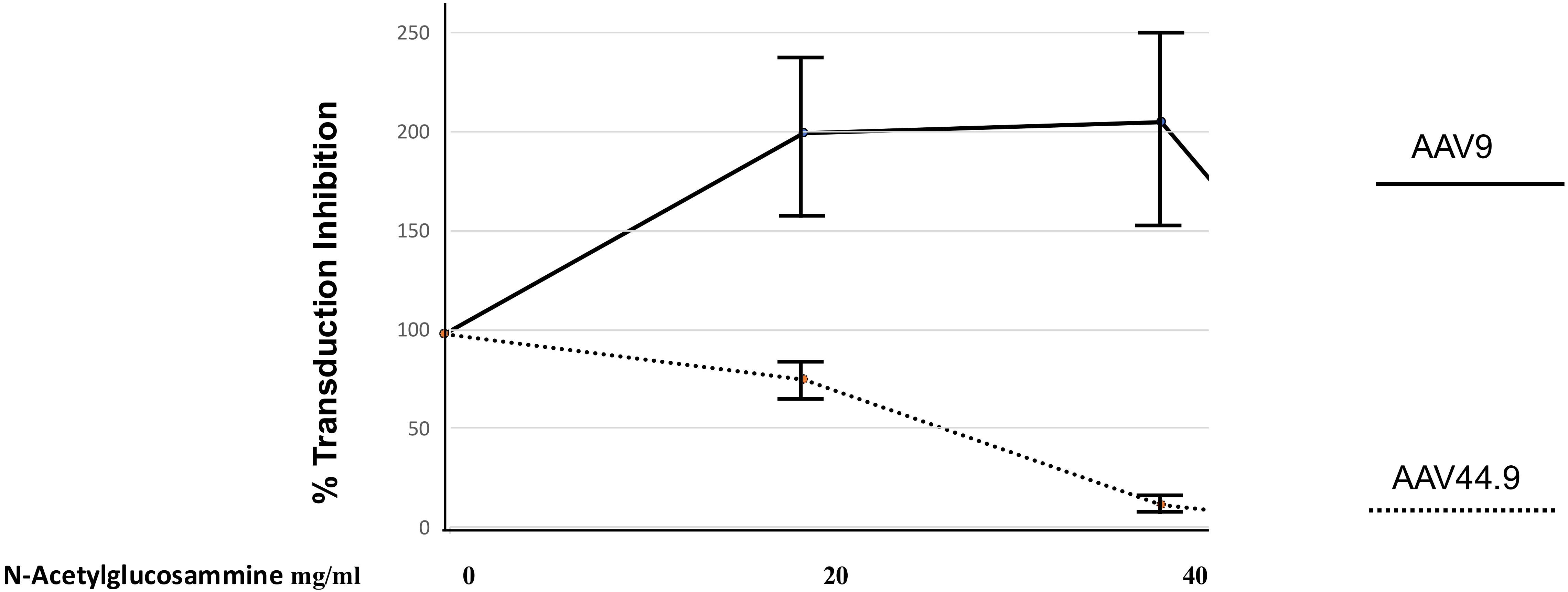
% Transduction Inhibition
250
200
150
100
50
0
N-Acetylglucosammine mg/ml
0
20
40
AAV9
AAV44.9

Fig. 4B

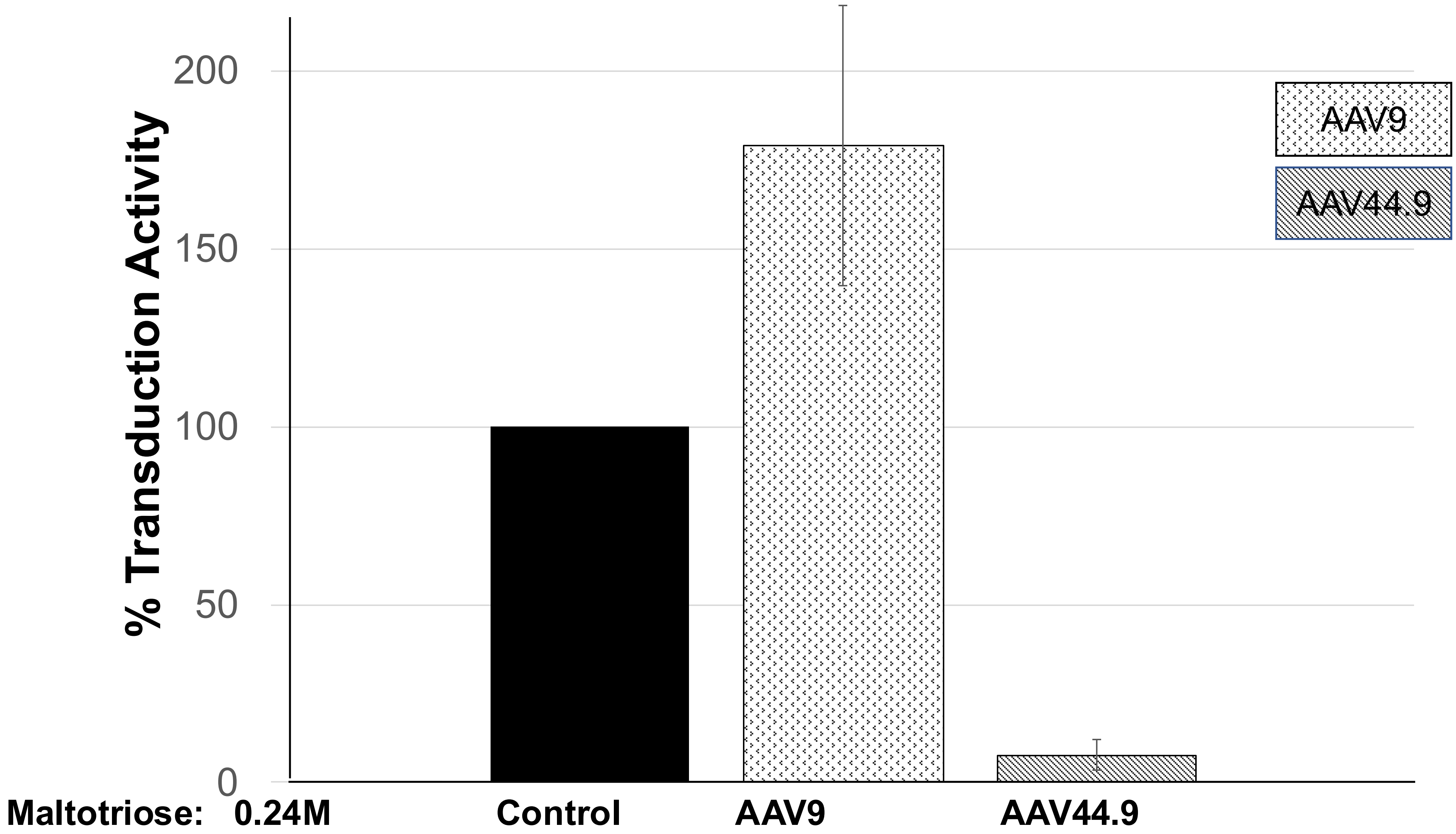
% Transduction Activity
200
150
100
50
0
AAV9
AAV44.9
Maltotriose: 0.24M
Control
AAV9
AAV44.9

Fig. 4C

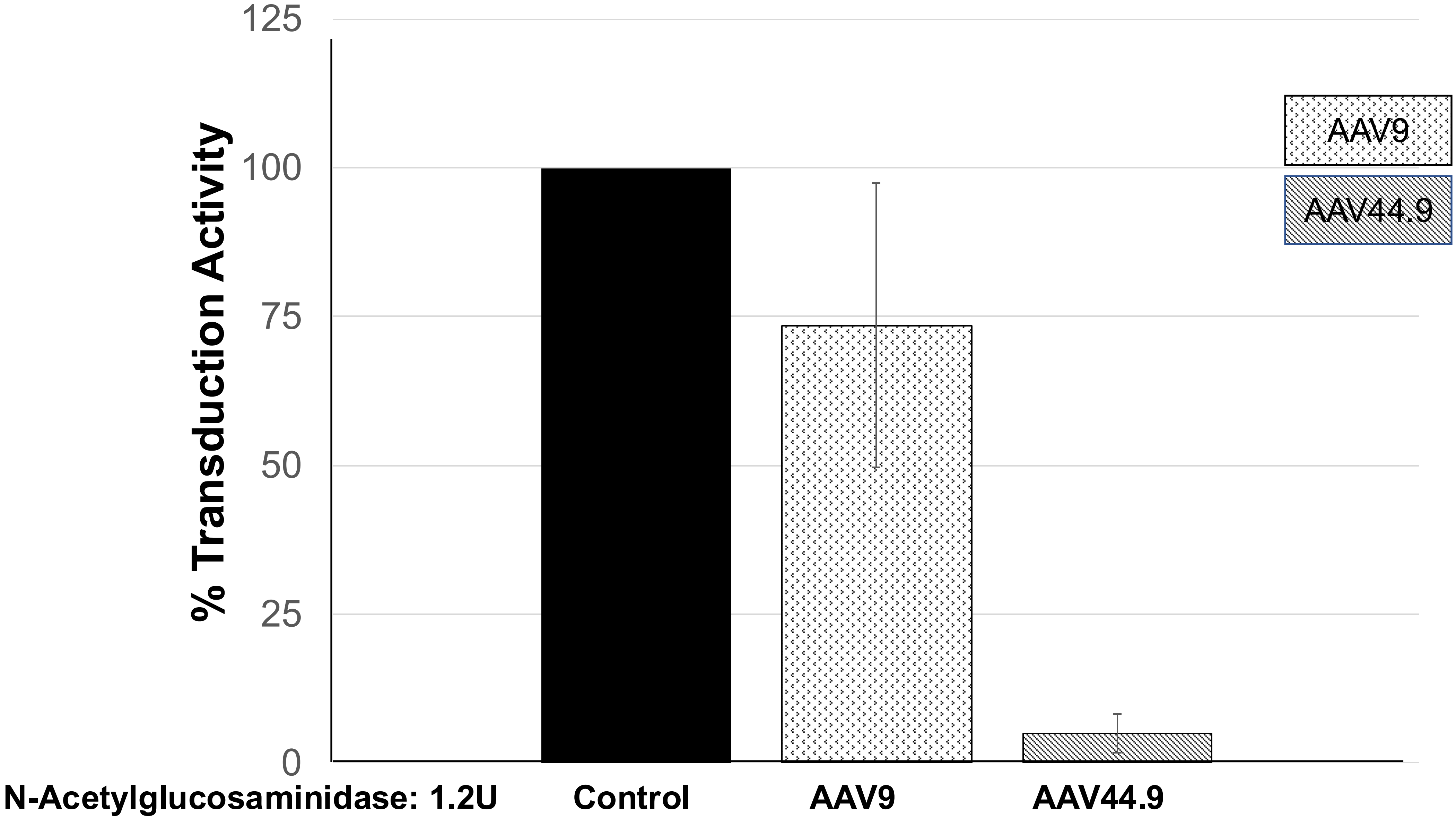
125
100
75
50
25
0
% Transduction Activity
AAV9
AAV44.9
N-Acetylglucosaminidase: 1.2U
Control
AAV9
AAV44.9

Fig. 4D

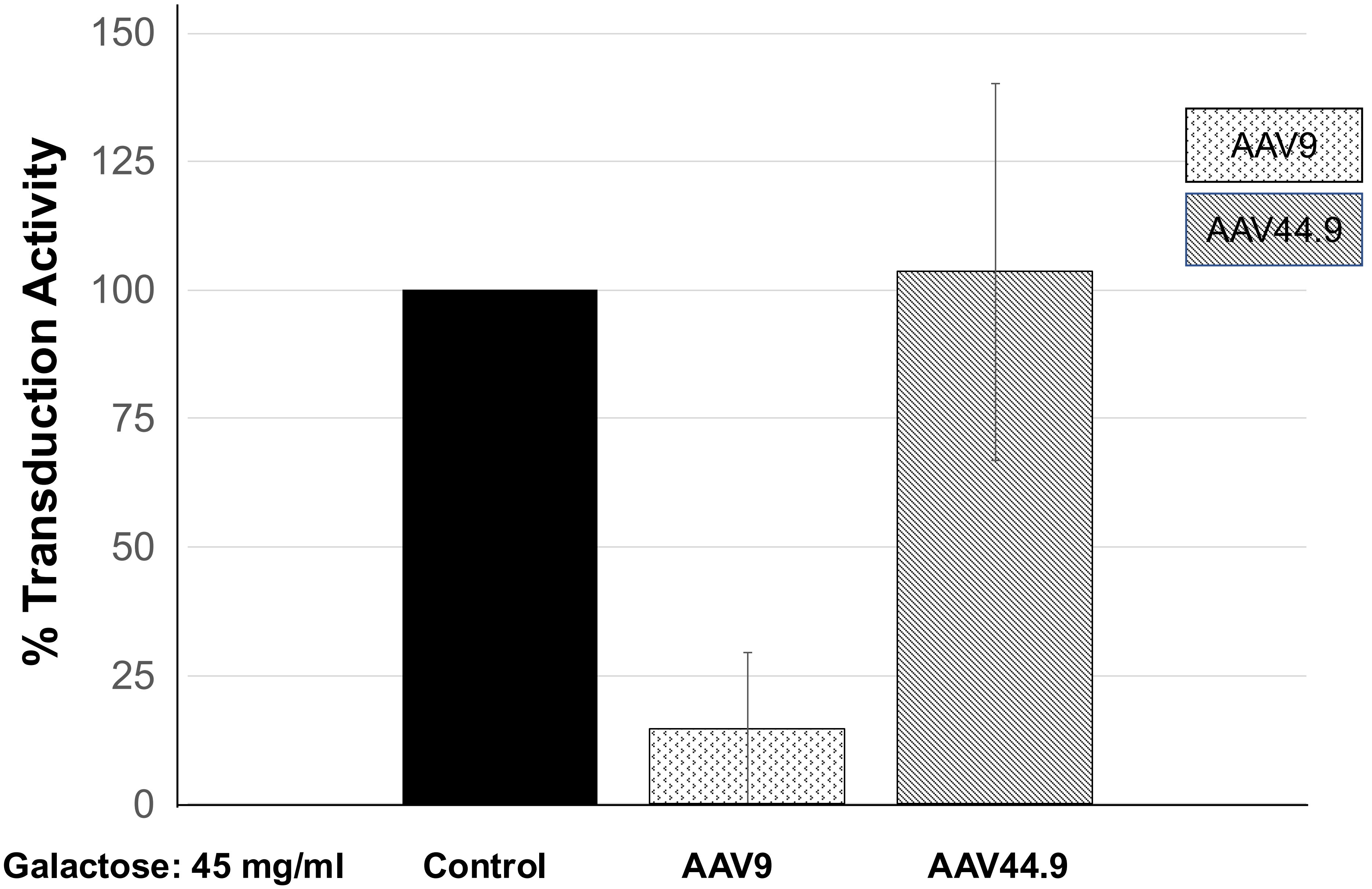

% Transduction Activity
150
125
100
75
50
25
0
AAV9
AAV44.9
Galactose: 45 mg/ml
Control
AAV9
AAV44.9

Fig. 5A

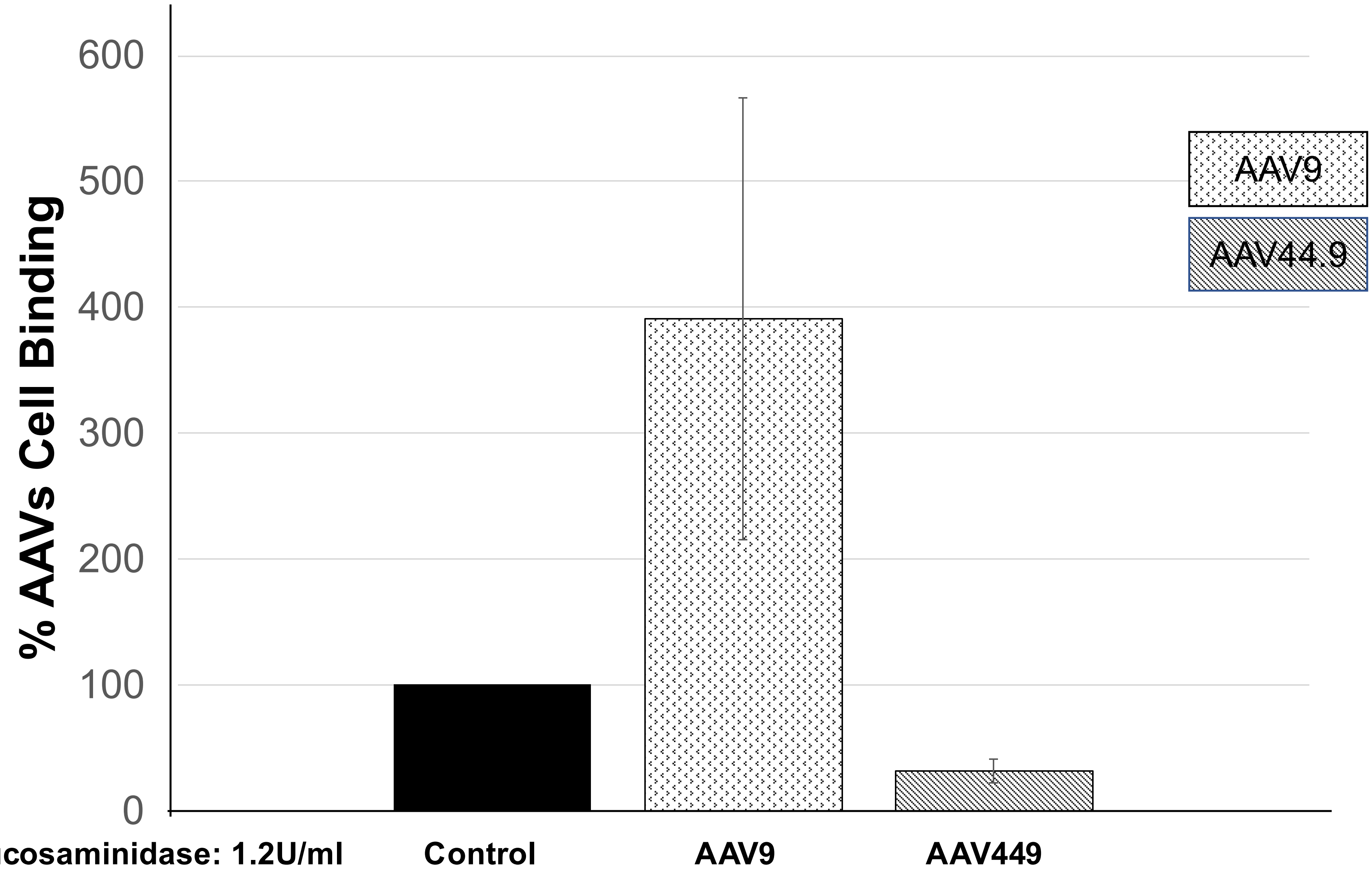

% AAVs Cell Binding
600
500
400
300
200
100
0
AAV9
AAV44.9
N-acetylglucosaminidase: 1.2U/ml
Control
AAV9
AAV449

Table 1

AAV44.9	AA	AAVRh8
Thr (T)	179	Ser (S)
Ser (S)	473	Asn (N)
Ser (S)	483	Cys (C)
Glu (E)	531	Asp (D)

Fig. 6A

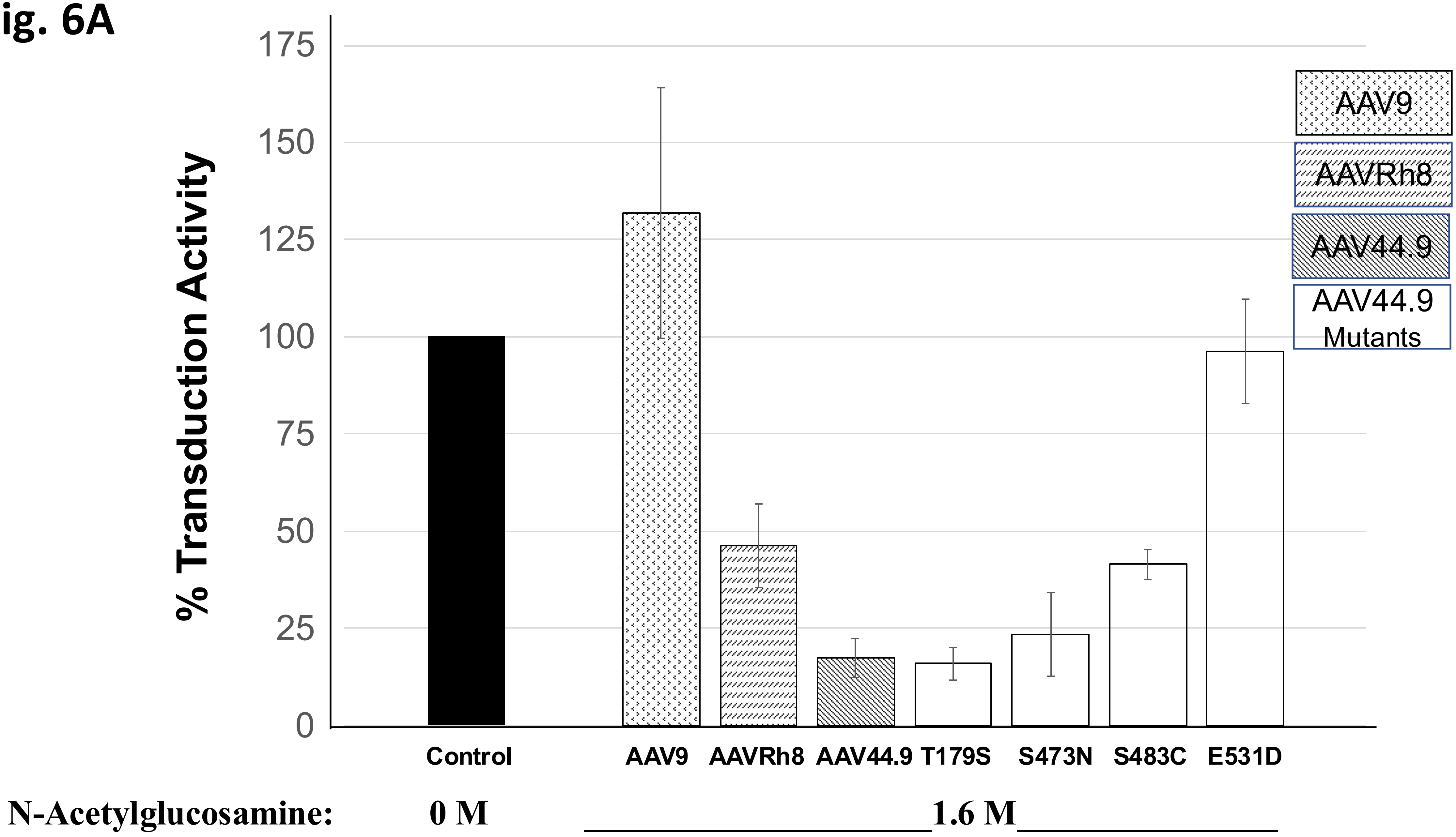
% Transduction Activity
175
150
125
100
75
50
25
0
AAV9
AAVRh8
AAV44.9
AAV44.9 Mutants
Control
AAV9
AAVRh8
AAV44.9
T179S
S473N
S483C
E531D
N-Acetylglucosamine:
0 M
1.6 M

Fig. 6B

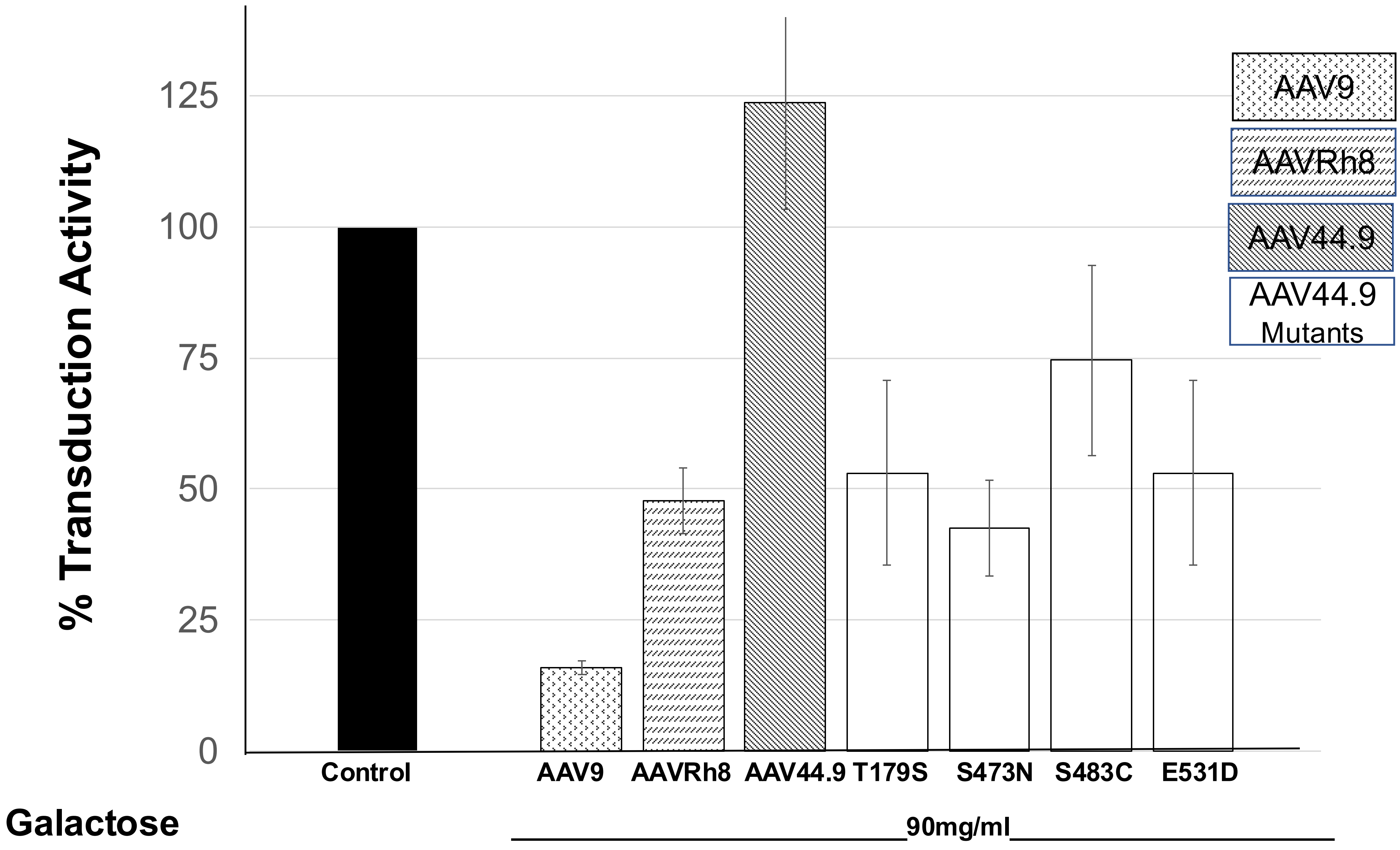
% Transduction Activity
125
100
75
50
25
0
AAV9
AAVRh8
AAV44.9
AAV44.9 Mutants
Control
AAV9
AAVRh8
AAV44.9
T179S
S473N
S483C
E531D
Galactose
90mg/ml

Fig. 7

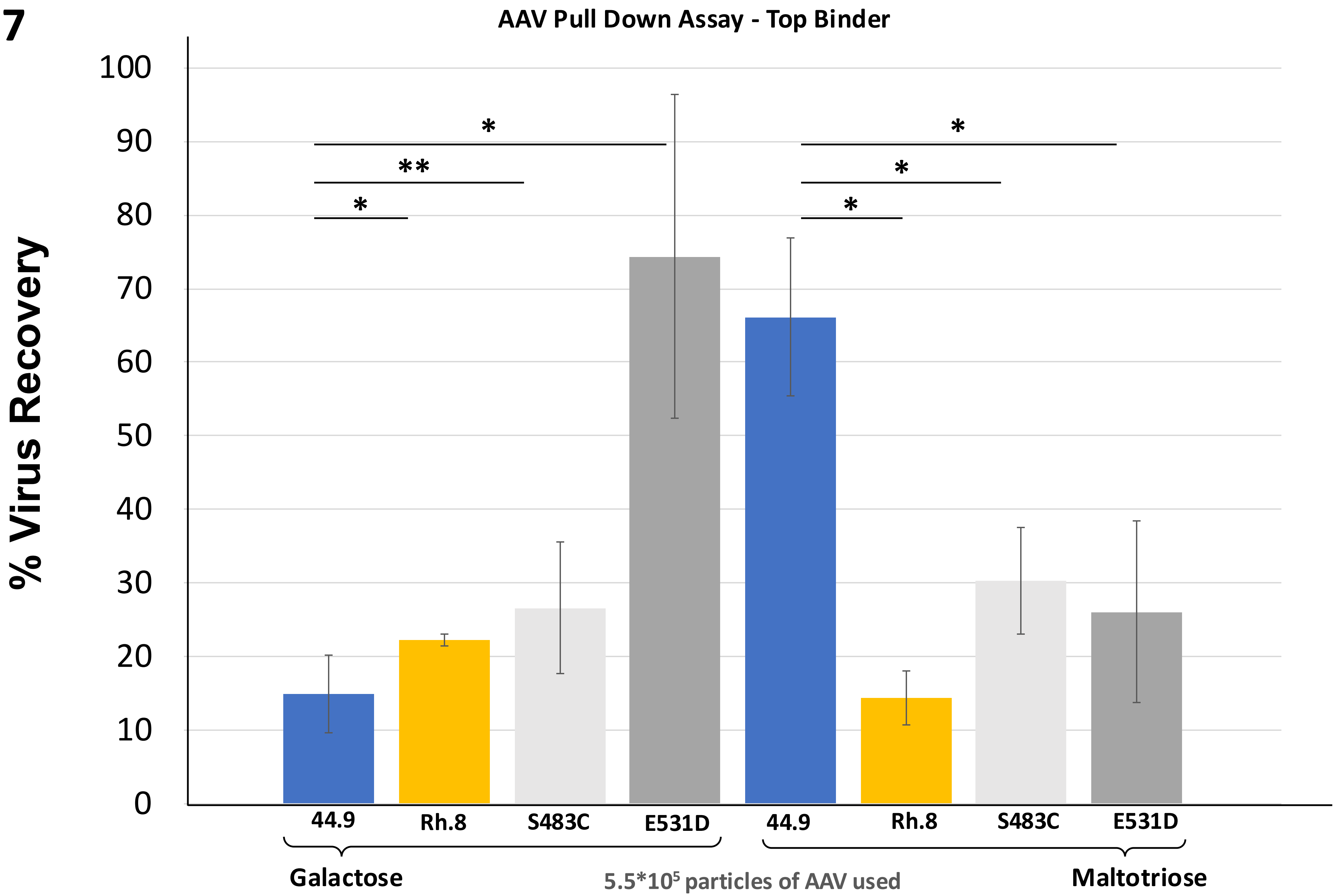

AAV Pull Down Assay - Top Binder
% Virus Recovery
100
90
80
70
60
50
40
30
20
10
0
*
**
*
*
*
*
44.9
Rh.8
S483C
E531D
44.9
Rh.8
S483C
E531D
Galactose
5.5*10^5 particles of AAV used
Maltotriose
