# Supplementary material for: Terminal glucose as a receptor for adeno-associated virus 44.9
Source: J Virol. 2026 Mar 23;100(4):e00254-25. doi: 10.1128/jvi.00254-25 (PMC13098211; doi:10.1128/jvi.00254-25)
Supplement: Fig. S2 — Amino acid sequence alignment of AAV44.9 and AAVrh.8R capsid proteins. [file jvi.00254-25-s0002.pdf]

Fig. 1S

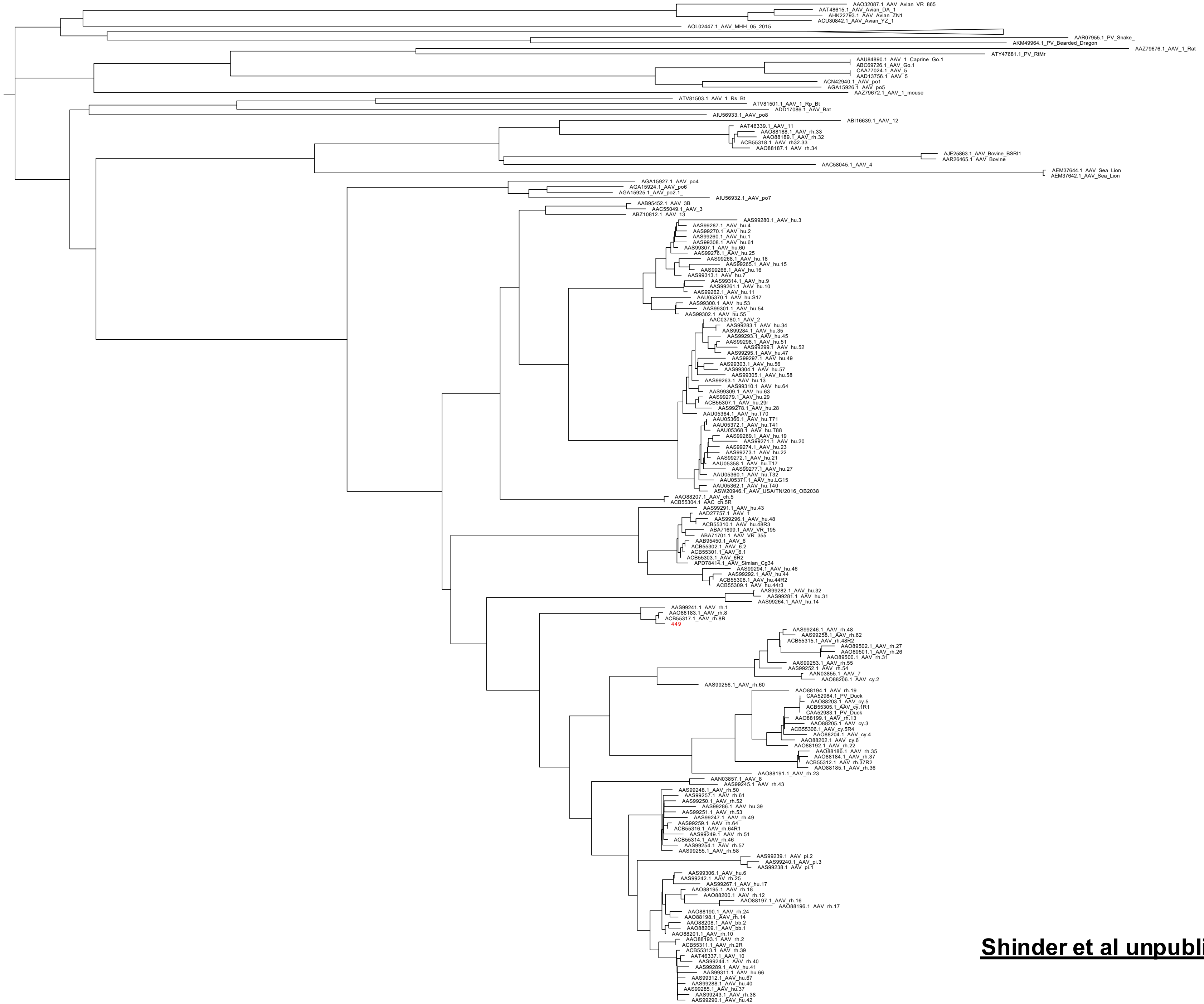

Shinder et al unpublished

```
AAV44.9 1    MAADGYLPDWLEDNLSEGIREWWDLKPGAPKPKANQQKQDDGRGLVLPGYKYLGPFNGLD  60
             MAADGYLPDWLEDNLSEGIREWWDLKPGAPKPKANQQKQDDGRGLVLPGYKYLGPFNGLD
AAVRh8   1   MAADGYLPDWLEDNLSEGIREWWDLKPGAPKPKANQQKQDDGRGLVLPGYKYLGPFNGLD  60

AAV44.1 61   KGEPVNAADAAALEHDKAYDQQLKAGDNPYLRYNHADAEFQERLQEDTSFGGNLGRAVFQ  120
             KGEPVNAADAAALEHDKAYDQQLKAGDNPYLRYNHADAEFQERLQEDTSFGGNLGRAVFQ
AAVRh8   61  KGEPVNAADAAALEHDKAYDQQLKAGDNPYLRYNHADAEFQERLQEDTSFGGNLGRAVFQ  120

AAV44.1 121  AKKRVLEPLGLVEEGAKTAPGKKRPVEQSPQEPDSSSGIGKTGQQPAKKRLNFGQTGDTE  180
             AKKRVLEPLGLVEEGAKTAPGKKRPVEQSPQEPDSSSGIGKTGQQPAKKRLNFGQTGD+E
AAVRh8   121 AKKRVLEPLGLVEEGAKTAPGKKRPVEQSPQEPDSSSGIGKTGQQPAKKRLNFGQTGDSE  180

AAV44.1 181  SVPDPQPLGEPPAAPSGLGPNTMASGGGAPMADNNEGADGVGNSSGNWHCDSTWLGDRVI  240
             SVPDPQPLGEPPAAPSGLGPNTMASGGGAPMADNNEGADGVGNSSGNWHCDSTWLGDRVI
AAVRh8   181 SVPDPQPLGEPPAAPSGLGPNTMASGGGAPMADNNEGADGVGNSSGNWHCDSTWLGDRVI  240

AAV44.1 241  TTSTRTWALPTYNNHLYKQISNGTSGGSTNDNTYFGYSTPWGYFDFNRFHCHFSPRDWQR  300
             TTSTRTWALPTYNNHLYKQISNGTSGGSTNDNTYFGYSTPWGYFDFNRFHCHFSPRDWQR
AAVRh8   241 TTSTRTWALPTYNNHLYKQISNGTSGGSTNDNTYFGYSTPWGYFDFNRFHCHFSPRDWQR  300

AAV44.1 301  LINNNWGFRPKRLNFKLFNIQVKEVTTNEGTKTIANNLTSTVQVFTDSEYQLPYVLGSAH  360
             LINNNWGFRPKRLNFKLFNIQVKEVTTNEGTKTIANNLTSTVQVFTDSEYQLPYVLGSAH
AAVRh8   301 LINNNWGFRPKRLNFKLFNIQVKEVTTNEGTKTIANNLTSTVQVFTDSEYQLPYVLGSAH  360

AAV44.1 361  QGCLPPFPADVFMVPQYGYLTLNNGSQALGRSSFYCLEYFPSQMLRTGNNFQFSYTFEDV  420
             QGCLPPFPADVFMVPQYGYLTLNNGSQALGRSSFYCLEYFPSQMLRTGNNFQFSYTFEDV
AAVRh8   361 QGCLPPFPADVFMVPQYGYLTLNNGSQALGRSSFYCLEYFPSQMLRTGNNFQFSYTFEDV  420
                                                   IV
AAV44.1 421  PFHSSYAHSQSLDRLMNPLIDQYLYYLVRTQTTGTGGTQTLAFSQAGPSSMASQARNWVP  480
             PFHSSYAHSQSLDRLMNPLIDQYLYYLVRTQTTGTGGTQTLAFSQAGPSSMA+QARNWVP
AAVRh8   421 PFHSSYAHSQSLDRLMNPLIDQYLYYLVRTQTTGTGGTQTLAFSQAGPSSMANQARNWVP  480
                             V
AAV44.1 481  GPSYRQQRVSTTTNQNNNSNFAWTGAAKFKLNGRDSLMNPGVAMASHKDDEDRFFPSSGV  540
             GP YRQQRVSTTTNQNNNSNFAWTGAAKFKLNGRDSLMNPGVAMASHKDD+DRFFPSSGV
AAVRh8   481 GPCYRQQRVSTTTNQNNNSNFAWTGAAKFKLNGRDSLMNPGVAMASHKDDDDRFFPSSGV  540

AAV44.1 541  LIFGKQGAGNDGVDYSQVLITDEEEIKATNPVATEEYGAVAINNQAANTQAQTGLVHNQG  600
             LIFGKQGAGNDGVDYSQVLITDEEEIKATNPVATEEYGAVAINNQAANTQAQTGLVHNQG
AAVRh8   541 LIFGKQGAGNDGVDYSQVLITDEEEIKATNPVATEEYGAVAINNQAANTQAQTGLVHNQG  600

AAV44.1 601  VIPGMVWQNRDVYLQGPIWAKIPHTDGNFHPSPLMGGFGLKHPPPQILIKNTPVPADPPL  660
             VIPGMVWQNRDVYLQGPIWAKIPHTDGNFHPSPLMGGFGLKHPPPQILIKNTPVPADPPL
AAVRh8   601 VIPGMVWQNRDVYLQGPIWAKIPHTDGNFHPSPLMGGFGLKHPPPQILIKNTPVPADPPL  660

AAV44.1 661  TFNQAKLNSFITQYSTGQVSVEIEWELQKENSKRWNPEIQYTSNYYKSTNVDFAVNTEGV  720
             TFNQAKLNSFITQYSTGQVSVEIEWELQKENSKRWNPEIQYTSNYYKSTNVDFAVNTEGV
AAVRh8   661 TFNQAKLNSFITQYSTGQVSVEIEWELQKENSKRWNPEIQYTSNYYKSTNVDFAVNTEGV  720

AAV44.1 721  YSEPRPIGTRYLTRNL  736
             YSEPRPIGTRYLTRNL
AAVRh8   721 YSEPRPIGTRYLTRNL  736
```

179 S→T

473 N→S

483 C→S

531 D→E

Fig. 1A

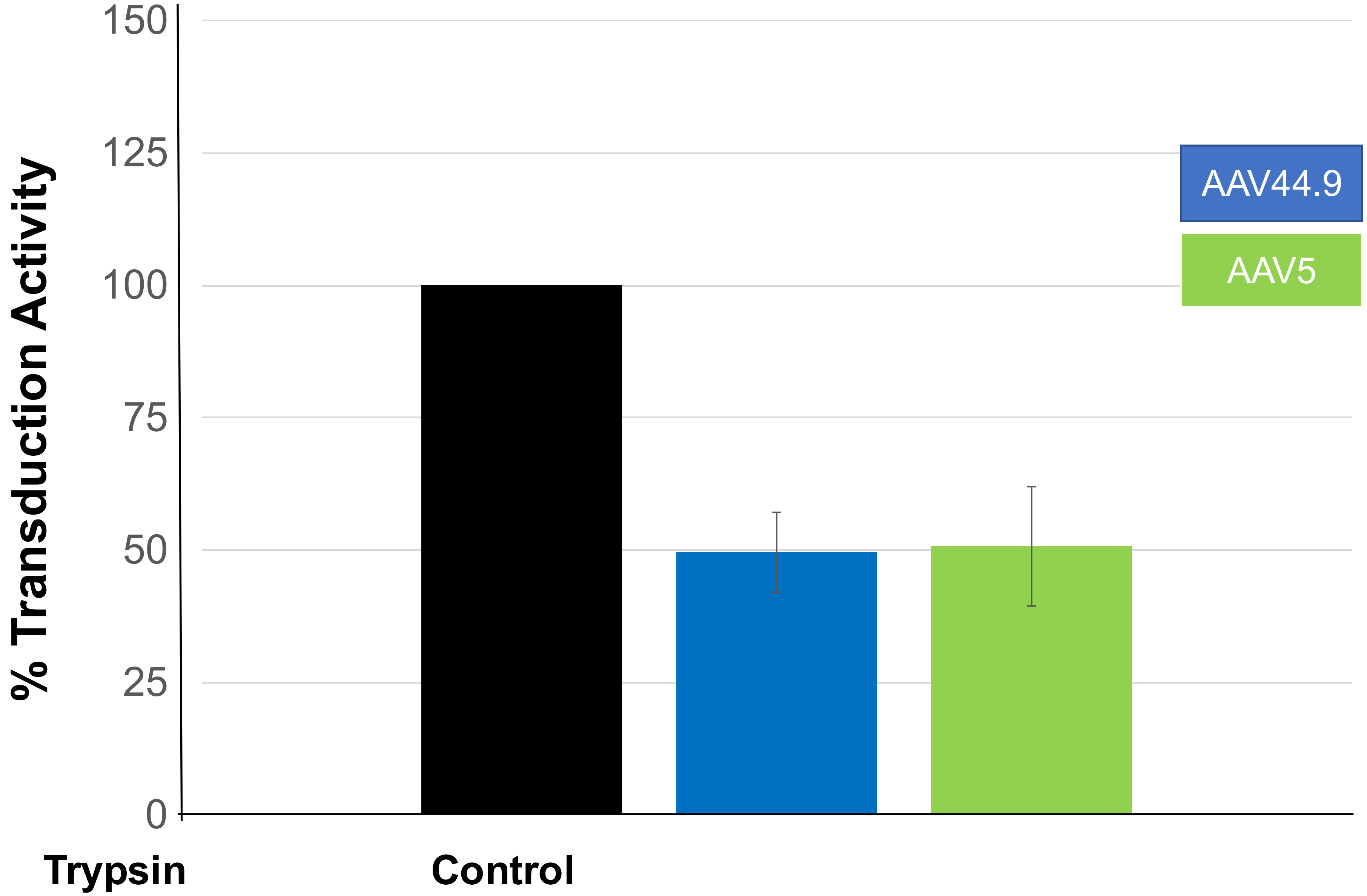

150
125
100
75
50
25
0
% Transduction Activity
AAV44.9
AAV5
Trypsin
Control

Fig. 1B

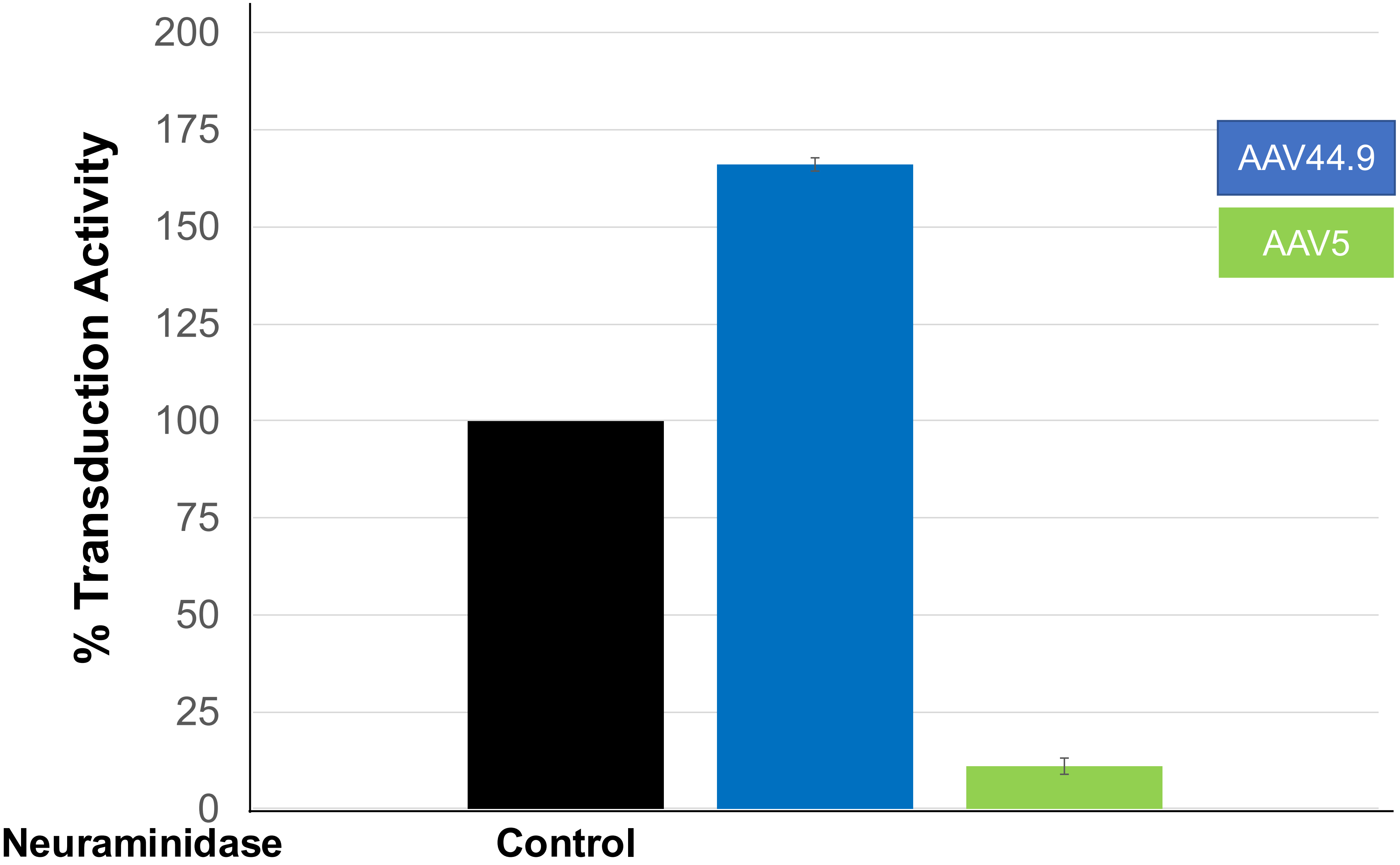

% Transduction Activity
200
175
150
125
100
75
50
25
0
AAV44.9
AAV5
Neuraminidase
Control

Fig. 1C

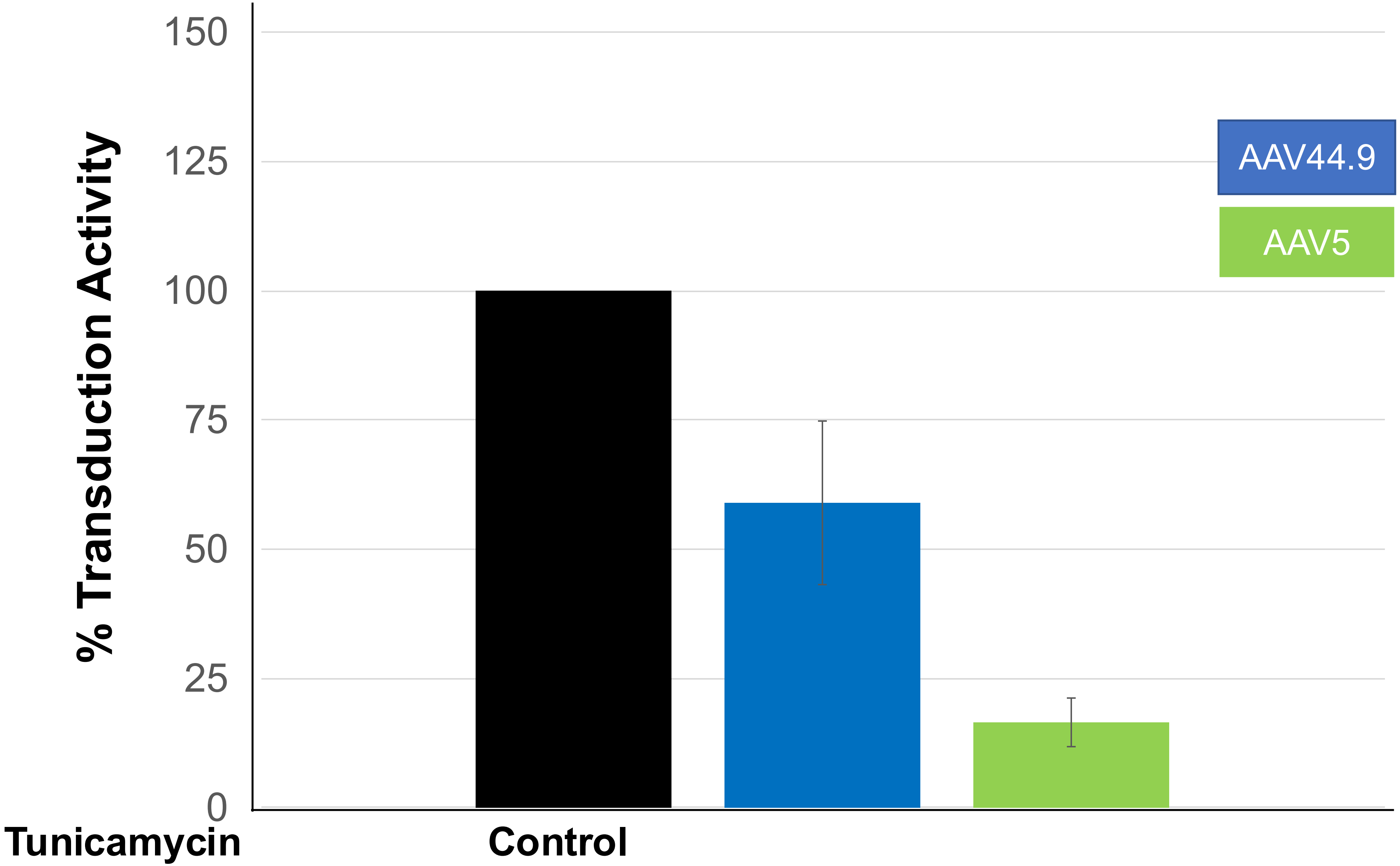
150
125
100
75
50
25
0
% Transduction Activity
AAV44.9
AAV5
Tunicamycin
Control

Fig. 1D

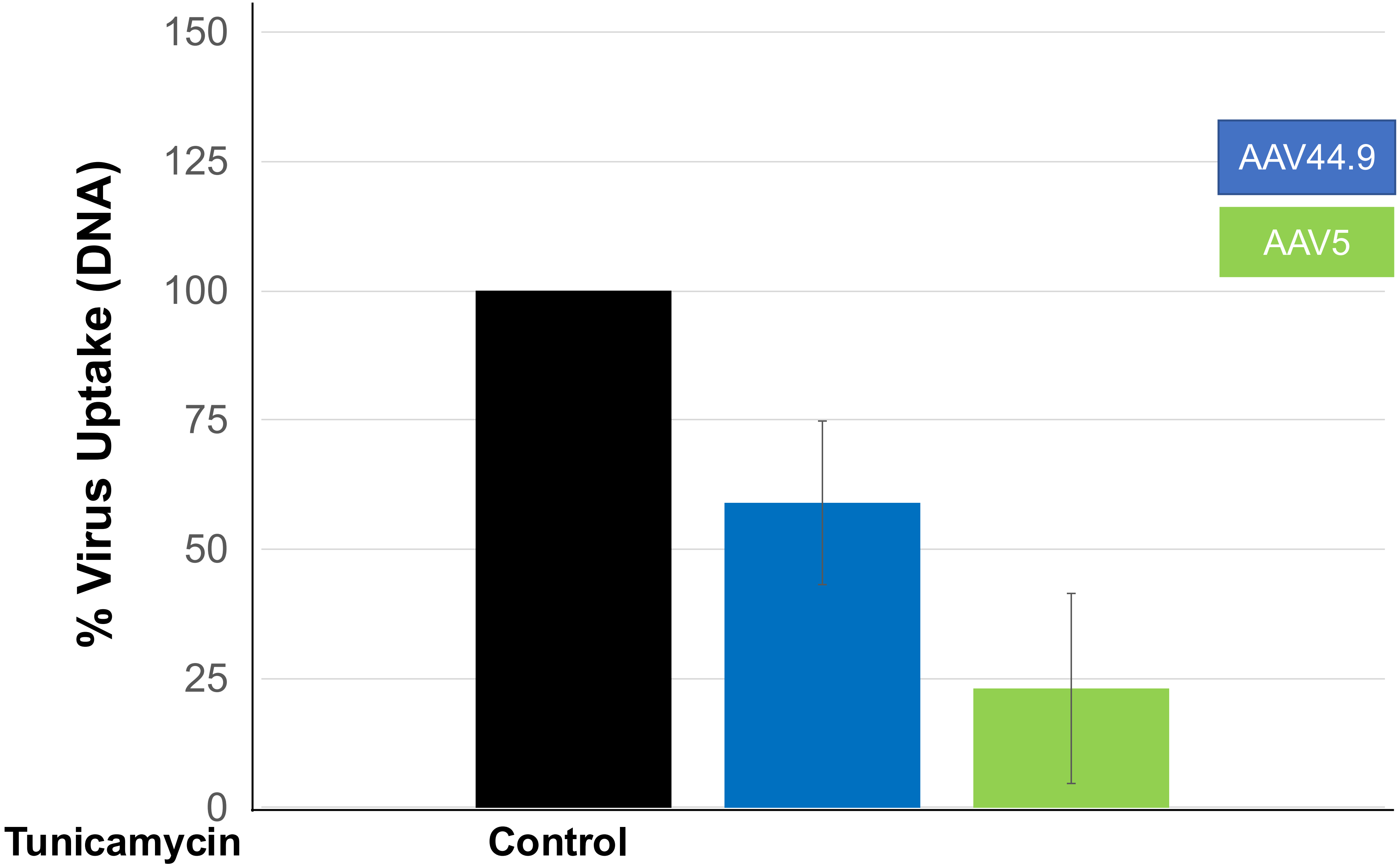

% Virus Uptake (DNA)
150
125
100
75
50
25
0
AAV44.9
AAV5
Tunicamycin
Control

Fig. 1E

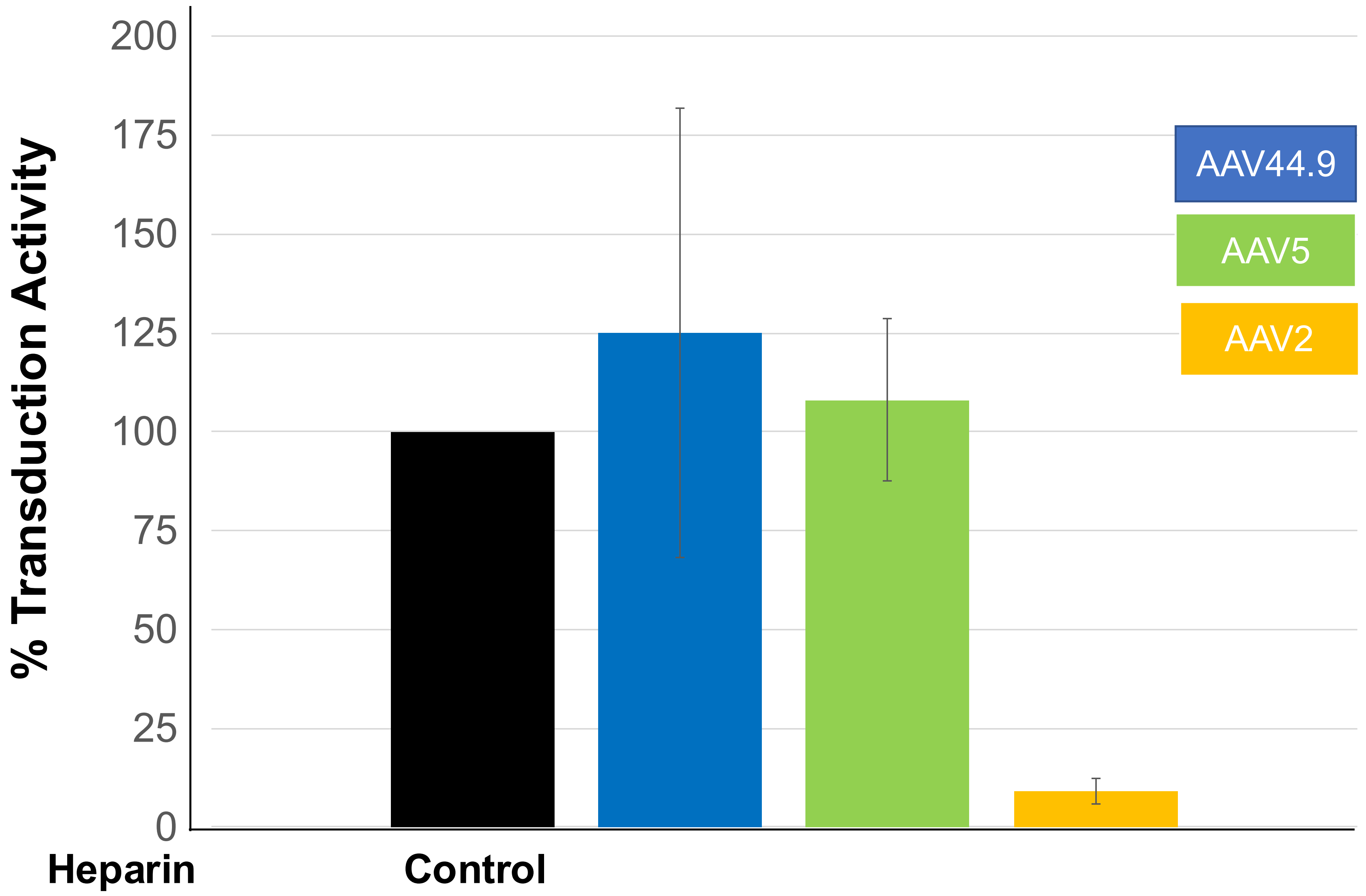
% Transduction Activity
200
175
150
125
100
75
50
25
0
AAV44.9
AAV5
AAV2
Heparin
Control

Fig. 2 A

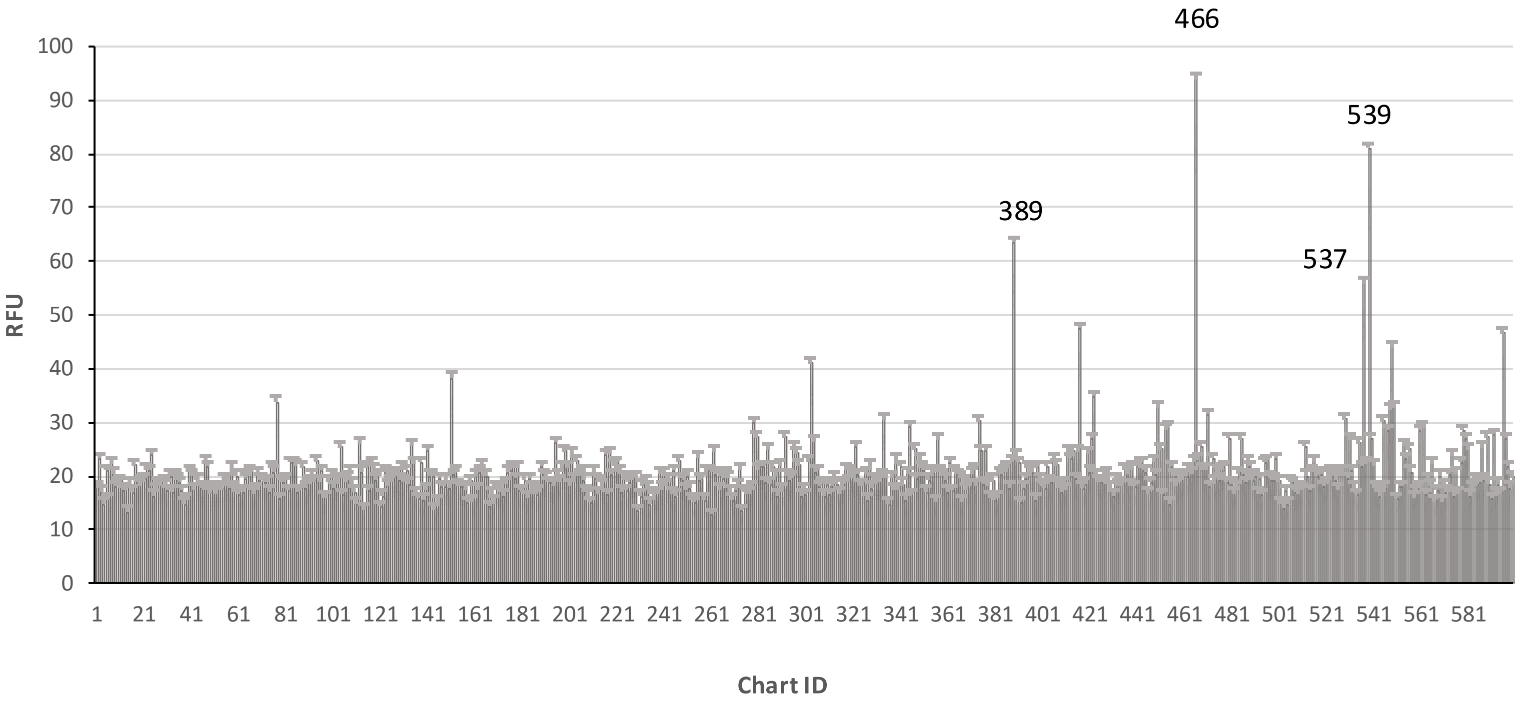

466	Glca1-4Glca1-4Glca1-4Glcb-Sp1
539	Galb1-4GlcNAcb1-3Galb1-4GlcNAcb1-3Galb1-4GlcNAcb1-2Mana1-6(Galb1-4GlcNAcb1-3Galb1-4GlcNAcb1-3Galb1-4GlcNAcb1-2Mana1-3)Manb1-4GlcNAcb1-4GlcNAcb-Sp12
389	GalNAca1-3(Fuca1-2)Galb1-3GalNAca1-3(Fuca1-2)Galb1-4GlcNAcb-Sp0
537	GlcNAcb1-3Galb1-4GlcNAcb1-3Galb1-4GlcNAcb1-2Mana1-6(GlcNAcb1-3Galb1-4GlcNAcb1-3Galb1-4GlcNAcb1-2Mana1-3)Manb1-4GlcNAcb1-4GlcNAcb-Sp12

Fig. 2B

Glycan 466

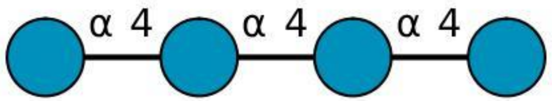

Glc(a4)Glc(a4)Glc(a4)Glc

Glycan 539

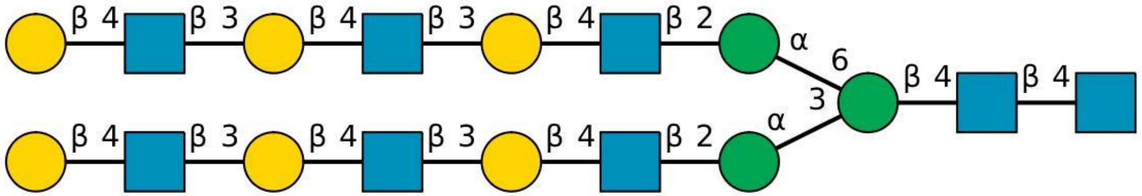

Gal(b4)GlcNAc(b3)Gal(b4)GlcNAc(b3)Gal(b4)GlcNAc(b2)Man(a6)[Gal(b4)GlcNAc(b3)Gal(b4)GlcNAc(b3)Gal(b4)GlcNAc(b2)Man(a3)]Man(b4)GlcNAc(b4)GlcNAc

Glycan 389

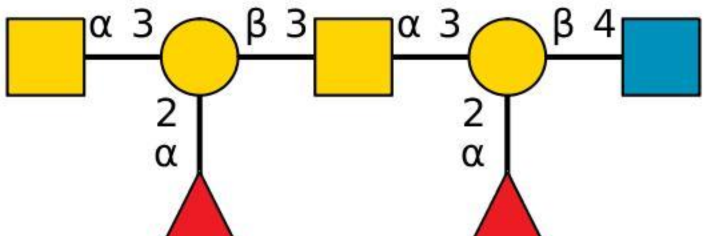

GalNAc(a3)[Fuc(a2)]Gal(b3)GalNAc(a3)[Fuc(a2)]Gal(b4)GlcNAc

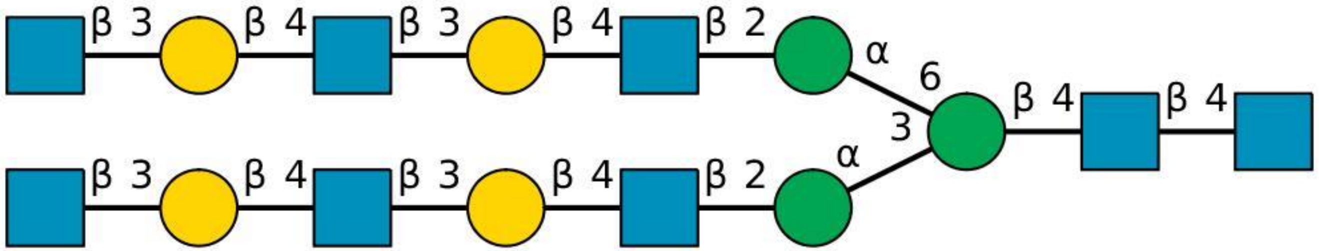

Glycan 537

GlcNAc(b3)Gal(b4)GlcNAc(b3)Gal(b4)GlcNAc(b2)Man(a6)[GlcNAc(b3)Gal(b4)GlcNAc(b3)Gal(b4)GlcNAc(b2)Man(a3)]Man(b4)GlcNAc(b4)GlcNAc

Fig. 3

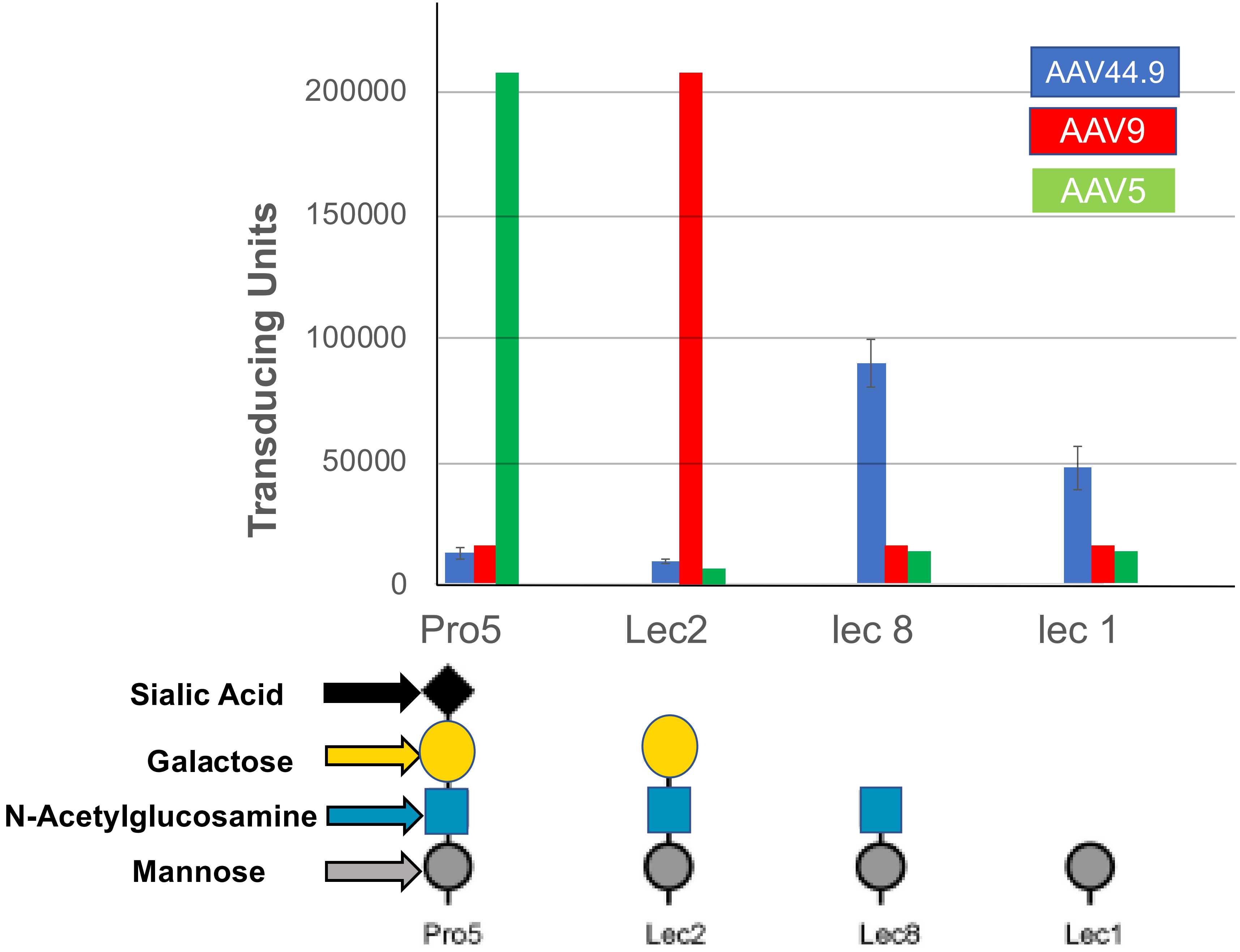
AAV44.9
AAV9
AAV5
Transducing Units
200000
150000
100000
50000
0
Pro5
Lec2
lec 8
lec 1
Sialic Acid
Galactose
N-Acetylglucosamine
Mannose
Pro5
Lec2
Lec8
Lec1

Fig. 4A

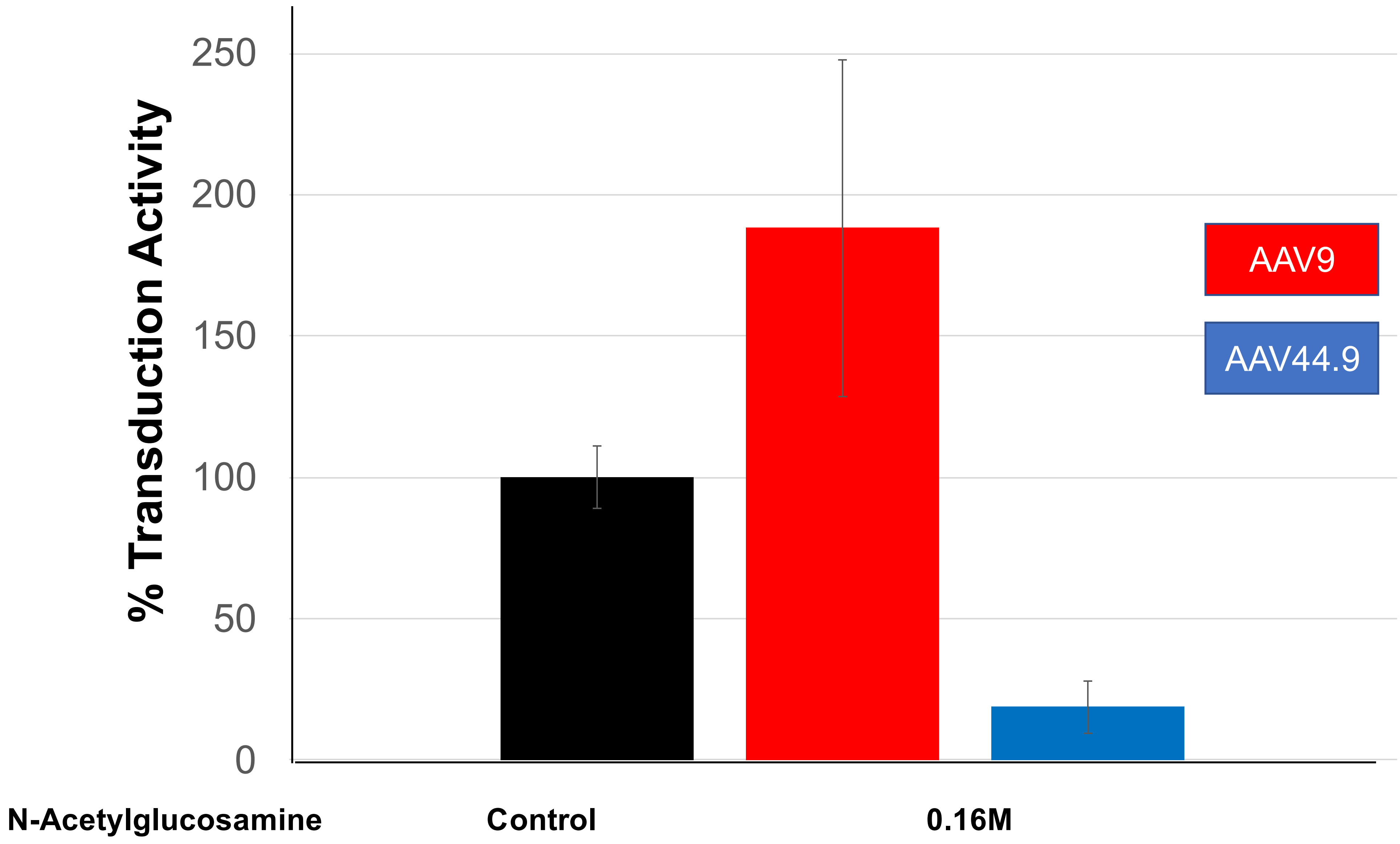
% Transduction Activity
250
200
150
100
50
0
AAV9
AAV44.9
N-Acetylglucosamine
Control
0.16M

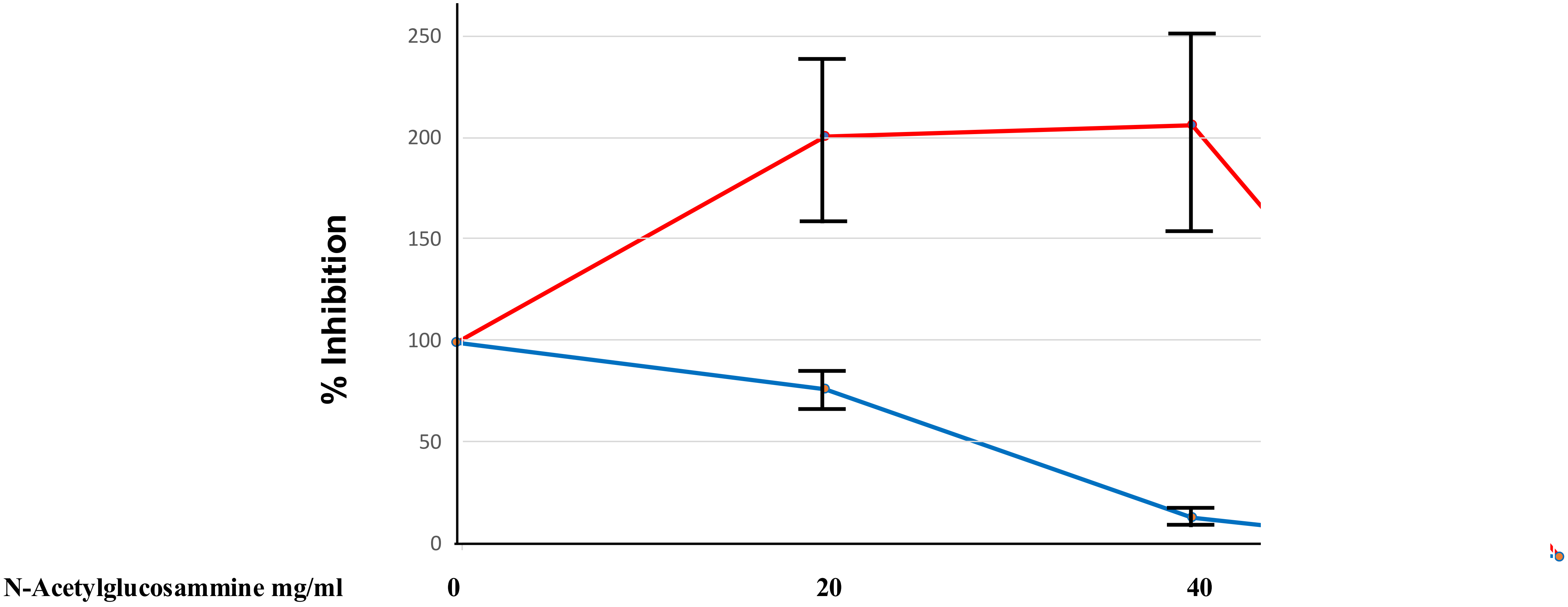

% Inhibition
250
200
150
100
50
0
N-Acetylglucosammine mg/ml
0
20
40

Fig. 4B

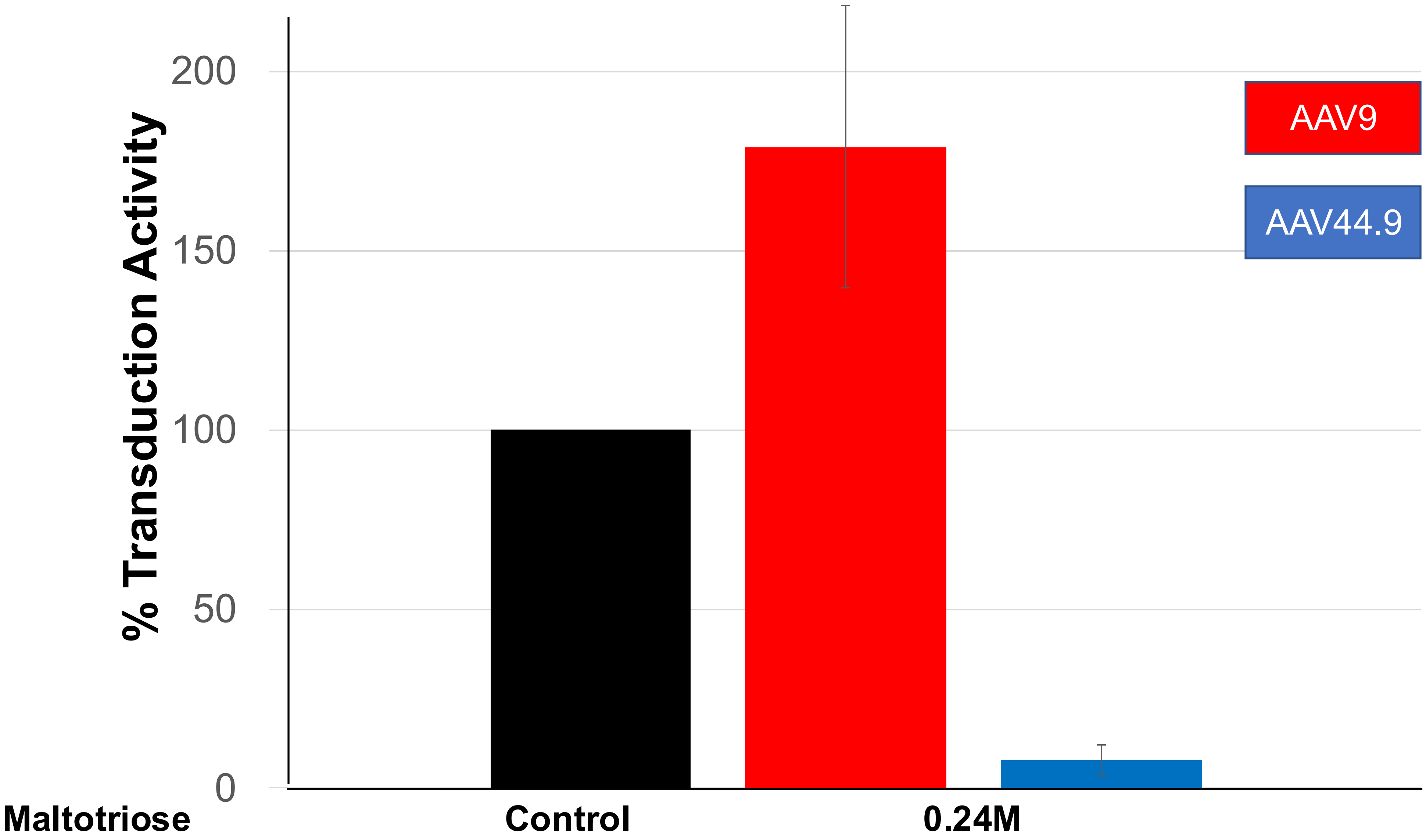
% Transduction Activity
200
150
100
50
0
AAV9
AAV44.9
Maltotriose
Control
0.24M

Fig. 4C

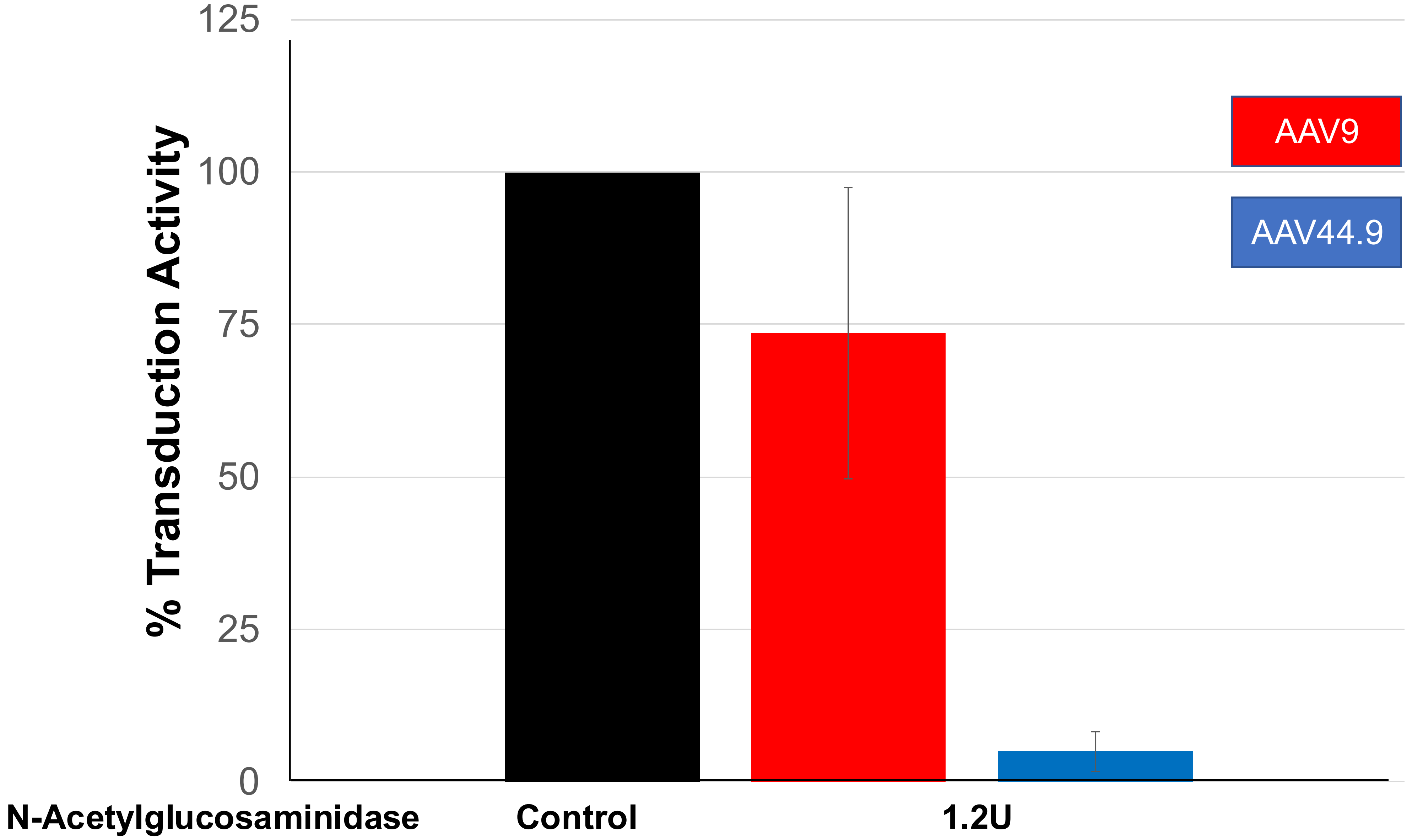
125
100
75
50
25
0
% Transduction Activity
AAV9
AAV44.9
N-Acetylglucosaminidase
Control
1.2U

Fig. 4D

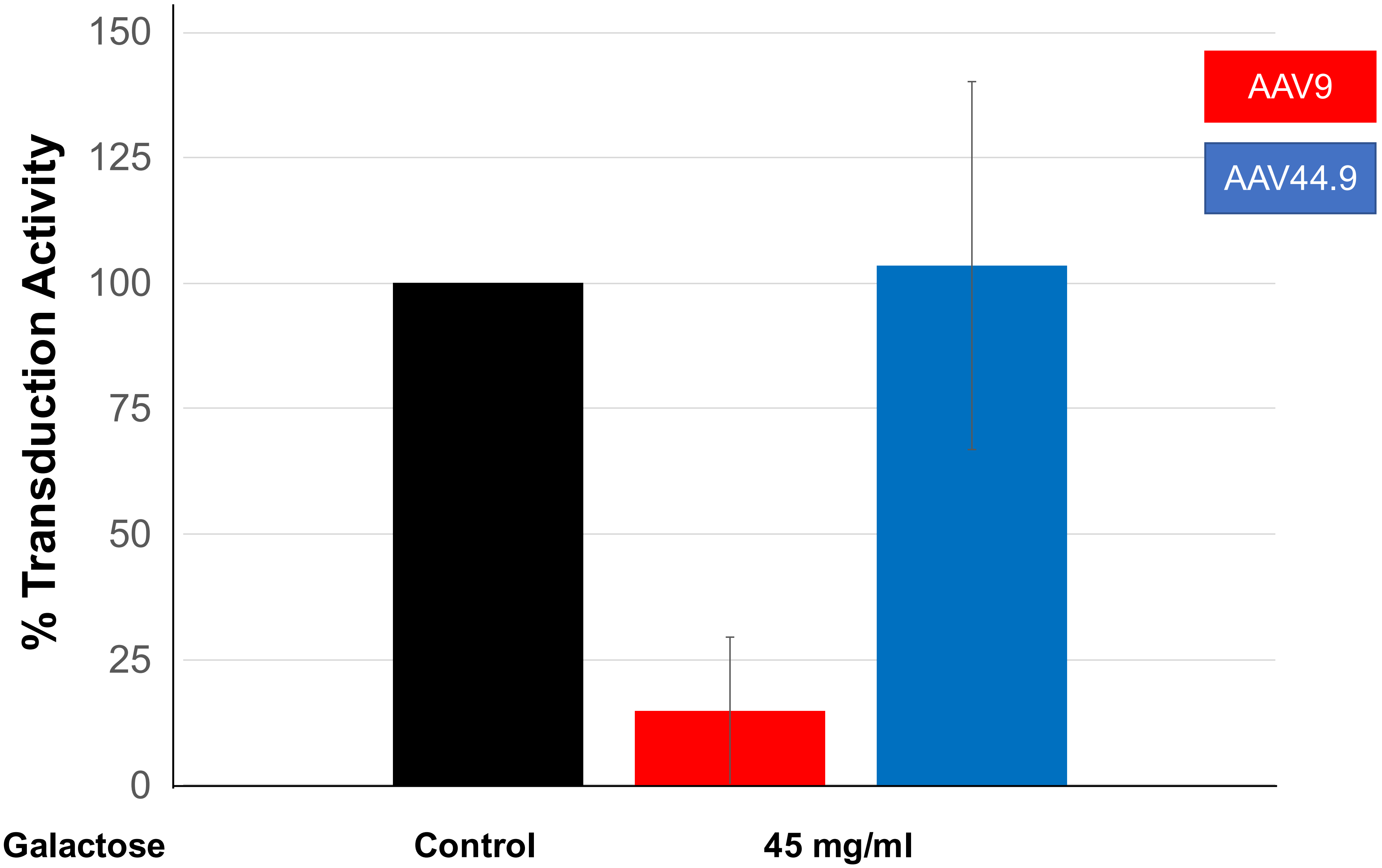

% Transduction Activity
150
125
100
75
50
25
0
AAV9
AAV44.9
Galactose
Control
45 mg/ml

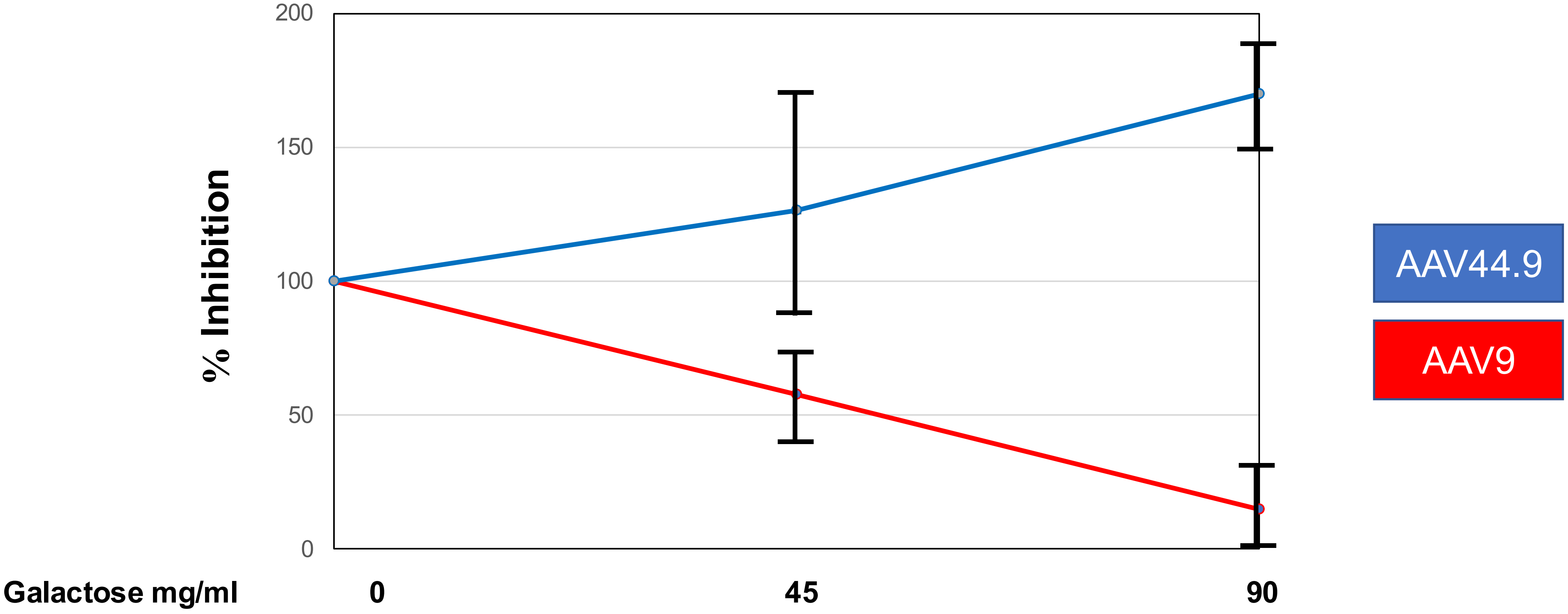

% Inhibition
200
150
100
50
0
Galactose mg/ml
0
45
90
AAV44.9
AAV9

Fig. 5A

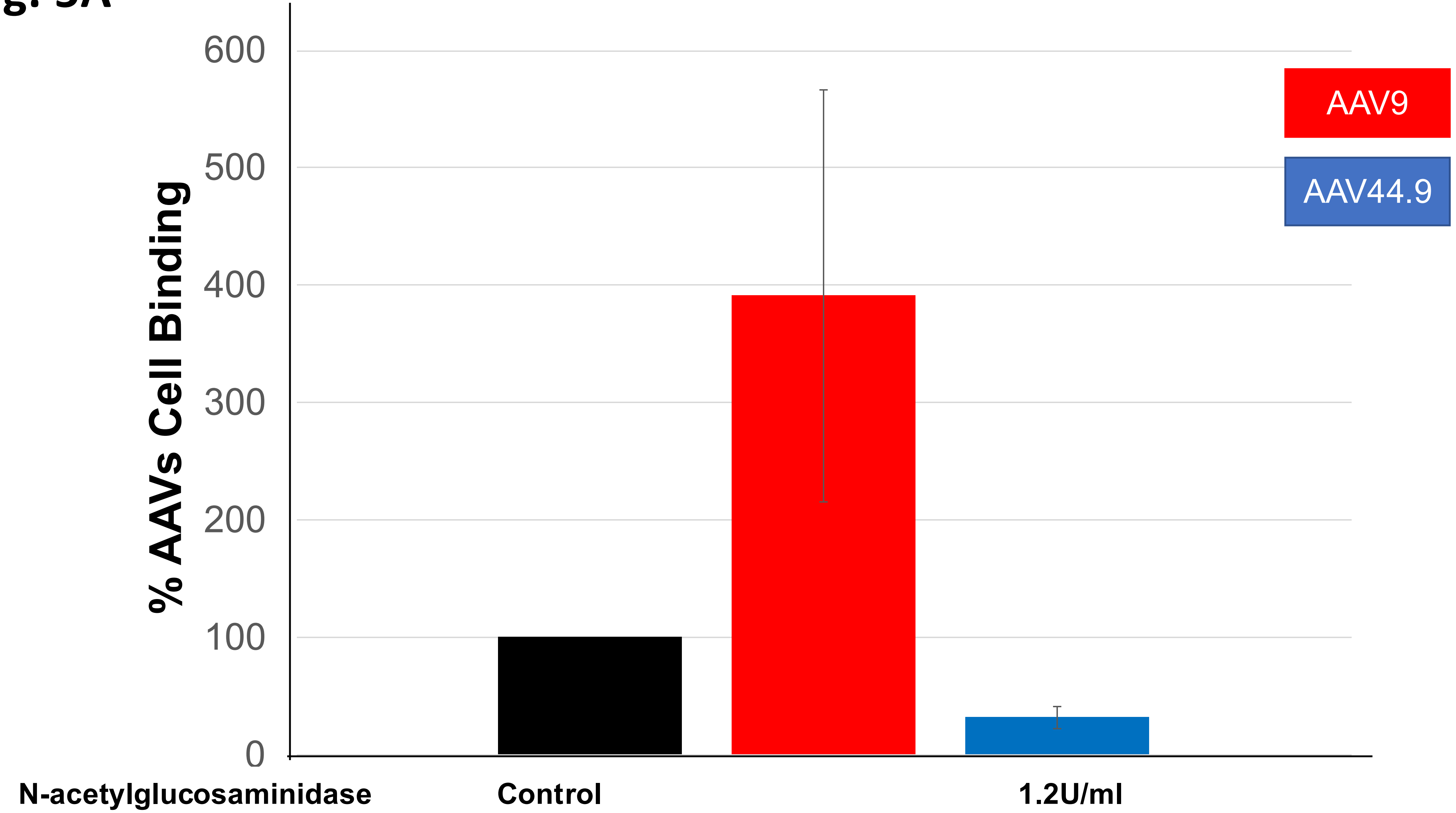
% AAVs Cell Binding
600
500
400
300
200
100
0
AAV9
AAV44.9
N-acetylglucosaminidase
Control
1.2U/ml

Table 1

AAV44.9	AA	AAVRh8
Thr (T)	179	Ser (S)
Ser (S)	473	Asn (N)
Ser (S)	483	Cys (C)
Glu (E)	531	Asp (D)

Fig. 6A

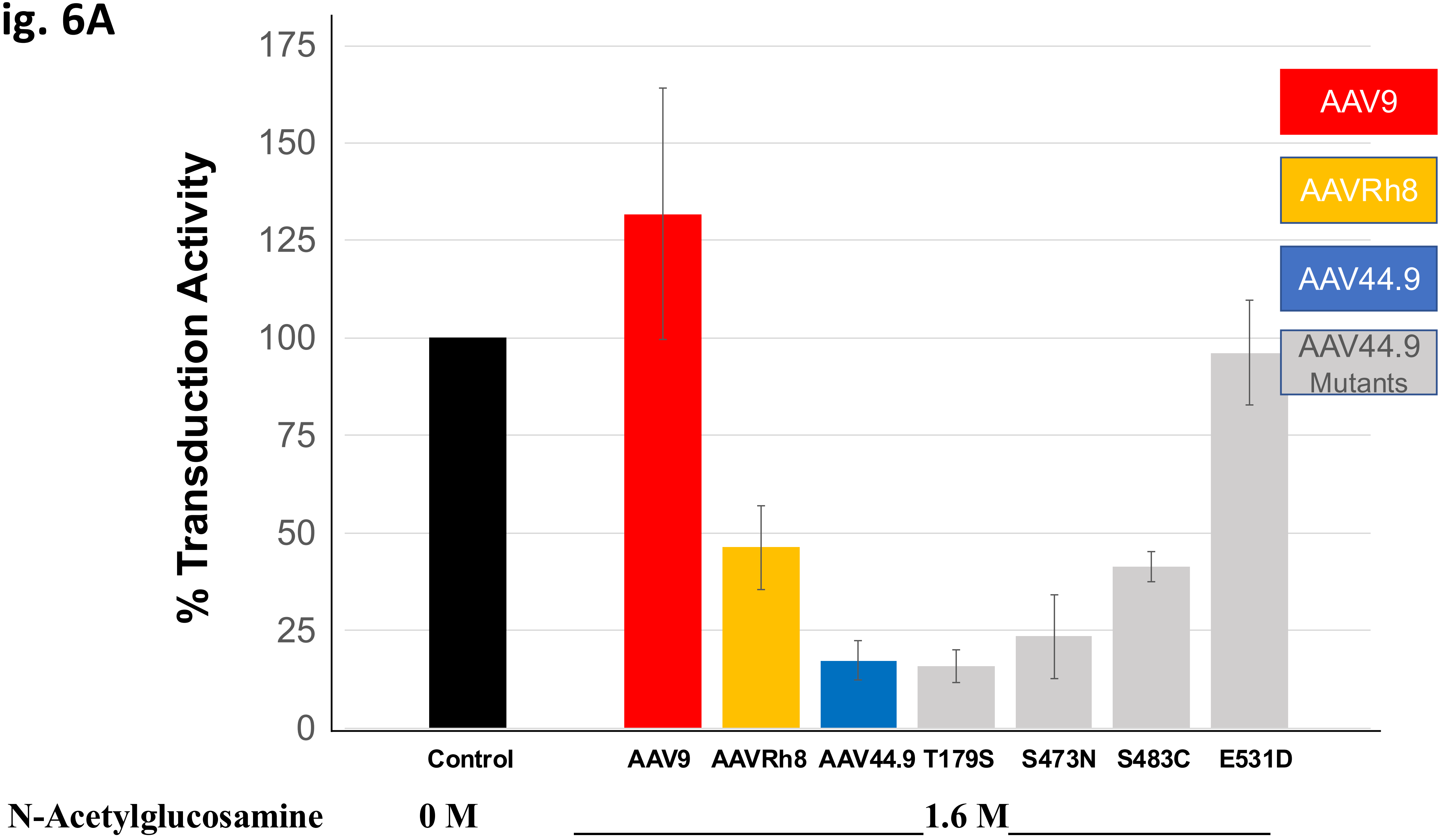
% Transduction Activity
175
150
125
100
75
50
25
0
AAV9
AAVRh8
AAV44.9
AAV44.9 Mutants
Control
AAV9
AAVRh8
AAV44.9
T179S
S473N
S483C
E531D
N-Acetylglucosamine
0 M
1.6 M

Fig. 6B

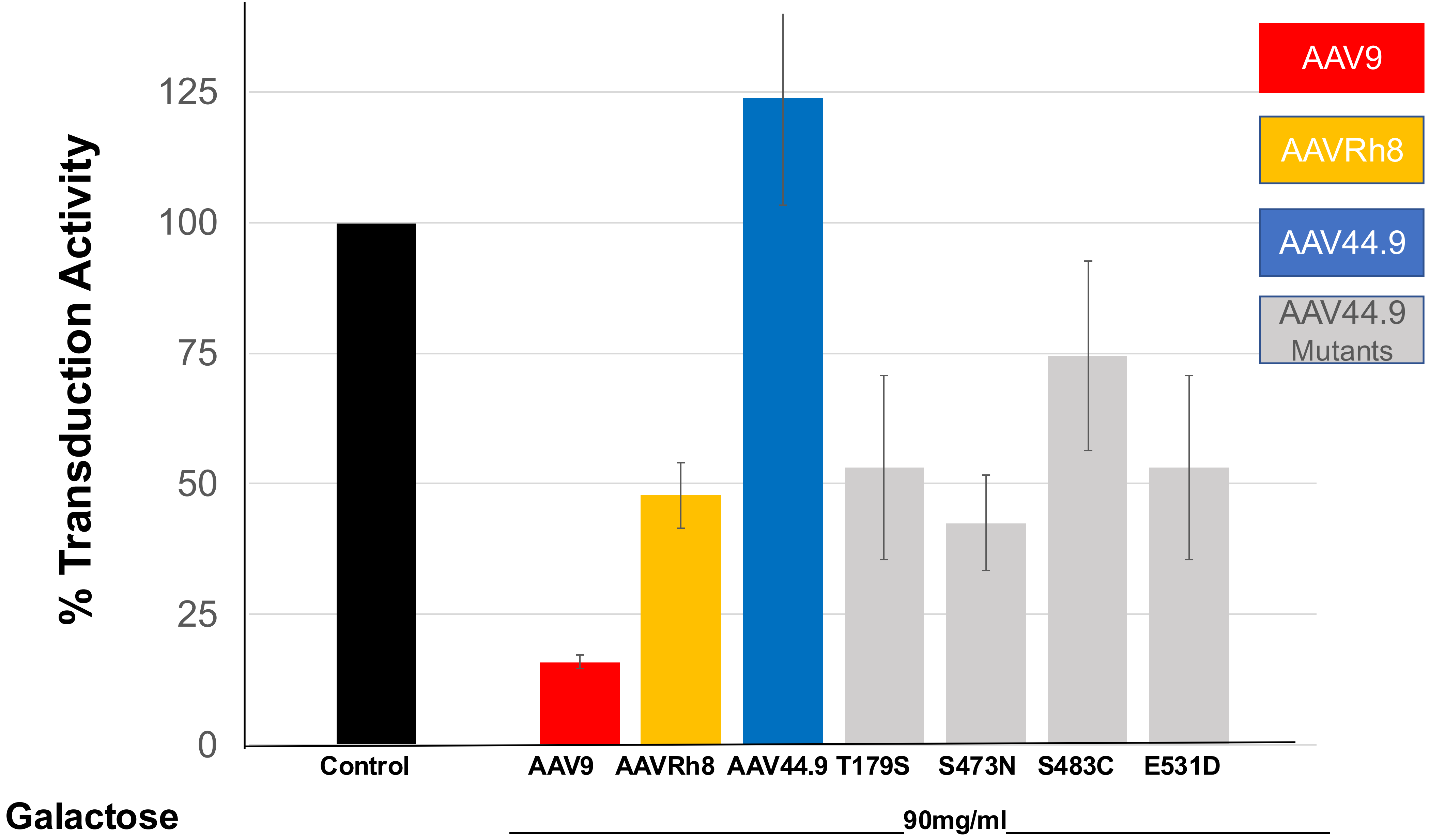

% Transduction Activity
125
100
75
50
25
0
AAV9
AAVRh8
AAV44.9
AAV44.9 Mutants
Control
AAV9
AAVRh8
AAV44.9
T179S
S473N
S483C
E531D
Galactose
90mg/ml

Fig. 7

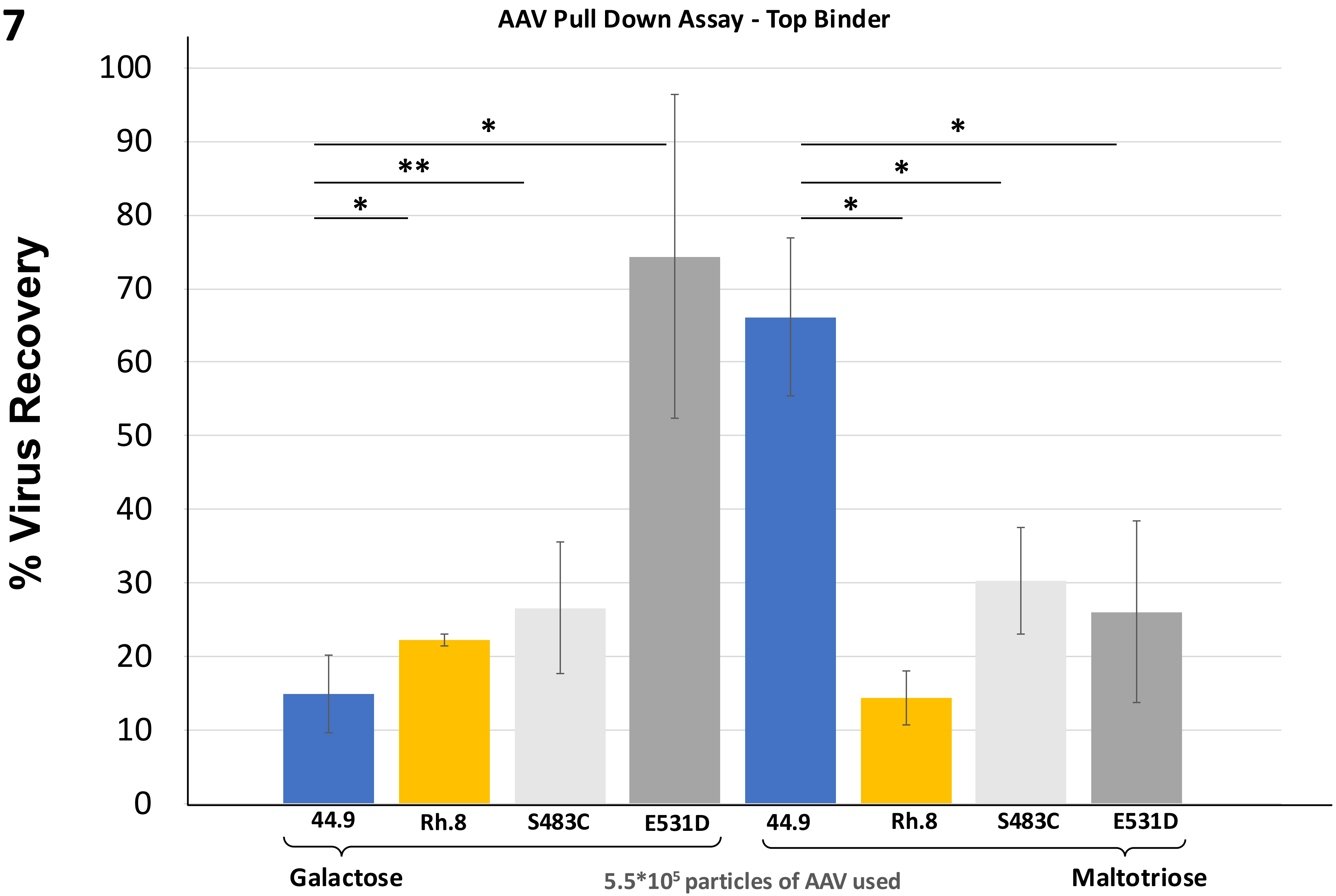
AAV Pull Down Assay - Top Binder
% Virus Recovery
100
90
80
70
60
50
40
30
20
10
0
*
**
*
*
*
*
44.9
Rh.8
S483C
E531D
44.9
Rh.8
S483C
E531D
Galactose
5.5*10^5 particles of AAV used
Maltotriose
