# Supplementary material for: Terminal glucose as a receptor for adeno-associated virus 44.9
Source: J Virol. 2026 Mar 23;100(4):e00254-25. doi: 10.1128/jvi.00254-25 (PMC13098211; doi:10.1128/jvi.00254-25)
Supplement: Table S1 — Glycan microarray data. [file jvi.00254-25-s0003.pdf]

ChartID	Glycan	Average
1	Gala-Sp8	18
2	Glca-Sp8	23
3	Mana-Sp8	17
4	GalNAca-Sp8	15
5	GalNAca-Sp15	21
6	Fuca-Sp8	16
7	Fuca-Sp9	22
8	Rhaa-Sp8	21
9	Neu5Aca-Sp8	18
10	Neu5Aca-Sp11	19
11	Neu5Acb-Sp8	19
12	Galb-Sp8	17
13	Glcb-Sp8	18
14	Manb-Sp8	13
15	GalNAcb-Sp8	19
16	GlcNAcb-Sp0	17
17	GlcNAcb-Sp8	22
18	GlcN(Gc)b-Sp8	19
19	Galb1-4GlcNAcb1-6(Galb1-4GlcNAcb1-3)GalNAca-Sp8	18
20	Galb1-4GlcNAcb1-6(Galb1-4GlcNAcb1-3)GalNAc-Sp14	19
21	GlcNAcb1-6(GlcNAcb1-4)(GlcNAcb1-3)GlcNAc-Sp8	20
22	6S(3S)Galb1-4(6S)GlcNAcb-Sp0	21
23	6S(3S)Galb1-4GlcNAcb-Sp0	21
24	(3S)Galb1-4(Fuca1-3)(6S)Glc-Sp0	24
25	(3S)Galb1-4Glcb-Sp8	16
26	(3S)Galb1-4(6S)Glcb-Sp0	18
27	(3S)Galb1-4(6S)Glcb-Sp8	19
28	(3S)Galb1-3(Fuca1-4)GlcNAcb-Sp8	18
29	(3S)Galb1-3GalNAca-Sp8	19
30	(3S)Galb1-3GlcNAcb-Sp0	18
31	(3S)Galb1-3GlcNAcb-Sp8	17
32	(3S)Galb1-4(Fuca1-3)GlcNAc-Sp0	20
33	(3S)Galb1-4(Fuca1-3)GlcNAc-Sp8	17
34	(3S)Galb1-4(6S)GlcNAcb-Sp0	20
35	(3S)Galb1-4(6S)GlcNAcb-Sp8	19
36	(3S)Galb1-4GlcNAcb-Sp0	19
37	(3S)Galb1-4GlcNAcb-Sp8	17
38	(3S)Galb-Sp8	15
39	(6S)(4S)Galb1-4GlcNAcb-Sp0	16
40	(4S)Galb1-4GlcNAcb-Sp8	21
41	(6P)Mana-Sp8	20
42	(6S)Galb1-4Glcb-Sp0	18
43	(6S)Galb1-4Glcb-Sp8	19
44	(6S)Galb1-4GlcNAcb-Sp8	19
45	(6S)Galb1-4(6S)Glcb-Sp8	18
46	Neu5Aca2-3(6S)Galb1-4GlcNAcb-Sp8	18
47	(6S)GlcNAcb-Sp8	23

48	Neu5,9Ac2a-Sp8	22
49	Neu5,9Ac2a2-6Galb1-4GlcNAcb-Sp8	17
50	Mana1-6(Mana1-3)Manb1-4GlcNAcb1-4GlcNAcb-Sp12	18
51	Mana1-6(Mana1-3)Manb1-4GlcNAcb1-4GlcNAcb-Sp13	17
52	GlcNAcb1-2Mana1-6(GlcNAcb1-2Mana1-3)Manb1-4GlcNAcb1-4GlcNAcb-Sp12	18
53	GlcNAcb1-2Mana1-6(GlcNAcb1-2Mana1-3)Manb1-4GlcNAcb1-4GlcNAcb-Sp13	18
54	Galb1-4GlcNAcb1-2Mana1-6(Galb1-4GlcNAcb1-2Mana1-3)Manb1-4GlcNAcb1-4G	18
55	Neu5Aca2-6Galb1-4GlcNAcb1-2Mana1-6(Neu5Aca2-6Galb1-4GlcNAcb1-2Mana1-	19
56	Neu5Aca2-6Galb1-4GlcNAcb1-2Mana1-6(Neu5Aca2-6Galb1-4GlcNAcb1-2Man-a1	19
57	Neu5Aca2-6Galb1-4GlcNAcb1-2Mana1-6(Neu5Aca2-6Galb1-4GlcNAcb1-2Mana1-	18
58	Fuca1-2Galb1-3GalNAcb1-3Gala-Sp9	22
59	Fuca1-2Galb1-3GalNAcb1-3Gala1-4Galb1-4Glcb-Sp9	18
60	Fuca1-2Galb1-3(Fuca1-4)GlcNAcb-Sp8	20
61	Fuca1-2Galb1-3GalNAca-Sp8	17
62	Fuca1-2Galb1-3GalNAca-Sp14	17
63	Fuca1-2Galb1-3GalNAcb1-4(Neu5Aca2-3)Galb1-4Glcb-Sp0	17
64	Fuca1-2Galb1-3GalNAcb1-4(Neu5Aca2-3)Galb1-4Glcb-Sp9	20
65	Fuca1-2Galb1-3GlcNAcb1-3Galb1-4Glcb-Sp8	20
66	Fuca1-2Galb1-3GlcNAcb1-3Galb1-4Glcb-Sp10	21
67	Fuca1-2Galb1-3GlcNAcb-Sp0	17
68	Fuca1-2Galb1-3GlcNAcb-Sp8	18
69	Fuca1-2Galb1-4(Fuca1-3)GlcNAcb1-3Galb1-4(Fuca1-3)GlcNAcb-Sp0	21
70	Fuca1-2Galb1-4(Fuca1-3)GlcNAcb1-3Galb1-4(Fuca1-3)GlcNAcb1-3Galb1-4(Fuca1	19
71	Fuca1-2Galb1-4(Fuca1-3)GlcNAcb-Sp0	20
72	Fuca1-2Galb1-4(Fuca1-3)GlcNAcb-Sp8	19
73	Fuca1-2Galb1-4GlcNAcb1-3Galb1-4GlcNAcb-Sp0	19
74	Fuca1-2Galb1-4GlcNAcb1-3Galb1-4GlcNAcb1-3Galb1-4GlcNAcb-Sp0	17
75	Fuca1-2Galb1-4GlcNAcb-Sp0	22
76	Fuca1-2Galb1-4GlcNAcb-Sp8	21
77	Fuca1-2Galb1-4Glcb-Sp0	34
78	Fuca1-2Galb-Sp8	16
79	Fuca1-3GlcNAcb-Sp8	19
80	Fuca1-4GlcNAcb-Sp8	19
81	Fucb1-3GlcNAcb-Sp8	20
82	GalNAca1-3(Fuca1-2)Galb1-3GlcNAcb-Sp0	17
83	GalNAca1-3(Fuca1-2)Galb1-4(Fuca1-3)GlcNAcb-Sp0	23
84	(3S)Galb1-4(Fuca1-3)Glcb-Sp0	23
85	GalNAca1-3(Fuca1-2)Galb1-4GlcNAcb-Sp0	22
86	GalNAca1-3(Fuca1-2)Galb1-4GlcNAcb-Sp8	17
87	GalNAca1-3(Fuca1-2)Galb1-4Glcb-Sp0	17
88	GlcNAcb1-3Galb1-3GalNAca-Sp8	22
89	GalNAca1-3(Fuca1-2)Galb-Sp8	18
90	GalNAca1-3(Fuca1-2)Galb-Sp18	19
91	GalNAca1-3GalNAcb-Sp8	19
92	GalNAca1-3Galb-Sp8	20
93	GalNAca1-4(Fuca1-2)Galb1-4GlcNAcb-Sp8	19
94	GalNAcb1-3GalNAca-Sp8	23
95	GalNAcb1-3(Fuca1-2)Galb-Sp8	19

96	GalNAcb1-3Gala1-4Galb1-4GlcNAcb-Sp0	21
97	GalNAcb1-4(Fuca1-3)GlcNAcb-Sp0	16
98	GalNAcb1-4GlcNAcb-Sp0	16
99	GalNAcb1-4GlcNAcb-Sp8	19
100	Gala1-2Galb-Sp8	20
101	Gala1-3(Fuca1-2)Galb1-3GlcNAcb-Sp0	18
102	Gala1-3(Fuca1-2)Galb1-3GlcNAcb-Sp8	19
103	Gala1-3(Fuca1-2)Galb1-4(Fuca1-3)GlcNAcb-Sp0	19
104	Gala1-3(Fuca1-2)Galb1-4(Fuca1-3)GlcNAcb-Sp8	25
105	Gala1-3(Fuca1-2)Galb1-4GlcNAc-Sp0	17
106	Gala1-3(Fuca1-2)Galb1-4Glcb-Sp0	19
107	Gala1-3(Fuca1-2)Galb-Sp8	20
108	Gala1-3(Fuca1-2)Galb-Sp18	21
109	Gala1-4(Gala1-3)Galb1-4GlcNAcb-Sp8	19
110	Gala1-3GalNAca-Sp8	17
111	Gala1-3GalNAca-Sp16	15
112	Gala1-3GalNAcb-Sp8	26
113	Gala1-3Galb1-4(Fuca1-3)GlcNAcb-Sp8	21
114	Gala1-3Galb1-3GlcNAcb-Sp0	14
115	Gala1-3Galb1-4GlcNAcb-Sp8	22
116	Gala1-3Galb1-4Glcb-Sp0	17
117	Gala1-3Galb1-4Glc-Sp10	22
118	Gala1-3Galb-Sp8	21
119	Gala1-4(Fuca1-2)Galb1-4GlcNAcb-Sp8	19
120	Gala1-4Galb1-4GlcNAcb-Sp0	20
121	Gala1-4Galb1-4GlcNAcb-Sp8	14
122	Gala1-4Galb1-4Glcb-Sp0	16
123	Gala1-4GlcNAcb-Sp8	21
124	Gala1-6Glcb-Sp8	18
125	Galb1-2Galb-Sp8	19
126	Galb1-3(Fuca1-4)GlcNAcb1-3Galb1-4(Fuca1-3)GlcNAcb-Sp0	20
127	Galb1-3GlcNAcb1-3Galb1-4(Fuca1-3)GlcNAcb-Sp0	20
128	Galb1-3(Fuca1-4)GlcNAc-Sp0	21
129	Galb1-3(Fuca1-4)GlcNAc-Sp8	19
130	Fuca1-4(Galb1-3)GlcNAcb-Sp8	22
131	Galb1-4GlcNAcb1-6GalNAca-Sp8	19
132	Galb1-4GlcNAcb1-6GalNAc-Sp14	21
133	GlcNAcb1-6(Galb1-3)GalNAca-Sp8	19
134	GlcNAcb1-6(Galb1-3)GalNAca-Sp14	26
135	Neu5Aca2-6(Galb1-3)GalNAca-Sp8	22
136	Neu5Aca2-6(Galb1-3)GalNAca-Sp14	17
137	Neu5Acb2-6(Galb1-3)GalNAca-Sp8	16
138	Neu5Aca2-6(Galb1-3)GlcNAcb1-4Galb1-4Glcb-Sp10	23
139	Galb1-3GalNAca-Sp8	16
140	Galb1-3GalNAca-Sp14	18
141	Galb1-3GalNAca-Sp16	25
142	Galb1-3GalNAcb-Sp8	20
143	Galb1-3GalNAcb1-3Gala1-4Galb1-4Glcb-Sp0	20

144	Galb1-3GalNAcb1-4(Neu5Aca2-3)Galb1-4Glcb-Sp0	14
145	Galb1-3GalNAcb1-4Galb1-4Glcb-Sp8	15
146	Galb1-3Galb-Sp8	20
147	Galb1-3GlcNAcb1-3Galb1-4GlcNAcb-Sp0	18
148	Galb1-3GlcNAcb1-3Galb1-4Glcb-Sp10	18
149	Galb1-3GlcNAcb-Sp0	19
150	Galb1-3GlcNAcb-Sp8	18
151	Galb1-4(Fuca1-3)GlcNAcb-Sp0	38
152	Galb1-4(Fuca1-3)GlcNAcb-Sp8	20
153	Galb1-4(Fuca1-3)GlcNAcb1-3Galb1-4(Fuca1-3)GlcNAcb-Sp0	21
154	Galb1-4(Fuca1-3)GlcNAcb1-3Galb1-4(Fuca1-3)GlcNAcb1-3Galb1-4(Fuca1-3)GlcN[illegible]	18
155	Galb1-4(6S)Glcb-Sp0	18
156	Galb1-4(6S)Glcb-Sp8	18
157	Galb1-4GalNAca1-3(Fuca1-2)Galb1-4GlcNAcb-Sp8	18
158	Galb1-4GalNAcb1-3(Fuca1-2)Galb1-4GlcNAcb-Sp8	15
159	Galb1-4GlcNAcb1-3GalNAca-Sp8	19
160	Galb1-4GlcNAcb1-3GalNAc-Sp14	15
161	Galb1-4GlcNAcb1-3Galb1-4(Fuca1-3)GlcNAcb1-3Galb1-4(Fuca1-3)GlcNAcb-Sp0	20
162	Galb1-4GlcNAcb1-3Galb1-4GlcNAcb1-3Galb1-4GlcNAcb-Sp0	18
163	Galb1-4GlcNAcb1-3Galb1-4GlcNAcb-Sp0	21
164	Galb1-4GlcNAcb1-3Galb1-4Glcb-Sp0	22
165	Galb1-4GlcNAcb1-3Galb1-4Glcb-Sp8	20
166	Galb1-4GlcNAcb1-6(Galb1-3)GalNAca-Sp8	20
167	Galb1-4GlcNAcb1-6(Galb1-3)GalNAc-Sp14	14
168	Galb1-4GlcNAcb-Sp0	17
169	Galb1-4GlcNAcb-Sp8	15
170	Galb1-4GlcNAcb-Sp23	18
171	Galb1-4Glcb-Sp0	17
172	Galb1-4Glcb-Sp8	18
173	GlcNAca1-3Galb1-4GlcNAcb-Sp8	16
174	GlcNAca1-6Galb1-4GlcNAcb-Sp8	19
175	GlcNAcb1-2Galb1-3GalNAca-Sp8	21
176	GlcNAcb1-6(GlcNAcb1-3)GalNAca-Sp8	21
177	GlcNAcb1-6(GlcNAcb1-3)GalNAca-Sp14	22
178	GlcNAcb1-6(GlcNAcb1-3)Galb1-4GlcNAcb-Sp8	20
179	GlcNAcb1-3GalNAca-Sp8	22
180	GlcNAcb1-3GalNAca-Sp14	17
181	GlcNAcb1-3Galb-Sp8	17
182	GlcNAcb1-3Galb1-4GlcNAcb-Sp0	19
183	GlcNAcb1-3Galb1-4GlcNAcb-Sp8	16
184	GlcNAcb1-3Galb1-4GlcNAcb1-3Galb1-4GlcNAcb-Sp0	20
185	GlcNAcb1-3Galb1-4Glcb-Sp0	18
186	GlcNAcb1-4-MDPLys	18
187	GlcNAcb1-6(GlcNAcb1-4)GalNAca-Sp8	17
188	GlcNAcb1-4Galb1-4GlcNAcb-Sp8	17
189	GlcNAcb1-4GlcNAcb1-4GlcNAcb1-4GlcNAcb1-4GlcNAcb1-4GlcNAcb1-Sp8	22
190	GlcNAcb1-4GlcNAcb1-4GlcNAcb1-4GlcNAcb1-4GlcNAcb1-Sp8	20
191	GlcNAcb1-4GlcNAcb1-4GlcNAcb-Sp8	20

192	GlcNAcb1-6GalNAca-Sp8	19
193	GlcNAcb1-6GalNAca-Sp14	19
194	GlcNAcb1-6Galb1-4GlcNAcb-Sp8	18
195	Glca1-4Glcb-Sp8	26
196	Glca1-4Glca-Sp8	20
197	Glca1-6Glca1-6Glcb-Sp8	19
198	Glcb1-4Glcb-Sp8	22
199	Glcb1-6Glcb-Sp8	25
200	G-ol-Sp8	22
201	GlcAa-Sp8	18
202	GlcAb-Sp8	24
203	GlcAb1-3Galb-Sp8	20
204	GlcAb1-6Galb-Sp8	23
205	KDNa2-3Galb1-3GlcNAcb-Sp0	20
206	KDNa2-3Galb1-4GlcNAcb-Sp0	21
207	Mana1-2Mana1-2Mana1-3Mana-Sp9	19
208	Mana1-2Mana1-6(Mana1-2Mana1-3)Mana-Sp9	17
209	Mana1-2Mana1-3Mana-Sp9	16
210	Mana1-6(Mana1-2Mana1-3)Mana1-6(Mana1-2Mana1-3)Manb1-4GlcNAcb1-4Glc	15
211	Mana1-2Mana1-6(Mana1-3)Mana1-6(Mana1-2Mana1-2Mana1-3)Manb1-4GlcNAc	21
212	Mana1-2Mana1-6(Mana1-2Mana1-3)Mana1-6(Mana1-2Mana1-2Mana1-3)Manb1-	20
213	Mana1-6(Mana1-3)Mana-Sp9	17
214	Mana1-2Mana1-2Mana1-6(Mana1-3)Mana-Sp9	19
215	Mana1-6(Mana1-3)Mana1-6(Mana1-2Mana1-3)Manb1-4GlcNAcb1-4GlcNAcb-Sp1	18
216	Mana1-6(Mana1-3)Mana1-6(Mana1-3)Manb1-4GlcNAcb1-4GlcNAcb-Sp12	24
217	Manb1-4GlcNAcb-Sp0	17
218	Neu5Aca2-3Galb1-4GlcNAcb1-3Galb1-4(Fuca1-3)GlcNAcb-Sp0	24
219	(3S)Galb1-4(Fuca1-3)(6S)GlcNAcb-Sp8	20
220	Fuca1-2(6S)Galb1-4GlcNAcb-Sp0	22
221	Fuca1-2Galb1-4(6S)GlcNAcb-Sp8	22
222	Fuca1-2(6S)Galb1-4(6S)Glcb-Sp0	21
223	Neu5Aca2-3Galb1-3GalNAca-Sp8	17
224	Neu5Aca2-3Galb1-3GalNAca-Sp14	20
225	GalNAcb1-4(Neu5Aca2-8Neu5Aca2-8Neu5Aca2-8Neu5Aca2-3)Galb1-4Glcb-Sp0	17
226	GalNAcb1-4(Neu5Aca2-8Neu5Aca2-8Neu5Aca2-3)Galb1-4Glcb-Sp0	19
227	Neu5Aca2-8Neu5Aca2-8Neu5Aca2-3Galb1-4Glcb-Sp0	20
228	GalNAcb1-4(Neu5Aca2-8Neu5Aca2-3)Galb1-4Glcb-Sp0	20
229	Neu5Aca2-8Neu5Aca2-8Neu5Aca-Sp8	20
230	GalNAcb1-4(Neu5Aca2-3)Galb1-4GlcNAcb-Sp0	14
231	GalNAcb1-4(Neu5Aca2-3)Galb1-4GlcNAcb-Sp8	16
232	GalNAcb1-4(Neu5Aca2-3)Galb1-4Glcb-Sp0	18
233	Neu5Aca2-3Galb1-3GalNAcb1-4(Neu5Aca2-3)Galb1-4Glcb-Sp0	19
234	Neu5Aca2-6(Neu5Aca2-3)GalNAca-Sp8	18
235	Neu5Aca2-3GalNAca-Sp8	15
236	Neu5Aca2-3GalNAcb1-4GlcNAcb-Sp0	17
237	Neu5Aca2-3Galb1-3(6S)GlcNAc-Sp8	16
238	Neu5Aca2-3Galb1-3(Fuca1-4)GlcNAcb-Sp8	17
239	Neu5Aca2-3Galb1-3(Fuca1-4)GlcNAcb1-3Galb1-4(Fuca1-3)GlcNAcb-Sp0	21

240	Neu5Aca2-3Galb1-4(Neu5Aca2-3Galb1-3)GlcNAcb-Sp8	18
241	Neu5Aca2-3Galb1-3(6S)GalNAca-Sp8	21
242	Neu5Aca2-6(Neu5Aca2-3Galb1-3)GalNAca-Sp8	18
243	Neu5Aca2-6(Neu5Aca2-3Galb1-3)GalNAca-Sp14	20
244	Neu5Aca2-3Galb-Sp8	17
245	Neu5Aca2-3Galb1-3GalNAcb1-3Gala1-4Galb1-4Glcb-Sp0	21
246	Neu5Aca2-3Galb1-3GlcNAcb1-3Galb1-4GlcNAcb-Sp0	16
247	Fuca1-2(6S)Galb1-4Glcb-Sp0	23
248	Neu5Aca2-3Galb1-3GlcNAcb-Sp0	20
249	Neu5Aca2-3Galb1-4(6S)GlcNAcb-Sp8	19
250	Neu5Aca2-3Galb1-4(Fuca1-3)(6S)GlcNAcb-Sp8	18
251	Neu5Aca2-3Galb1-4(Fuca1-3)GlcNAcb1-3Galb1-4(Fuca1-3)GlcNAcb1-3Galb1-4(Fu	20
252	Neu5Aca2-3Galb1-4(Fuca1-3)GlcNAcb-Sp0	18
253	Neu5Aca2-3Galb1-4(Fuca1-3)GlcNAcb-Sp8	16
254	Neu5Aca2-3Galb1-4(Fuca1-3)GlcNAcb1-3Galb-Sp8	15
255	Neu5Aca2-3Galb1-4(Fuca1-3)GlcNAcb1-3Galb1-4GlcNAcb-Sp8	24
256	Neu5Aca2-3Galb1-4GlcNAcb1-3Galb1-4GlcNAcb1-3Galb1-4GlcNAcb-Sp0	19
257	Neu5Aca2-3Galb1-4GlcNAcb-Sp0	20
258	Neu5Aca2-3Galb1-4GlcNAcb-Sp8	16
259	Neu5Aca2-3Galb1-4GlcNAcb1-3Galb1-4GlcNAcb-Sp0	21
260	Fuca1-2Galb1-4(6S)Glcb-Sp0	21
261	Neu5Aca2-3Galb1-4Glcb-Sp0	13
262	Neu5Aca2-3Galb1-4Glcb-Sp8	25
263	Neu5Aca2-6GalNAca-Sp8	20
264	Neu5Aca2-6GalNAcb1-4GlcNAcb-Sp0	21
265	Neu5Aca2-6Galb1-4(6S)GlcNAcb-Sp8	18
266	Neu5Aca2-6Galb1-4GlcNAcb-Sp0	20
267	Neu5Aca2-6Galb1-4GlcNAcb-Sp8	21
268	Neu5Aca2-6Galb1-4GlcNAcb1-3Galb1-4(Fuca1-3)GlcNAcb1-3Galb1-4(Fuca1-3)Gl	20
269	Neu5Aca2-6Galb1-4GlcNAcb1-3Galb1-4GlcNAcb-Sp0	17
270	Neu5Aca2-6Galb1-4Glcb-Sp0	16
271	Neu5Aca2-6Galb1-4Glcb-Sp8	17
272	Neu5Aca2-6Galb-Sp8	18
273	Neu5Aca2-8Neu5Aca-Sp8	21
274	Neu5Aca2-8Neu5Aca2-3Galb1-4Glcb-Sp0	14
275	Galb1-3(Fuca1-4)GlcNAcb1-3Galb1-3(Fuca1-4)GlcNAcb-Sp0	17
276	Neu5Acb2-6GalNAca-Sp8	17
277	Neu5Acb2-6Galb1-4GlcNAcb-Sp8	17
278	Neu5Gca2-3Galb1-3(Fuca1-4)GlcNAcb-Sp0	18
279	Neu5Gca2-3Galb1-3GlcNAcb-Sp0	30
280	Neu5Gca2-3Galb1-4(Fuca1-3)GlcNAcb-Sp0	27
281	Neu5Gca2-3Galb1-4GlcNAcb-Sp0	21
282	Neu5Gca2-3Galb1-4Glcb-Sp0	22
283	Neu5Gca2-6GalNAca-Sp0	22
284	Neu5Gca2-6Galb1-4GlcNAcb-Sp0	19
285	Neu5Gca-Sp8	25
286	Neu5Aca2-3Galb1-4GlcNAcb1-6(Galb1-3)GalNAca-Sp14	19
287	Galb1-3GlcNAcb1-3Galb1-3GlcNAcb-Sp0	22

288	Galb1-4(Fuca1-3)(6S)GlcNAcb-Sp0	20
289	Galb1-4(Fuca1-3)(6S)Glcb-Sp0	16
290	Galb1-4(Fuca1-3)GlcNAcb1-3Galb1-3(Fuca1-4)GlcNAcb-Sp0	22
291	Galb1-4GlcNAcb1-3Galb1-3GlcNAcb-Sp0	18
292	Neu5Aca2-3Galb1-3GlcNAcb1-3Galb1-3GlcNAcb-Sp0	27
293	Neu5Aca2-3Galb1-4GlcNAcb1-3Galb1-3GlcNAcb-Sp0	20
294	4S(3S)Galb1-4GlcNAcb-Sp0	19
295	(6S)Galb1-4(6S)GlcNAcb-Sp0	20
296	(6P)Glcb-Sp10	25
297	Neu5Aca2-3Galb1-4(Fuca1-3)GlcNAcb1-6(Galb1-3)GalNAca-Sp14	24
298	Galb1-3Galb1-4GlcNAcb-Sp8	22
299	Neu5Aca2-6Galb1-4GlcNAcb1-2Mana1-6(Galb1-4GlcNAcb1-2Mana1-3)Manb1-4G	16
300	Galb1-4GlcNAcb1-6(Galb1-4GlcNAcb1-3)Galb1-4GlcNAc-Sp0	18
301	GlcNAcb1-6(Galb1-4GlcNAcb1-3)Galb1-4GlcNAc-Sp0	17
302	Galb1-4GlcNAca1-6Galb1-4GlcNAcb-Sp0	23
303	Galb1-4GlcNAcb1-6Galb1-4GlcNAcb-Sp0	41
304	GalNAcb1-3Galb-Sp8	27
305	GlcAb1-3GlcNAcb-Sp8	21
306	Neu5Aca2-6Galb1-4GlcNAcb1-2Mana1-6(GlcNAcb1-2Mana1-3)Manb1-4GlcNAcb	21
307	GlcNAcb1-3Man-Sp10	18
308	GlcNAcb1-4GlcNAcb-Sp10	19
309	GlcNAcb1-4GlcNAcb-Sp12	16
310	MurNAcb1-4GlcNAcb-Sp10	19
311	Mana1-6Manb-Sp10	17
312	Mana1-6(Mana1-3)Mana1-6(Mana1-3)Manb-Sp10	18
313	Mana1-2Mana1-6(Mana1-3)Mana1-6(Mana1-2Mana1-2Mana1-3)Mana-Sp9	20
314	Mana1-2Mana1-6(Mana1-2Mana1-3)Mana1-6(Mana1-2Mana1-2Mana1-3)Mana-S	19
315	Neu5Aca2-3Galb1-4GlcNAcb1-6(Neu5Aca2-3Galb1-3)GalNAca-Sp14	17
316	Neu5Aca2-6Galb1-4GlcNAcb1-2Mana1-6(Neu5Aca2-3Galb1-4GlcNAcb1-2Mana1-	18
317	Galb1-4GlcNAcb1-2Mana1-6(Neu5Aca2-6Galb1-4GlcNAcb1-2Mana1-3)Manb1-4G	19
318	Neu5Aca2-8Neu5Acb-Sp17	21
319	Neu5Aca2-8Neu5Aca2-8Neu5Acb-Sp8	19
320	Neu5Gcb2-6Galb1-4GlcNAc-Sp8	21
321	Galb1-3GlcNAcb1-2Mana1-6(Galb1-3GlcNAcb1-2Mana1-3)Manb1-4GlcNAcb1-4G	20
322	Neu5Aca2-3Galb1-4GlcNAcb1-2Mana1-6(Neu5Aca2-3Galb1-4GlcNAcb1-2Mana1-	26
323	Neu5Aca2-3Galb1-4GlcNAcb1-2Mana1-6(Neu5Aca2-6Galb1-4GlcNAcb1-2Mana1-	20
324	Galb1-4(Fuca1-3)GlcNAcb1-2Mana1-6(Galb1-4(Fuca1-3)GlcNAcb1-2Mana1-3)Ma	18
325	Neu5,9Ac2a2-3Galb1-4GlcNAcb-Sp0	18
326	Neu5,9Ac2a2-3Galb1-3GlcNAcb-Sp0	21
327	Neu5Aca2-6Galb1-4GlcNAcb1-3Galb1-3GlcNAcb-Sp0	16
328	Neu5Aca2-3Galb1-3(Fuca1-4)GlcNAcb1-3Galb1-3(Fuca1-4)GlcNAcb-Sp0	22
329	Neu5Aca2-6Galb1-4GlcNAcb1-3Galb1-4GlcNAcb1-3Galb1-4GlcNAcb-Sp0	18
330	Gala1-4Galb1-4GlcNAcb1-3Galb1-4Glcb-Sp0	19
331	GalNAcb1-3Gala1-4Galb1-4GlcNAcb1-3Galb1-4Glcb-Sp0	19
332	GalNAca1-3(Fuca1-2)Galb1-4GlcNAcb1-3Galb1-4GlcNAcb-Sp0	19
333	GalNAca1-3(Fuca1-2)Galb1-4GlcNAcb1-3Galb1-4GlcNAcb1-3Galb1-4GlcNAcb-Sp0	20
334	Neu5Aca2-3Galb1-4(Fuca1-3)GlcNAcb1-6(Neu5Aca2-3Galb1-3)GalNAc-Sp14	31
335	GlcNAca1-4Galb1-4GlcNAcb1-3Galb1-4GlcNAcb1-3Galb1-4GlcNAcb-Sp0	20

336	GlcNAca1-4Galb1-4GlcNAcb-Sp0	15
337	GlcNAca1-4Galb1-3GlcNAcb-Sp0	20
338	GlcNAca1-4Galb1-4GlcNAcb1-3Galb1-4Glcb-Sp0	18
339	GlcNAca1-4Galb1-4GlcNAcb1-3Galb1-4(Fuca1-3)GlcNAcb1-3Galb1-4(Fuca1-3)Glc	23
340	GlcNAca1-4Galb1-4GlcNAcb1-3Galb1-4GlcNAcb-Sp0	18
341	GlcNAca1-4Galb1-3GalNAc-Sp14	22
342	Neu5Aca2-6Galb1-4GlcNAcb1-2Mana1-6(Mana1-3)Manb1-4GlcNAcb1-4GlcNAc-S	19
343	Mana1-6(Neu5Aca2-6Galb1-4GlcNAcb1-2Mana1-3)Manb1-4GlcNAcb1-4GlcNAc-S	16
344	Neu5Aca2-6Galb1-4GlcNAcb1-2Mana1-6Manb1-4GlcNAcb1-4GlcNAc-Sp12	21
345	Neu5Aca2-6Galb1-4GlcNAcb1-2Mana1-3Manb1-4GlcNAcb1-4GlcNAc-Sp12	29
346	Galb1-4GlcNAcb1-2Mana1-3Manb1-4GlcNAcb1-4GlcNAc-Sp12	17
347	Galb1-4GlcNAcb1-2Mana1-6Manb1-4GlcNAcb1-4GlcNAc-Sp12	25
348	Mana1-6(Galb1-4GlcNAcb1-2Mana1-3)Manb1-4GlcNAcb1-4GlcNAcb-Sp12	20
349	GlcNAcb1-2Mana1-6(GlcNAcb1-2Mana1-3)Manb1-4GlcNAcb1-4(Fuca1-6)GlcNAc	23
350	Galb1-4GlcNAcb1-2Mana1-6(Galb1-4GlcNAcb1-2Mana1-3)Manb1-4GlcNAcb1-4(	22
351	Galb1-3GlcNAcb1-2Mana1-6(Galb1-3GlcNAcb1-2Mana1-3)Manb1-4GlcNAcb1-4(	21
352	(6S)GlcNAcb1-3Galb1-4GlcNAcb-Sp0	17
353	KDNa2-3Galb1-4(Fuca1-3)GlcNAc-Sp0	19
354	KDNa2-6Galb1-4GlcNAc-Sp0	21
355	KDNa2-3Galb1-4Glc-Sp0	21
356	KDNa2-3Galb1-3GalNAca-Sp14	15
357	Fuca1-2Galb1-3GlcNAcb1-2Mana1-6(Fuca1-2Galb1-3GlcNAcb1-2Mana1-3)Manb	27
358	Fuca1-2Galb1-4GlcNAcb1-2Mana1-6(Fuca1-2Galb1-4GlcNAcb1-2Mana1-3)Manb	21
359	Fuca1-2Galb1-4(Fuca1-3)GlcNAcb1-2Mana1-6(Fuca1-2Galb1-4(Fuca1-3)GlcNAcb	20
360	Gala1-3Galb1-4GlcNAcb1-2Mana1-6(Gala1-3Galb1-4GlcNAcb1-2Mana1-3)Manb1	19
361	Galb1-4GlcNAcb1-2Mana1-6(Mana1-3)Manb1-4GlcNAcb1-4GlcNAcb-Sp12	17
362	Fuca1-4(Galb1-3)GlcNAcb1-2Mana1-6(Fuca1-4(Galb1-3)GlcNAcb1-2Mana1-3)Ma	23
363	Neu5Aca2-6GlcNAcb1-4GlcNAc-Sp21	17
364	Neu5Aca2-6GlcNAcb1-4GlcNAcb1-4GlcNAc-Sp21	21
365	Galb1-4(Fuca1-3)GlcNAcb1-6(Fuca1-2Galb1-4GlcNAcb1-3)Galb1-4Glc-Sp21	20
366	Galb1-4GlcNAcb1-2Mana1-6(Galb1-4GlcNAcb1-4(Galb1-4GlcNAcb1-2)Mana1-3)M	20
367	GalNAca1-3(Fuca1-2)Galb1-4GlcNAcb1-2Mana1-6(GalNAca1-3(Fuca1-2)Galb1-4G	15
368	Gala1-3(Fuca1-2)Galb1-4GlcNAcb1-2Mana1-6(Gala1-3(Fuca1-2)Galb1-4GlcNAcb1	19
369	Gala1-3Galb1-4(Fuca1-3)GlcNAcb1-2Mana1-6(Gala1-3Galb1-4(Fuca1-3)GlcNAcb1	18
370	GalNAca1-3(Fuca1-2)Galb1-3GlcNAcb1-2Mana1-6(GalNAca1-3(Fuca1-2)Galb1-3G	18
371	Fuca1-4(Fuca1-2Galb1-3)GlcNAcb1-2Mana1-3(Fuca1-4(Fuca1-2Galb1-3)GlcNAcb	21
372	Neu5Aca2-3Galb1-4GlcNAcb1-3GalNAc-Sp14	21
373	Neu5Aca2-6Galb1-4GlcNAcb1-3GalNAc-Sp14	18
374	Neu5Aca2-3Galb1-4(Fuca1-3)GlcNAcb1-3GalNAca-Sp14	30
375	GalNAcb1-4GlcNAcb1-2Mana1-6(GalNAcb1-4GlcNAcb1-2Mana1-3)Manb1-4GlcN	25
376	Galb1-3GalNAca1-3(Fuca1-2)Galb1-4Glc-Sp0	19
377	Galb1-3GalNAca1-3(Fuca1-2)Galb1-4GlcNAc-Sp0	25
378	Galb1-3GlcNAcb1-3Galb1-4GlcNAcb1-6(Galb1-3GlcNAcb1-3)Galb1-4Glcb-Sp0	18
379	Galb1-4(Fuca1-3)GlcNAcb1-6(Galb1-3GlcNAcb1-3)Galb1-4Glc-Sp21	16
380	Galb1-4GlcNAcb1-6(Fuca1-4(Fuca1-2Galb1-3)GlcNAcb1-3)Galb1-4Glc-Sp21	19
381	Galb1-4(Fuca1-3)GlcNAcb1-6(Fuca1-4(Fuca1-2Galb1-3)GlcNAcb1-3)Galb1-4Glc-S	16
382	Galb1-3GlcNAcb1-3Galb1-4(Fuca1-3)GlcNAcb1-6(Galb1-3GlcNAcb1-3)Galb1-4Glc	16
383	Galb1-4GlcNAcb1-6(Galb1-4GlcNAcb1-2)Mana1-6(Galb1-4GlcNAcb1-4(Galb1-4G	21

384	GlcNAcb1-2Mana1-6(GlcNAcb1-4(GlcNAcb1-2)Mana1-3)Manb1-4GlcNAcb1-4Glc	20
385	Fuca1-2Galb1-3GalNAca1-3(Fuca1-2)Galb1-4Glcb-Sp0	21
386	Fuca1-2Galb1-3GalNAca1-3(Fuca1-2)Galb1-4GlcNAcb-Sp0	22
387	Galb1-3GlcNAcb1-3GalNAca-Sp14	18
388	GalNAcb1-4(Neu5Aca2-3)Galb1-4GlcNAcb1-3GalNAca-Sp14	23
389	GalNAca1-3(Fuca1-2)Galb1-3GalNAca1-3(Fuca1-2)Galb1-4GlcNAcb-Sp0	64
390	Gala1-3Galb1-3GlcNAcb1-2Mana1-6(Gala1-3Galb1-3GlcNAcb1-2Mana1-3)Manb1	24
391	Gala1-3Galb1-3(Fuca1-4)GlcNAcb1-2Mana1-6(Gala1-3Galb1-3(Fuca1-4)GlcNAcb1	23
392	Neu5Aca2-3Galb1-3GlcNAcb1-2Mana1-6(Neu5Aca2-3Galb1-3GlcNAcb1-2Mana1-	15
393	GlcNAcb1-2Mana1-6(Galb1-4GlcNAcb1-2Mana1-3)Manb1-4GlcNAcb1-4GlcNAc-S	18
394	Galb1-4GlcNAcb1-2Mana1-6(GlcNAcb1-2Mana1-3)Manb1-4GlcNAcb1-4GlcNAc-S	20
395	Neu5Aca2-3Galb1-3GlcNAcb1-3GalNAca-Sp14	22
396	Fuca1-2Galb1-4GlcNAcb1-3GalNAca-Sp14	21
397	Galb1-4(Fuca1-3)GlcNAcb1-3GalNAca-Sp14	21
398	GalNAca1-3GalNAcb1-3Gala1-4Galb1-4GlcNAcb-Sp0	16
399	Gala1-4Galb1-3GlcNAcb1-2Mana1-6(Gala1-4Galb1-3GlcNAcb1-2Mana1-3)Manb1	22
400	Gala1-4Galb1-4GlcNAcb1-2Mana1-6(Gala1-4Galb1-4GlcNAcb1-2Mana1-3)Manb1	19
401	Gala1-3Galb1-4GlcNAcb1-3GalNAca-Sp14	18
402	Galb1-3GlcNAcb1-6Galb1-4GlcNAcb-Sp0	18
403	Galb1-3GlcNAca1-6Galb1-4GlcNAcb-Sp0	22
404	GalNAcb1-3Gala1-6Galb1-4Glcb-Sp8	19
405	Gala1-3(Fuca1-2)Galb1-4(Fuca1-3)Glcb-Sp21	19
406	Galb1-4GlcNAcb1-6(Neu5Aca2-6Galb1-3GlcNAcb1-3)Galb1-4Glc-Sp21	23
407	Galb1-3GalNAcb1-4(Neu5Aca2-8Neu5Aca2-3)Galb1-4Glcb-Sp0	22
408	Neu5Aca2-3Galb1-3GalNAcb1-4(Neu5Aca2-8Neu5Aca2-3)Galb1-4Glcb-Sp0	18
409	Gala1-3(Fuca1-2)Galb1-4GlcNAcb1-3GalNAca-Sp14	17
410	GalNAca1-3(Fuca1-2)Galb1-4GlcNAcb1-3GalNAca-Sp14	19
411	GalNAca1-3GalNAcb1-3Gala1-4Galb1-4Glcb-Sp0	18
412	Fuca1-2Galb1-4(Fuca1-3)GlcNAcb1-3GalNAca-Sp14	25
413	Gala1-3(Fuca1-2)Galb1-4(Fuca1-3)GlcNAcb1-3GalNAc-Sp14	23
414	GalNAca1-3(Fuca1-2)Galb1-4(Fuca1-3)GlcNAcb1-3GalNAc-Sp14	25
415	Galb1-4(Fuca1-3)GlcNAcb1-2Mana1-6(Galb1-4(Fuca1-3)GlcNAcb1-2Mana1-3)Ma	20
416	Fuca1-2Galb1-4GlcNAcb1-2Mana1-6(Fuca1-2Galb1-4GlcNAcb1-2Mana1-3)Manb	25
417	GlcNAcb1-2(GlcNAcb1-6)Mana1-6(GlcNAcb1-2Mana1-3)Manb1-4GlcNAcb1-4Glc	48
418	Fuca1-2Galb1-3GlcNAcb1-3GalNAc-Sp14	18
419	Gala1-3(Fuca1-2)Galb1-3GlcNAcb1-3GalNAc-Sp14	19
420	GalNAca1-3(Fuca1-2)Galb1-3GlcNAcb1-3GalNAc-Sp14	24
421	Gala1-3Galb1-3GlcNAcb1-3GalNAc-Sp14	21
422	Fuca1-2Galb1-3GlcNAcb1-2Mana1-6(Fuca1-2Galb1-3GlcNAcb1-2Mana1-3)Manb	27
423	Gala1-3(Fuca1-2)Galb1-4GlcNAcb1-2Mana1-6(Gala1-3(Fuca1-2)Galb1-4GlcNAcb1	35
424	Galb1-3GlcNAcb1-6(Galb1-3GlcNAcb1-2)Mana1-6(Galb1-3GlcNAcb1-2Mana1-3)M	20
425	Galb1-4GlcNAcb1-6(Fuca1-2Galb1-3GlcNAcb1-3)Galb1-4Glc-Sp21	19
426	Fuca1-3GlcNAcb1-6(Galb1-4GlcNAcb1-3)Galb1-4Glc-Sp21	19
427	GlcNAcb1-2Mana1-6(GlcNAcb1-4)(GlcNAcb1-2Mana1-3)Manb1-4GlcNAcb1-4Glc	20
428	GlcNAcb1-2Mana1-6(GlcNAcb1-4)(GlcNAcb1-4(GlcNAcb1-2)Mana1-3)Manb1-4Gl	21
429	GlcNAcb1-6(GlcNAcb1-2)Mana1-6(GlcNAcb1-4)(GlcNAcb1-2Mana1-3)Manb1-4Gl	19
430	GlcNAcb1-6(GlcNAcb1-2)Mana1-6(GlcNAcb1-4)(GlcNAcb1-4(GlcNAcb1-2)Mana1-	18
431	Galb1-4GlcNAcb1-2Mana1-6(GlcNAcb1-4)(Galb1-4GlcNAcb1-2Mana1-3)Manb1-4	16

432	Galb1-4GlcNAcb1-2Mana1-6(GlcNAcb1-4)(Galb1-4GlcNAcb1-4(Galb1-4GlcNAcb1	19
433	Galb1-4GlcNAcb1-6(Galb1-4GlcNAcb1-2)Mana1-6(GlcNAcb1-4)(Galb1-4GlcNAcb	18
434	Galb1-4GlcNAcb1-6(Galb1-4GlcNAcb1-2)Mana1-6(GlcNAcb1-4)(Galb1-4GlcNAcb	19
435	Galb1-4Galb-Sp10	18
436	Galb1-6Galb-Sp10	21
437	Neu5Aca2-3Galb1-4GlcNAcb1-3Galb-Sp8	21
438	GalNAcb1-6GalNAcb-Sp8	18
439	(6S)Galb1-3GlcNAcb-Sp0	22
440	(6S)Galb1-3(6S)GlcNAc-Sp0	18
441	Fuca1-2Galb1-4 GlcNAcb1-2Mana1-6(Fuca1-2Galb1-4GlcNAcb1-2(Fuca1-2Galb1-	22
442	Fuca1-2Galb1-4(Fuca1-3)GlcNAcb1-2Mana1-6(Fuca1-2Galb1-4(Fuca1-3)GlcNAcb	23
443	Galb1-4(Fuca1-3)GlcNAcb1-6GalNAc-Sp14	23
444	Galb1-4GlcNAcb1-2Mana-Sp0	21
445	Fuca1-2Galb1-4GlcNAcb1-6(Fuca1-2Galb1-4GlcNAcb1-3)GalNAc-Sp14	21
446	Gala1-3(Fuca1-2)Galb1-4GlcNAcb1-6(Gala1-3(Fuca1-2)Galb1-4GlcNAcb1-3)GalNA	17
447	GalNAca1-3(Fuca1-2)Galb1-4GlcNAcb1-6(GalNAca1-3(Fuca1-2)Galb1-4GlcNAcb1-	19
448	Neu5Aca2-8Neu5Aca2-3Galb1-3GalNAcb1-4(Neu5Aca2-8Neu5Aca2-3)Galb1-4Glc	23
449	GalNAcb1-4Galb1-4Glcb-Sp0	20
450	GalNAca1-3(Fuca1-2)Galb1-4GlcNAcb1-2Mana1-6(GalNAca1-3(Fuca1-2)Galb1-4G	33
451	Gala1-3(Fuca1-2)Galb1-3GlcNAcb1-2Mana1-6(Gala1-3(Fuca1-2)Galb1-3GlcNAcb1	25
452	Neu5Aca2-6Galb1-4GlcNAcb1-6(Fuca1-2Galb1-3GlcNAcb1-3)Galb1-4Glc-Sp21	17
453	GalNAca1-3(Fuca1-2)Galb1-3GlcNAcb1-2Mana1-6(GalNAca1-3(Fuca1-2)Galb1-3G	29
454	Galb1-4GlcNAcb1-6(Galb1-4GlcNAcb1-2)Mana1-6(Galb1-4GlcNAcb1-2Mana1-3)M	29
455	Neu5Aca2-3Galb1-4GlcNAcb1-2Mana1-6(GlcNAcb1-4)(Neu5Aca2-3Galb1-4GlcNA	15
456	Neu5Aca2-3Galb1-4GlcNAcb1-4Mana1-6(GlcNAcb1-4)(Neu5Aca2-3Galb1-4GlcNA	22
457	Neu5Aca2-3Galb1-4GlcNAcb1-6(Neu5Aca2-3Galb1-4GlcNAcb1-2)Mana1-6(GlcNA	20
458	Neu5Aca2-3Galb1-4GlcNAcb1-6(Neu5Aca2-3Galb1-4GlcNAcb1-2)Mana1-6(GlcNA	18
459	Neu5Aca2-6Galb1-4GlcNAcb1-2Mana1-6(GlcNAcb1-4)(Neu5Aca2-6Galb1-4GlcNA	20
460	Neu5Aca2-6Galb1-4GlcNAcb1-4Mana1-6(GlcNAcb1-4)(Neu5Aca2-6Galb1-4GlcNA	19
461	Neu5Aca2-6Galb1-4GlcNAcb1-6(Neu5Aca2-6Galb1-4GlcNAcb1-2)Mana1-6(GlcNA	20
462	Neu5Aca2-6Galb1-4GlcNAcb1-6(Neu5Aca2-6Galb1-4GlcNAcb1-2)Mana1-6(GlcNA	20
463	Gala1-3(Fuca1-2)Galb1-3GalNAca-Sp8	21
464	Gala1-3(Fuca1-2)Galb1-3GalNAcb-Sp8	21
465	Glca1-6Glca1-6Glca1-6Glcb-Sp10	23
466	Glca1-4Glca1-4Glca1-4Glcb-Sp10	94
467	Neu5Aca2-3Galb1-4GlcNAcb1-6(Neu5Aca2-3Galb1-4GlcNAcb1-3)GalNAca-Sp14	23
468	Fuca1-2Galb1-4(Fuca1-3)GlcNAcb1-2Mana1-6(Fuca1-2Galb1-4(Fuca1-3)GlcNAcb	26
469	Fuca1-2Galb1-3(Fuca1-4)GlcNAcb1-2Mana1-6(Fuca1-2Galb1-3(Fuca1-4)GlcNAcb	21
470	GlcNAcb1-6(GlcNAcb1-2)Mana1-6(GlcNAcb1-2Mana1-3)Manb1-4GlcNAcb1-4(Fu	21
471	Galb1-3GlcNAcb1-2Mana1-6(GlcNAcb1-4)(Galb1-3GlcNAcb1-2Mana1-3)Manb1-4	32
472	Neu5Aca2-6Galb1-4GlcNAcb1-6(Galb1-3GlcNAcb1-3)Galb1-4Glcb-Sp21	18
473	Neu5Aca2-3Galb1-4GlcNAcb1-2Mana-Sp0	23
474	Neu5Aca2-3Galb1-4GlcNAcb1-6GalNAca-Sp14	19
475	Neu5Aca2-6Galb1-4GlcNAcb1-6GalNAca-Sp14	21
476	Neu5Aca2-6Galb1-4 GlcNAcb1-6(Neu5Aca2-6Galb1-4GlcNAcb1-3)GalNAca-Sp14	19
477	Neu5Aca2-6Galb1-4GlcNAcb1-2Mana1-6(Neu5Aca2-6Galb1-4GlcNAcb1-2Mana1-	21
478	Neu5Aca2-3Galb1-4GlcNAcb1-2Mana1-6(Neu5Aca2-3Galb1-4GlcNAcb1-2Mana1-	22
479	Mana1-6(Mana1-3)Manb1-4GlcNAcb1-4(Fuca1-6)GlcNAcb-Sp19	19

480	Galb1-4GlcNAcb1-6(Galb1-4GlcNAcb1-2)Mana1-6(Galb1-4GlcNAcb1-2Mana1-3)M	27
481	Neu5Aca2-3Galb1-3GlcNAcb1-2Mana1-6(GlcNAcb1-4)(Neu5Aca2-3Galb1-3GlcNA	19
482	Neu5Aca2-6Galb1-4GlcNAcb1-6(Fuca1-2Galb1-4(Fuca1-3)GlcNAcb1-3)Galb1-4Gl	17
483	Galb1-3GlcNAcb1-6GalNAca-Sp14	18
484	Gala1-3Galb1-3GlcNAcb1-6GalNAca-Sp14	21
485	Galb1-3(Fuca1-4)GlcNAcb1-6GalNAca-Sp14	27
486	Neu5Aca2-3Galb1-3GlcNAcb1-6GalNAca-Sp14	20
487	(3S)Galb1-3(Fuca1-4)GlcNAcb-Sp0	19
488	Galb1-4(Fuca1-3)GlcNAcb1-6(Neu5Aca2-6(Neu5Aca2-3Galb1-3)GlcNAcb1-3)Galb	23
489	Fuca1-2Galb1-4GlcNAcb1-6GalNAca-Sp14	22
490	Gala1-3Galb1-4GlcNAcb1-6GalNAca-Sp14	19
491	Galb1-4(Fuca1-3)GlcNAcb1-2Mana-Sp0	20
492	Fuca1-2(6S)Galb1-3GlcNAcb-Sp0	17
493	Gala1-3(Fuca1-2)Galb1-4GlcNAcb1-6GalNAca-Sp14	20
494	Fuca1-2Galb1-4GlcNAcb1-2Mana-Sp0	17
495	Fuca1-2Galb1-3(6S)GlcNAcb-Sp0	23
496	Fuca1-2(6S)Galb1-3(6S)GlcNAcb-Sp0	23
497	Neu5Aca2-6GalNAcb1-4(6S)GlcNAcb-Sp8	20
498	GalNAcb1-4(Fuca1-3)(6S)GlcNAcb-Sp8	19
499	(3S)GalNAcb1-4(Fuca1-3)GlcNAcb-Sp8	19
500	Fuca1-2Galb1-3GlcNAcb1-6(Fuca1-2Galb1-3GlcNAcb1-3)GalNAca-Sp14	23
501	GalNAca1-3(Fuca1-2)Galb1-3GlcNAcb1-6GalNAca-Sp14	19
502	GlcNAcb1-6(GlcNAcb1-2)Mana1-6(GlcNAcb1-4)(GlcNAcb1-4(GlcNAcb1-2)Mana1-	15
503	Galb1-4GlcNAcb1-6(Galb1-4GlcNAcb1-2)Mana1-6(GlcNAcb1-4)Galb1-4GlcNAcb1	14
504	Galb1-3GlcNAca1-3Galb1-4GlcNAcb-Sp8	17
505	Galb1-3(6S)GlcNAcb-Sp8	15
506	(6S)(4S)GalNAcb1-4GlcNAc-Sp8	19
507	(6S)GalNAcb1-4GlcNAc-Sp8	19
508	(3S)GalNAcb1-4(3S)GlcNAc-Sp8	17
509	GalNAcb1-4(6S)GlcNAc-Sp8	17
510	(3S)GalNAcb1-4GlcNAc-Sp8	18
511	(4S)GalNAcb-Sp10	18
512	Galb1-4(6P)GlcNAcb-Sp0	26
513	(6P)Galb1-4GlcNAcb-SP0	20
514	GalNAca1-3(Fuca1-2)Galb1-4GlcNAcb1-6GalNAc-Sp14	17
515	Neu5Aca2-6Galb1-4GlcNAcb1-2Man-Sp0	21
516	Gala1-3Galb1-4GlcNAcb1-2Mana-Sp0	23
517	Gala1-3(Fuca1-2)Galb1-4GlcNAcb1-2Mana-Sp0	20
518	GalNAca1-3(Fuca1-2)Galb1-4 GlcNAcb1-2Mana-Sp0	21
519	Galb1-3GlcNAcb1-2Mana-Sp0	20
520	Gala1-3(Fuca1-2)Galb1-3GlcNAcb1-6GalNAc-Sp14	18
521	Neu5Aca2-3Galb1-3GlcNAcb1-2Mana-Sp0	19
522	Gala1-3Galb1-3GlcNAcb1-2Mana-Sp0	20
523	GalNAcb1-4GlcNAcb1-2Mana-Sp0	21
524	Neu5Aca2-3Galb1-3GalNAcb1-4Galb1-4Glcb-Sp0	20
525	GlcNAcb1-2 Mana1-6(GlcNAcb1-4)(GlcNAcb1-2Mana1-3)Manb1-4GlcNAcb1-4(Fu	19
526	Galb1-4GlcNAcb1-2 Mana1-6(GlcNAcb1-4)(Galb1-4GlcNAcb1-2Mana1-3)Manb1-4	18
527	Galb1-4GlcNAcb1-2 Mana1-6(Galb1-4GlcNAcb1-4)(Galb1-4GlcNAcb1-2Mana1-3)	21

528	Fuca1-4(Galb1-3)GlcNAcb1-2 Mana-Sp0	21
529	Neu5Aca2-3Galb1-4(Fuca1-3)GlcNAcb1-2Mana-Sp0	31
530	GlcNAcb1-3Galb1-4GlcNAcb1-6(GlcNAcb1-3)Galb1-4GlcNAc-Sp0	20
531	GalNAca1-3(Fuca1-2)Galb1-3GalNAcb1-3Gala1-4Galb1-4Glc-Sp21	27
532	Gala1-3(Fuca1-2)Galb1-3GalNAcb1-3Gala1-4Galb1-4Glc-Sp21	21
533	Galb1-3GalNAcb1-3Gal-Sp21	18
534	GlcNAcb1-3Galb1-4GlcNAcb1-2Mana1-6(GlcNAcb1-3Galb1-4GlcNAcb1-2Mana1-	17
535	GlcNAcb1-3Galb1-4GlcNAcb1-2Mana1-6(GlcNAcb1-3Galb1-4GlcNAcb1-2Mana1-	26
536	Fuca1-2Galb1-4GlcNAcb1-3Galb1-4GlcNAcb1-2Mana1-6(Fuca1-2Galb1-4GlcNAc	22
537	GlcNAcb1-3Galb1-4GlcNAcb1-3Galb1-4GlcNAcb1-2Mana1-6(GlcNAcb1-3Galb1-4	56
538	GlcNAcb1-3Galb1-4GlcNAcb1-3Galb1-4GlcNAcb1-2Mana1-6(GlcNAcb1-3Galb1-4	22
539	Galb1-4GlcNAcb1-3Galb1-4GlcNAcb1-3Galb1-4GlcNAcb1-2Mana1-6(Galb1-4Glc	81
540	Galb1-4GlcNAcb1-3Galb1-4GlcNAcb1-3Galb1-4GlcNAcb1-2Mana1-6(Galb1-4Glc	27
541	Galb1-3GlcNAcb1-3Galb1-4GlcNAcb1-2Mana1-6(Galb1-3GlcNAcb1-3Galb1-4Glc	22
542	Neu5Gca2-8Neu5Gca2-3Galb1-4GlcNAc-Sp0	18
543	Neu5Aca2-8Neu5Gca2-3Galb1-4GlcNAc-Sp0	17
544	Neu5Gca2-8Neu5Aca2-3Galb1-4GlcNAc-Sp0	16
545	Neu5Gca2-8Neu5Gca2-3Galb1-4GlcNAcb1-3Galb1-4GlcNAc-Sp0	30
546	Neu5Gca2-8Neu5Gca2-6Galb1-4GlcNAc-Sp0	18
547	Neu5Aca2-8Neu5Aca2-3Galb1-4GlcNAc-Sp0	29
548	GlcNAcb1-3Galb1-4GlcNAcb1-6(GlcNAcb1-3Galb1-4GlcNAcb1-2)Mana1-6(GlcNA	33
549	Galb1-4GlcNAcb1-3Galb1-4GlcNAcb1-6(Galb1-4GlcNAcb1-3Galb1-4GlcNAcb1-2)	44
550	Gala1-3Galb1-4GlcNAcb1-2Mana1-6(Gala1-3Galb1-4GlcNAcb1-2Mana1-3)Manb1	33
551	GlcNAcb1-3Galb1-4GlcNAcb1-6(GlcNAcb1-3Galb1-3)GalNAca-Sp14	16
552	GalNAcb1-3GlcNAcb-Sp0	18
553	GalNAcb1-4GlcNAcb1-3GalNAcb1-4GlcNAcb-Sp0	18
554	GlcNAcb1-3Galb1-4GlcNAcb1-3Galb1-4GlcNAcb1-3Galb1-4GlcNAcb1-3Galb1-4G	26
555	Galb1-4GlcNAcb1-3Galb1-4GlcNAcb1-3Galb1-4GlcNAcb1-3Galb1-4GlcNAcb1-3G	23
556	GlcNAb1-3Galb1-3GalNAc-Sp14	25
557	Galb1-3GlcNAcb1-6(Galb1-3)GalNAc-Sp14	21
558	Galb1-4GlcNAcb1-3Galb1-4GlcNAcb1-3Galb1-4GlcNAcb1-3Galb1-4GlcNAcb1-3G	16
559	(3S)GlcAb1-3Galb1-4GlcNAcb1-3Galb1-4Glc-Sp0	17
560	(3S)GlcAb1-3Galb1-4GlcNAcb1-2Mana-Sp0	18
561	Galb1-3GlcNAcb1-3Galb1-4GlcNAcb1-3Galb1-4GlcNAcb1-6(Galb1-3GlcNAcb1-3	29
562	Galb1-3GlcNAcb1-3Galb1-4GlcNAcb1-6(Galb1-3GlcNAcb1-3Galb1-4GlcNAb1-2)M	29
563	Neu5Aca2-8Neu5Aca2-3Galb1-3GalNAcb1-4(Neu5Aca2-3)Galb1-4Glc-Sp21	17
564	GlcNAcb1-3Galb1-4GlcNAcb1-2Mana1-6(GlcNAcb1-3Galb1-4GlcNAcb1-2Mana1-	20
565	Galb1-4GlcNAcb1-3Galb1-4GlcNAcb1-2Mana1-6(Galb1-4GlcNAcb1-3Galb1-4Glc	18
566	GlcNAcb1-3Galb1-4GlcNAcb1-3Galb1-4GlcNAcb1-2Mana1-6(GlcNAcb1-3Galb1-4	22
567	Galb1-4GlcNAcb1-3Galb1-4GlcNAcb1-3Galb1-4GlcNAcb1-2Mana1-6(Galb1-4Glc	15
568	GlcNAcb1-3Galb1-4GlcNAcb1-3Galb1-4GlcNAcb1-3Galb1-4GlcNAcb1-2Mana1-6	17
569	Galb1-4GlcNAcb1-3Galb1-4GlcNAcb1-3Galb1-4GlcNAcb1-3Galb1-4GlcNAcb1-2M	20
570	GlcNAcb1-3Galb1-4GlcNAcb1-3Galb1-4GlcNAcb1-3Galb1-4GlcNAcb1-3Galb1-4G	15
571	Galb1-4GlcNAcb1-3Galb1-4GlcNAcb1-3Galb1-4GlcNAcb1-3Galb1-4GlcNAcb1-3G	18
572	Galb1-4GlcNAcb1-3Galb1-4GlcNAcb1-6(Galb1-4GlcNAcb1-3Galb1-4GlcNAb1-2)M	17
573	GlcNAcb1-3Galb1-4GlcNAcb1-3Galb1-4GlcNAcb1-6(GlcNAcb1-3Galb1-4GlcNAcb	20
574	Galb1-4GlcNAcb1-3Galb1-4GlcNAcb1-3Galb1-4GlcNAcb1-6(Galb1-4GlcNAcb1-3	24
575	GlcNAcb1-3Galb1-4GlcNAcb1-3Galb1-4GlcNAcb1-3Galb1-4GlcNAcb1-6(GlcNAcb	16

576	Galb1-4GlcNAcb1-3Galb1-4GlcNAcb1-3Galb1-4GlcNAcb1-3Galb1-4GlcNAcb1-6(	21
577	GlcNAcb1-3Galb1-4GlcNAcb1-3Galb1-4GlcNAcb1-3Galb1-4GlcNAcb1-3Galb1-4G	17
578	Galb1-4GlcNAcb1-3Galb1-4GlcNAcb1-3Galb1-4GlcNAcb1-3Galb1-4GlcNAcb1-3G	23
579	Galb1-4GlcNAcb1-3Galb1-4GlcNAcb1-3GalNAca-Sp14	29
580	Galb1-4GlcNAcb1-3Galb1-4GlcNAcb1-6(Galb1-3)GalNAca-Sp14	27
581	Galb1-4GlcNAcb1-3Galb1-4GlcNAcb1-6(Galb1-4GlcNAcb1-3Galb1-4GlcNAcb1-3)	25
582	Neu5Aca2-3Galb1-4GlcNAcb1-3Galb1-4GlcNAcb1-3GalNAca-Sp14	16
583	GlcNAcb1-3Galb1-4GlcNAcb1-3GalNAca-Sp14	18
584	GlcNAcb1-3Galb1-4GlcNAcb1-6(Galb1-3)GalNAca-Sp14	19
585	GlcNAcb1-3Galb1-4GlcNAcb1-6(GlcNAcb1-3Galb1-4GlcNAcb1-3)GalNAca-Sp14	18
586	Neu5Aca2-3Galb1-4GlcNAcb1-3Galb1-4GlcNAcb1-6(Neu5Aca2-3Galb1-4GlcNAcb	19
587	Neu5Aca2-6Galb1-4GlcNAcb1-3Galb1-4GlcNAcb1-3GalNAca-Sp14	25
588	GlcNAcb1-3Galb1-4GlcNAcb1-3Galb1-4GlcNAcb1-3GalNAca-Sp14	19
589	Galb1-4GlcNAcb1-3Galb1-3GalNAca-Sp14	27
590	Neu5Aca2-3Galb1-4GlcNAcb1-3Galb1-4GlcNAcb1-6(Galb1-3)GalNAca-Sp14	19
591	Neu5Aca2-6Galb1-4GlcNAcb1-3Galb1-4GlcNAcb1-6(Galb1-3)GalNAca-Sp14	16
592	Neu5Aca2-6Galb1-4GlcNAcb1-6(Galb1-3)GalNAca-Sp14	28
593	Neu5Aca2-3Galb1-4GlcNAcb1-3Galb1-4GlcNAcb1-2Mana1-6(Neu5Aca2-3Galb1-4	17
594	GlcNAcb1-6(Neu5Aca2-3Galb1-3)GalNAca-Sp14	19
595	Neu5Aca2-6Galb1-4GlcNAcb1-3Galb1-4GlcNAcb1-6(Neu5Aca2-6Galb1-4GlcNAcb	17
596	Neu5Aca2-6Galb1-4GlcNAcb1-3Galb1-4GlcNAcb1-3Galb1-4GlcNAcb1-2Mana1-6	47
597	Neu5Aca2-3Galb1-4GlcNAcb1-3Galb1-4GlcNAcb1-3Galb1-4GlcNAcb1-2Mana1-6	27
598	Neu5Aca2-6Galb1-4GlcNAcb1-3Galb1-4GlcNAcb1-2Mana1-6(Neu5Aca2-6Galb1-4	22
599	GlcNAcb1-3Fuca-Sp21	18
600	Galb1-3GalNAcb1-4(Neu5Aca2-8Neu5Aca2-8Neu5Aca2-3)Galb1-4Glcb-Sp21	20

STDEV	%CV
1	8
8	33
3	15
3	20
1	4
4	23
2	8
3	15
1	5
3	16
1	7
2	10
4	24
1	9
4	21
4	21
2	11
3	14
2	13
1	6
4	23
1	5
5	23
1	3
1	6
5	30
3	14
3	15
2	9
1	3
3	19
2	12
3	19
1	7
1	5
3	14
2	11
3	19
3	18
9	42
2	11
2	11
3	15
2	8
2	12
5	26
2	8

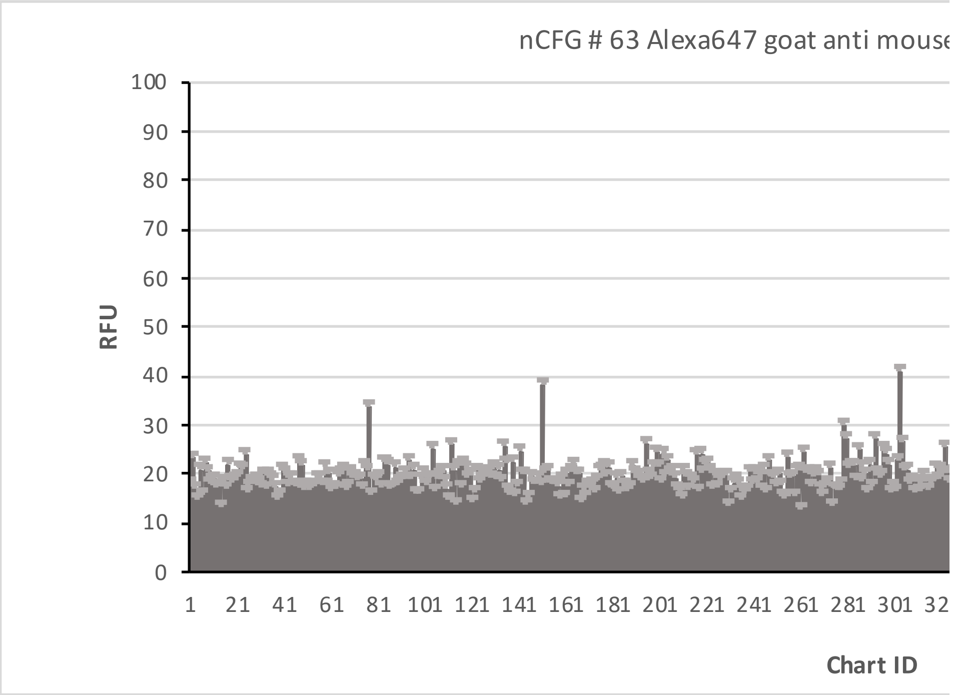

3	14
3	16
3	14
2	13
3	18
2	10
4	22
2	11
3	13
2	10
2	11
2	8
5	24
2	14
2	9
1	5
3	18
3	15
6	31
2	12
1	3
3	15
3	17
4	21
2	11
4	19
3	16
2	7
2	9
19	56
3	21
1	7
2	11
2	11
1	7
4	19
1	6
12	55
2	14
2	10
4	18
2	10
4	24
2	11
1	6
1	7
4	17
3	14

3	13
2	13
1	5
3	14
3	13
4	22
2	8
4	22
4	16
1	6
2	13
1	3
3	15
2	9
2	13
2	15
10	38
1	6
1	4
4	20
3	17
4	16
4	16
1	7
5	23
3	22
1	8
4	17
4	23
3	15
5	26
3	15
2	8
1	7
3	12
3	17
2	9
3	14
5	20
12	55
1	6
2	13
1	4
3	19
4	20
2	8
1	7
2	9

2	11
3	22
3	13
2	12
3	16
5	26
1	5
2	6
3	14
1	5
3	16
3	16
1	8
6	31
2	12
3	18
2	11
1	4
2	14
3	13
4	16
2	11
2	12
1	9
2	13
2	10
3	16
3	16
1	5
1	3
2	12
1	2
2	8
2	10
3	13
2	10
2	10
2	14
3	16
2	10
2	11
1	7
3	15
1	8
3	20
7	30
2	8
1	5

2	10
3	17
1	3
7	28
3	14
3	14
5	24
3	12
2	11
3	14
1	4
1	3
4	17
1	6
3	16
2	12
2	14
1	6
1	8
3	13
3	16
1	6
2	13
2	12
5	20
2	12
6	24
3	15
3	16
3	15
2	11
2	10
2	9
2	13
3	16
3	15
2	10
4	19
1	7
2	9
4	19
3	14
2	10
1	6
3	20
3	18
3	17
3	15

2	14
4	22
3	15
4	18
2	9
2	10
2	13
4	17
2	12
2	9
8	43
2	11
4	24
3	21
2	16
3	13
2	11
2	12
2	12
2	11
3	14
2	16
5	19
2	11
3	15
2	10
3	13
3	12
2	10
2	10
2	11
1	6
4	22
3	16
2	13
5	29
2	10
3	17
2	12
5	16
4	15
2	10
3	15
4	18
1	7
1	6
1	5
1	6

1	7
3	20
4	19
1	6
8	29
3	14
3	14
2	9
4	17
4	15
1	6
1	6
3	15
1	8
5	23
15	38
3	13
2	10
3	16
1	6
2	8
4	22
2	11
2	10
4	21
2	8
3	14
3	16
1	5
2	9
4	20
3	14
3	14
4	19
3	11
2	11
2	12
2	8
4	19
2	13
1	5
2	8
4	20
2	12
1	4
3	16
4	13
1	3

4	28
3	13
3	18
3	12
1	7
4	17
3	14
3	17
1	5
22	75
2	12
3	11
1	7
1	5
2	7
3	14
4	25
2	12
4	18
2	11
3	21
7	25
2	8
5	24
2	8
5	27
3	15
2	13
2	10
2	8
3	17
1	8
2	11
2	14
5	28
1	6
5	23
5	25
2	5
2	8
2	10
2	9
4	24
1	9
3	15
1	8
3	16
4	18

3	13
2	10
3	16
1	7
8	33
9	13
5	21
3	13
3	18
4	21
4	21
4	21
2	8
2	10
2	11
4	19
4	21
1	7
2	12
3	16
4	19
3	17
3	12
3	13
3	17
4	21
3	14
2	11
6	24
4	18
4	14
4	22
4	15
21	44
2	10
3	14
6	25
2	11
2	6
2	6
1	6
1	7
4	21
2	9
1	6
2	11
3	14
2	11

2	12
2	13
3	14
2	12
3	13
4	21
2	8
1	6
4	20
3	13
2	9
1	6
4	17
2	10
1	6
1	7
2	9
4	21
3	8
2	9
1	3
5	18
11	39
1	6
4	20
3	14
1	7
3	17
1	5
1	5
3	13
2	10
2	8
3	12
4	4
3	12
4	15
3	12
2	10
5	15
2	12
2	8
4	21
3	15
3	15
4	19
4	19
1	5

2	9
2	13
2	9
6	32
2	8
3	12
2	7
2	9
2	10
1	4
3	17
1	4
2	9
3	16
3	15
2	11
2	10
2	12
1	7
3	16
3	11
1	6
2	12
1	6
3	20
2	16
3	15
2	10
1	8
2	9
2	14
2	11
2	8
2	9
2	12
4	17
2	8
3	13
2	10
2	7
3	19
3	18
3	14
4	21
3	16
4	23
2	14
4	19

3	12
6	20
3	16
2	6
3	13
2	10
1	6
2	9
2	9
3	5
2	8
15	19
1	5
3	14
2	8
3	14
2	9
5	16
3	16
8	29
5	15
2	6
3	10
3	20
2	12
2	11
3	12
3	11
5	21
4	21
1	5
3	17
1	5
2	6
4	14
2	14
4	20
3	14
2	9
3	22
2	9
2	9
3	16
5	26
1	8
3	15
2	6
2	11

2	8
3	16
5	21
2	8
3	12
3	13
2	13
3	19
2	11
2	9
3	14
4	17
3	15
3	12
2	9
3	18
10	37
1	6
1	5
2	10
2	5
1	3
3	12
1	3
1	3

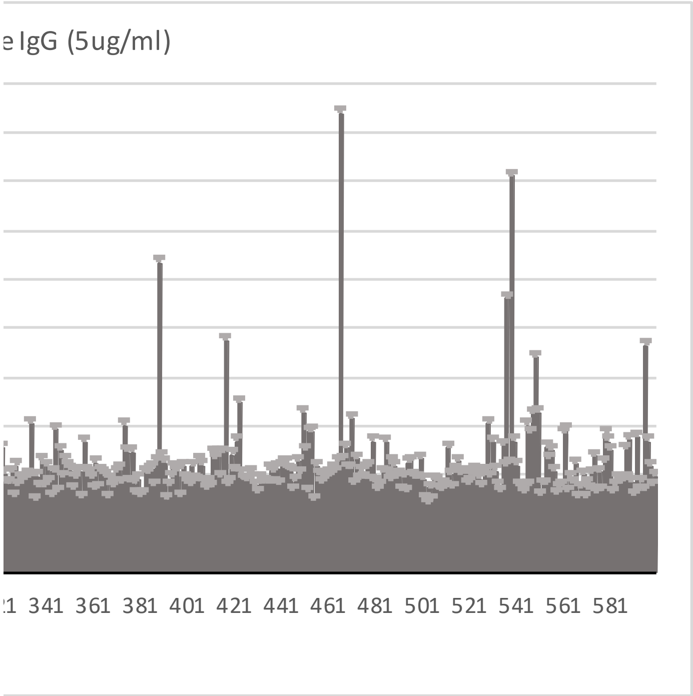

ChartID
466
539
389
537
417
596
549
303
151
423
77
450
550
548
471
334
529
374
545
279
345
562
454
453
547
561
579
592
280
292
589
422
480
540
580
597
357
485
531
304
195
112
535
134
554
322
468

512
104
296
587
285
347
451
556
581
141
375
377
199
262
412
414
416
202
218
297
420
24
216
390
574
255
2
413
473
500
555
339
349
406
467
47
94
204
247
302
388
448
465
488
496
516
83
84

138
362
391
442
443
495
578
7
117
135
221
538
566
17
85
164
220
290
298
328
407
541
48
75
115
177
189
282
283
341
350
399
403
441
456
478
489
536
598
58
88
130
179
198
200
287
386
395

439
22
118
273
281
318
372
436
463
469
470
477
5
23
40
66
96
132
176
245
260
306
320
351
385
444
515
523
532
76
108
113
123
153
163
175
206
211
222
259
305
355
358
364
396
397
421
445

475
527
528
557
8
69
128
239
241
264
267
326
344
354
371
383
428
437
464
484
518
576
41
65
92
100
152
190
205
219
263
293
323
359
365
384
424
427
449
461
486
519
573
32
34
60
120
127

142
143
161
165
166
196
251
288
333
457
462
491
513
517
522
524
569
107
126
191
224
228
229
268
295
313
321
335
337
366
497
600
21
64
71
81
146
178
184
203
212
227
243
248
257
266
348
394

415
459
493
530
564
35
43
44
55
56
70
102
103
119
129
149
182
207
249
256
294
330
331
353
360
380
400
429
432
460
476
490
498
499
525
584
588
11
20
29
73
79
91
93
95
99
106
125

131
159
197
214
233
319
332
368
404
425
474
501
506
10
18
36
72
80
174
192
193
226
284
308
405
426
447
479
487
507
586
15
27
90
109
133
286
310
314
317
342
376
410
419
434
481
521
590

594
19
28
30
124
154
194
201
215
232
242
272
316
324
325
370
373
378
401
408
433
435
438
458
565
571
585
1
9
147
148
155
156
172
234
240
278
307
312
440
472
510
520
552
553
45
50
52

57
59
89
101
150
170
185
186
252
329
387
393
402
418
430
483
533
542
546
560
583
599
13
26
42
46
53
54
68
140
157
162
250
265
291
300
338
340
369
411
511
526
12
31
37
62
82
87

116
171
180
188
225
271
363
504
514
543
559
568
3
16
33
63
74
86
110
181
208
223
269
275
352
361
409
508
572
49
51
67
136
168
213
236
238
244
276
277
315
446
482
492
509
577
593
595

61
105
187
217
301
311
346
452
494
534
563
25
122
173
209
231
246
289
299
309
431
544
575
582
97
98
183
237
379
382
558
6
39
78
137
551
591
139
253
258
270
327
343
381
398
145
160
169

356
367
567
570
158
210
254
392
502
4
38
111
235
336
455
505
121
167
503
114
144
230
274
14
261

Glycan
Glca1-4Glca1-4Glca1-4Glcb-Sp10
Galb1-4GlcNAcb1-3Galb1-4GlcNAcb1-3Galb1-4GlcNAcb1-2Mana1-6(Galb1-4GlcNAcb1-3Galb1-4GlcNAcb1-3Galb1-4GlcN
GalNAca1-3(Fuca1-2)Galb1-3GalNAca1-3(Fuca1-2)Galb1-4GlcNAcb-Sp0
GlcNAcb1-3Galb1-4GlcNAcb1-3Galb1-4GlcNAcb1-2Mana1-6(GlcNAcb1-3Galb1-4GlcNAcb1-3Galb1-4GlcNAcb1-2Mana1-3
GlcNAcb1-2(GlcNAcb1-6)Mana1-6(GlcNAcb1-2Mana1-3)Manb1-4GlcNAcb1-4GlcNAcb-Sp19
Neu5Aca2-6Galb1-4GlcNAcb1-3Galb1-4GlcNAcb1-3Galb1-4GlcNAcb1-2Mana1-6(Neu5Aca2-6Galb1-4GlcNAcb1-3Galb1-4
Galb1-4GlcNAcb1-3Galb1-4GlcNAcb1-6(Galb1-4GlcNAcb1-3Galb1-4GlcNAcb1-2)Mana1-6(Galb1-4GlcNAcb1-3Galb1-4Glc
Galb1-4GlcNAcb1-6Galb1-4GlcNAcb-Sp0
Galb1-4(Fuca1-3)GlcNAcb-Sp0
Gala1-3(Fuca1-2)Galb1-4GlcNAcb1-2Mana1-6(Gala1-3(Fuca1-2)Galb1-4GlcNAcb1-2Mana1-3)Manb1-4GlcNAcb1-4(Fuca1-
Fuca1-2Galb1-4Glcb-Sp0
GalNAca1-3(Fuca1-2)Galb1-4GlcNAcb1-2Mana1-6(GalNAca1-3(Fuca1-2)Galb1-4GlcNAcb1-2Mana1-3)Manb1-4GlcNAcb1-4
Gala1-3Galb1-4GlcNAcb1-2Mana1-6(Gala1-3Galb1-4GlcNAcb1-2Mana1-3)Manb1-4GlcNAcb1-4GlcNAc-Sp24
GlcNAcb1-3Galb1-4GlcNAcb1-6(GlcNAcb1-3Galb1-4GlcNAcb1-2)Mana1-6(GlcNAcb1-3Galb1-4GlcNAcb1-2Man a1-3)Manb
Galb1-3GlcNAcb1-2Mana1-6(GlcNAcb1-4)(Galb1-3GlcNAcb1-2Mana1-3)Manb1-4GlcNAcb1-4GlcNAcb-Sp21
Neu5Aca2-3Galb1-4(Fuca1-3)GlcNAcb1-6(Neu5Aca2-3Galb1-3)GalNAc-Sp14
Neu5Aca2-3Galb1-4(Fuca1-3)GlcNAcb1-2Mana-Sp0
Neu5Aca2-3Galb1-4(Fuca1-3)GlcNAcb1-3GalNAca-Sp14
Neu5Gca2-8Neu5Gca2-3Galb1-4GlcNAcb1-3Galb1-4GlcNAc-Sp0
Neu5Gca2-3Galb1-3GlcNAcb-Sp0
Neu5Aca2-6Galb1-4GlcNAcb1-2Mana1-3Manb1-4GlcNAcb1-4GlcNAc-Sp12
Galb1-3GlcNAcb1-3Galb1-4GlcNAcb1-6(Galb1-3GlcNAcb1-3Galb1-4GlcNAb1-2)Mana1-6(Galb1-3GlcNAcb1-3Galb1-4GlcN
Galb1-4GlcNAcb1-6(Galb1-4GlcNAcb1-2)Mana1-6(Galb1-4GlcNAcb1-2Mana1-3)Manb1-4GlcNAcb1-4GlcNAcb-Sp19
GalNAca1-3(Fuca1-2)Galb1-3GlcNAcb1-2Mana1-6(GalNAca1-3(Fuca1-2)Galb1-3GlcNAcb1-2Mana1-3)Manb1-4GlcNAcb1-4
Neu5Aca2-8Neu5Aca2-3Galb1-4GlcNAc-Sp0
Galb1-3GlcNAcb1-3Galb1-4GlcNAcb1-3Galb1-4GlcNAcb1-6(Galb1-3GlcNAcb1-3Galb1-4GlcNAcb1-3Galb1-4GlcNAb1-2)M
Galb1-4GlcNAcb1-3Galb1-4GlcNAcb1-3GalNAca-Sp14
Neu5Aca2-6Galb1-4GlcNAcb1-6(Galb1-3)GalNAca-Sp14
Neu5Gca2-3Galb1-4(Fuca1-3)GlcNAcb-Sp0
Neu5Aca2-3Galb1-3GlcNAcb1-3Galb1-3GlcNAcb-Sp0
Galb1-4GlcNAcb1-3Galb1-3GalNAca-Sp14
Fuca1-2Galb1-3GlcNAcb1-2Mana1-6(Fuca1-2Galb1-3GlcNAcb1-2Mana1-3)Manb1-4GlcNAcb1-4(Fuca1-6)GlcNAcb-Sp22
Galb1-4GlcNAcb1-6(Galb1-4GlcNAcb1-2)Mana1-6(Galb1-4GlcNAcb1-2Mana1-3)Manb1-4GlcNAcb1-4(Fuca1-6)GlcNAcb-Sp
Galb1-4GlcNAcb1-3Galb1-4GlcNAcb1-3Galb1-4GlcNAcb1-2Mana1-6(Galb1-4GlcNAcb1-3Galb1-4GlcNAcb1-3Galb1-4GlcN
Galb1-4GlcNAcb1-3Galb1-4GlcNAcb1-6(Galb1-3)GalNAca-Sp14
Neu5Aca2-3Galb1-4GlcNAcb1-3Galb1-4GlcNAcb1-3Galb1-4GlcNAcb1-2Mana1-6(Neu5Aca2-3Galb1-4GlcNAcb1-3Galb1-4
Fuca1-2Galb1-3GlcNAcb1-2Mana1-6(Fuca1-2Galb1-3GlcNAcb1-2Mana1-3)Manb1-4GlcNAcb1-4GlcNAcb-Sp20
Galb1-3(Fuca1-4)GlcNAcb1-6GalNAca-Sp14
GalNAca1-3(Fuca1-2)Galb1-3GalNAcb1-3Gala1-4Galb1-4Glc-Sp21
GalNAcb1-3Galb-Sp8
Glca1-4Glcb-Sp8
Gala1-3GalNAcb-Sp8
GlcNAcb1-3Galb1-4GlcNAcb1-2Mana1-6(GlcNAcb1-3Galb1-4GlcNAcb1-2Mana1-3)Manb1-4GlcNAcb1-4GlcNAcb-Sp25
GlcNAcb1-6(Galb1-3)GalNAca-Sp14
GlcNAcb1-3Galb1-4GlcNAcb1-3Galb1-4GlcNAcb1-3Galb1-4GlcNAcb1-3Galb1-4GlcNAcb1-2Mana1-6(GlcNAcb1-3Galb1-4
Neu5Aca2-3Galb1-4GlcNAcb1-2Mana1-6(Neu5Aca2-3Galb1-4GlcNAcb1-2Mana1-3)Manb1-4GlcNAcb1-4GlcNAcb-Sp12
Fuca1-2Galb1-4(Fuca1-3)GlcNAcb1-2Mana1-6(Fuca1-2Galb1-4(Fuca1-3)GlcNAcb1-2Mana1-3)Manb1-4GlcNAcb1-4(Fuca1-

Galb1-4(6P)GlcNAcb-Sp0
Gala1-3(Fuca1-2)Galb1-4(Fuca1-3)GlcNAcb-Sp8
(6P)Glcb-Sp10
Neu5Aca2-6Galb1-4GlcNAcb1-3Galb1-4GlcNAcb1-3GalNAca-Sp14
Neu5Gca-Sp8
Galb1-4GlcNAcb1-2Mana1-6Manb1-4GlcNAcb1-4GlcNAc-Sp12
Gala1-3(Fuca1-2)Galb1-3GlcNAcb1-2Mana1-6(Gala1-3(Fuca1-2)Galb1-3GlcNAcb1-2Mana1-3)Manb1-4GlcNAcb1-4(Fuca1-
GlcNAb1-3Galb1-3GalNAc-Sp14
Galb1-4GlcNAcb1-3Galb1-4GlcNAcb1-6(Galb1-4GlcNAcb1-3Galb1-4GlcNAcb1-3)GalNAca-Sp14
Galb1-3GalNAca-Sp16
GalNAcb1-4GlcNAcb1-2Mana1-6(GalNAcb1-4GlcNAcb1-2Mana1-3)Manb1-4GlcNAcb1-4GlcNAc-Sp12
Galb1-3GalNAca1-3(Fuca1-2)Galb1-4GlcNAc-Sp0
Glcb1-6Glcb-Sp8
Neu5Aca2-3Galb1-4Glcb-Sp8
Fuca1-2Galb1-4(Fuca1-3)GlcNAcb1-3GalNAca-Sp14
GalNAca1-3(Fuca1-2)Galb1-4(Fuca1-3)GlcNAcb1-3GalNAc-Sp14
Fuca1-2Galb1-4GlcNAcb1-2Mana1-6(Fuca1-2Galb1-4GlcNAcb1-2Mana1-3)Manb1-4GlcNAcb1-4(Fuca1-6)GlcNAcb-Sp22
GlcAb-Sp8
Neu5Aca2-3Galb1-4GlcNAcb1-3Galb1-4(Fuca1-3)GlcNAcb-Sp0
Neu5Aca2-3Galb1-4(Fuca1-3)GlcNAcb1-6(Galb1-3)GalNAca-Sp14
GalNAca1-3(Fuca1-2)Galb1-3GlcNAcb1-3GalNAc-Sp14
(3S)Galb1-4(Fuca1-3)(6S)Glc-Sp0
Mana1-6(Mana1-3)Mana1-6(Mana1-3)Manb1-4GlcNAcb1-4GlcNAcb-Sp12
Gala1-3Galb1-3GlcNAcb1-2Mana1-6(Gala1-3Galb1-3GlcNAcb1-2Mana1-3)Manb1-4GlcNAcb1-4GlcNAc-Sp19
Galb1-4GlcNAcb1-3Galb1-4GlcNAcb1-3Galb1-4GlcNAcb1-6(Galb1-4GlcNAcb1-3Galb1-4GlcNAcb1-3Galb1-4GlcNAb1-2)M
Neu5Aca2-3Galb1-4(Fuca1-3)GlcNAcb1-3Galb1-4GlcNAcb-Sp8
Glca-Sp8
Gala1-3(Fuca1-2)Galb1-4(Fuca1-3)GlcNAcb1-3GalNAc-Sp14
Neu5Aca2-3Galb1-4GlcNAcb1-2Mana-Sp0
Fuca1-2Galb1-3GlcNAcb1-6(Fuca1-2Galb1-3GlcNAcb1-3)GalNAca-Sp14
Galb1-4GlcNAcb1-3Galb1-4GlcNAcb1-3Galb1-4GlcNAcb1-3Galb1-4GlcNAcb1-3Galb1-4GlcNAcb1-2Mana1-6(Galb1-4GlcN
GlcNAca1-4Galb1-4GlcNAcb1-3Galb1-4(Fuca1-3)GlcNAcb1-3Galb1-4(Fuca1-3)GlcNAcb-Sp0
GlcNAcb1-2Mana1-6(GlcNAcb1-2Mana1-3)Manb1-4GlcNAcb1-4(Fuca1-6)GlcNAcb-Sp22
Galb1-4GlcNAcb1-6(Neu5Aca2-6Galb1-3GlcNAcb1-3)Galb1-4Glc-Sp21
Neu5Aca2-3Galb1-4GlcNAcb1-6(Neu5Aca2-3Galb1-4GlcNAcb1-3)GalNAca-Sp14
(6S)GlcNAcb-Sp8
GalNAcb1-3GalNAca-Sp8
GlcAb1-6Galb-Sp8
Fuca1-2(6S)Galb1-4Glcb-Sp0
Galb1-4GlcNAca1-6Galb1-4GlcNAcb-Sp0
GalNAcb1-4(Neu5Aca2-3)Galb1-4GlcNAcb1-3GalNAca-Sp14
Neu5Aca2-8Neu5Aca2-3Galb1-3GalNAcb1-4(Neu5Aca2-8Neu5Aca2-3)Galb1-4Glcb-Sp0
Glca1-6Glca1-6Glca1-6Glcb-Sp10
Galb1-4(Fuca1-3)GlcNAcb1-6(Neu5Aca2-6(Neu5Aca2-3Galb1-3)GlcNAcb1-3)Galb1-4Glc-Sp21
Fuca1-2(6S)Galb1-3(6S)GlcNAcb-Sp0
Gala1-3Galb1-4GlcNAcb1-2Mana-Sp0
GalNAca1-3(Fuca1-2)Galb1-4(Fuca1-3)GlcNAcb-Sp0
(3S)Galb1-4(Fuca1-3)Glcb-Sp0

Neu5Aca2-6(Galb1-3)GlcNAcb1-4Galb1-4Glcb-Sp10
Fuca1-4(Galb1-3)GlcNAcb1-2Mana1-6(Fuca1-4(Galb1-3)GlcNAcb1-2Mana1-3)Manb1-4GlcNAcb1-4(Fuca1-6)GlcNAcb-Sp2
Gala1-3Galb1-3(Fuca1-4)GlcNAcb1-2Mana1-6(Gala1-3Galb1-3(Fuca1-4)GlcNAcb1-2Mana1-3)Manb1-4GlcNAcb1-4GlcNAc
Fuca1-2Galb1-4(Fuca1-3)GlcNAcb1-2Mana1-6(Fuca1-2Galb1-4(Fuca1-3)GlcNAcb1-4(Fuca1-2Galb1-4(Fuca1-3)GlcNAcb1-2
Galb1-4(Fuca1-3)GlcNAcb1-6GalNAc-Sp14
Fuca1-2Galb1-3(6S)GlcNAcb-Sp0
Galb1-4GlcNAcb1-3Galb1-4GlcNAcb1-3Galb1-4GlcNAcb1-3Galb1-4GlcNAcb1-3Galb1-4GlcNAcb1-6(Galb1-4GlcNAcb1-3G
Fuca-Sp9
Gala1-3Galb1-4Glc-Sp10
Neu5Aca2-6(Galb1-3)GalNAca-Sp8
Fuca1-2Galb1-4(6S)GlcNAcb-Sp8
GlcNAcb1-3Galb1-4GlcNAcb1-3Galb1-4GlcNAcb1-2Mana1-6(GlcNAcb1-3Galb1-4GlcNAcb1-3Galb1-4GlcNAcb1-2Mana1-
GlcNAcb1-3Galb1-4GlcNAcb1-3Galb1-4GlcNAcb1-2Mana1-6(GlcNAcb1-3Galb1-4GlcNAcb1-3Galb1-4GlcNAcb1-2Mana1-
GlcNAcb-Sp8
GalNAca1-3(Fuca1-2)Galb1-4GlcNAcb-Sp0
Galb1-4GlcNAcb1-3Galb1-4Glcb-Sp0
Fuca1-2(6S)Galb1-4GlcNAcb-Sp0
Galb1-4(Fuca1-3)GlcNAcb1-3Galb1-3(Fuca1-4)GlcNAcb-Sp0
Galb1-3Galb1-4GlcNAcb-Sp8
Neu5Aca2-3Galb1-3(Fuca1-4)GlcNAcb1-3Galb1-3(Fuca1-4)GlcNAcb-Sp0
Galb1-3GalNAcb1-4(Neu5Aca2-8Neu5Aca2-3)Galb1-4Glcb-Sp0
Galb1-3GlcNAcb1-3Galb1-4GlcNAcb1-2Mana1-6(Galb1-3GlcNAcb1-3Galb1-4GlcNAcb1-2Mana1-3)Manb1-4GlcNAcb1-4G
Neu5,9Ac2a-Sp8
Fuca1-2Galb1-4GlcNAcb-Sp0
Gala1-3Galb1-4GlcNAcb-Sp8
GlcNAcb1-6(GlcNAcb1-3)GalNAca-Sp14
GlcNAcb1-4GlcNAcb1-4GlcNAcb1-4GlcNAcb1-4GlcNAcb1-4GlcNAcb1-Sp8
Neu5Gca2-3Galb1-4Glcb-Sp0
Neu5Gca2-6GalNAca-Sp0
GlcNAca1-4Galb1-3GalNAc-Sp14
Galb1-4GlcNAcb1-2Mana1-6(Galb1-4GlcNAcb1-2Mana1-3)Manb1-4GlcNAcb1-4(Fuca1-6)GlcNAcb-Sp22
Gala1-4Galb1-3GlcNAcb1-2Mana1-6(Gala1-4Galb1-3GlcNAcb1-2Mana1-3)Manb1-4GlcNAcb1-4GlcNAcb-Sp19
Galb1-3GlcNAca1-6Galb1-4GlcNAcb-Sp0
Fuca1-2Galb1-4 GlcNAcb1-2Mana1-6(Fuca1-2Galb1-4GlcNAcb1-2(Fuca1-2Galb1-4GlcNAcb1-4)Mana1-3)Manb1-4GlcNAc
Neu5Aca2-3Galb1-4GlcNAcb1-4Mana1-6(GlcNAcb1-4)(Neu5Aca2-3Galb1-4GlcNAcb1-4(Neu5Aca2-3Galb1-4GlcNAcb1-2)M
Neu5Aca2-3Galb1-4GlcNAcb1-2Mana1-6(Neu5Aca2-3Galb1-4GlcNAcb1-2Mana1-3)Manb1-4GlcNAcb1-4(Fuca1-6)GlcNAc
Fuca1-2Galb1-4GlcNAcb1-6GalNAca-Sp14
Fuca1-2Galb1-4GlcNAcb1-3Galb1-4GlcNAcb1-2Mana1-6(Fuca1-2Galb1-4GlcNAcb1-3Galb1-4GlcNAcb1-2Mana1-3)Manb1
Neu5Aca2-6Galb1-4GlcNAcb1-3Galb1-4GlcNAcb1-2Mana1-6(Neu5Aca2-6Galb1-4GlcNAcb1-3Galb1-4GlcNAcb1-2Mana1-
Fuca1-2Galb1-3GalNAcb1-3Gala-Sp9
GlcNAcb1-3Galb1-3GalNAca-Sp8
Fuca1-4(Galb1-3)GlcNAcb-Sp8
GlcNAcb1-3GalNAca-Sp8
Glcb1-4Glcb-Sp8
G-ol-Sp8
Galb1-3GlcNAcb1-3Galb1-3GlcNAcb-Sp0
Fuca1-2Galb1-3GalNAca1-3(Fuca1-2)Galb1-4GlcNAcb-Sp0
Neu5Aca2-3Galb1-3GlcNAcb1-3GalNAca-Sp14

(6S)Galb1-3GlcNAcb-Sp0
6S(3S)Galb1-4(6S)GlcNAcb-Sp0
Gala1-3Galb-Sp8
Neu5Aca2-8Neu5Aca-Sp8
Neu5Gca2-3Galb1-4GlcNAcb-Sp0
Neu5Aca2-8Neu5Acb-Sp17
Neu5Aca2-3Galb1-4GlcNAcb1-3GalNAc-Sp14
Galb1-6Galb-Sp10
Gala1-3(Fuca1-2)Galb1-3GalNAca-Sp8
Fuca1-2Galb1-3(Fuca1-4)GlcNAcb1-2Mana1-6(Fuca1-2Galb1-3(Fuca1-4)GlcNAcb1-2Mana1-3)Manb1-4GlcNAcb1-4(Fuca1-
GlcNAcb1-6(GlcNAcb1-2)Mana1-6(GlcNAcb1-2Mana1-3)Manb1-4GlcNAcb1-4(Fuca1-6)GlcNAcb-Sp24
Neu5Aca2-6Galb1-4GlcNAcb1-2Mana1-6(Neu5Aca2-6Galb1-4GlcNAcb1-2Mana1-3)Manb1-4GlcNAcb1-4(Fuca1-6)GlcNAcb
GalNAca-Sp15
6S(3S)Galb1-4GlcNAcb-Sp0
(4S)Galb1-4GlcNAcb-Sp8
Fuca1-2Galb1-3GlcNAcb1-3Galb1-4Glcb-Sp10
GalNAcb1-3Gala1-4Galb1-4GlcNAcb-Sp0
Galb1-4GlcNAcb1-6GalNAc-Sp14
GlcNAcb1-6(GlcNAcb1-3)GalNAca-Sp8
Neu5Aca2-3Galb1-3GalNAcb1-3Gala1-4Galb1-4Glcb-Sp0
Fuca1-2Galb1-4(6S)Glcb-Sp0
Neu5Aca2-6Galb1-4GlcNAcb1-2Mana1-6(GlcNAcb1-2Mana1-3)Manb1-4GlcNAcb1-4GlcNAcb-Sp12
Neu5Gcb2-6Galb1-4GlcNAc-Sp8
Galb1-3GlcNAcb1-2Mana1-6(Galb1-3GlcNAcb1-2Mana1-3)Manb1-4GlcNAcb1-4(Fuca1-6)GlcNAcb-Sp22
Fuca1-2Galb1-3GalNAca1-3(Fuca1-2)Galb1-4Glcb-Sp0
Galb1-4GlcNAcb1-2Mana-Sp0
Neu5Aca2-6Galb1-4GlcNAcb1-2Man-Sp0
GalNAcb1-4GlcNAcb1-2Mana-Sp0
Gala1-3(Fuca1-2)Galb1-3GalNAcb1-3Gala1-4Galb1-4Glc-Sp21
Fuca1-2Galb1-4GlcNAcb-Sp8
Gala1-3(Fuca1-2)Galb-Sp18
Gala1-3Galb1-4(Fuca1-3)GlcNAcb-Sp8
Gala1-4GlcNAcb-Sp8
Galb1-4(Fuca1-3)GlcNAcb1-3Galb1-4(Fuca1-3)GlcNAcb-Sp0
Galb1-4GlcNAcb1-3Galb1-4GlcNAcb-Sp0
GlcNAcb1-2Galb1-3GalNAca-Sp8
KDNa2-3Galb1-4GlcNAcb-Sp0
Mana1-2Mana1-6(Mana1-3)Mana1-6(Mana1-2Mana1-2Mana1-3)Manb1-4GlcNAcb1-4GlcNAcb-Sp12
Fuca1-2(6S)Galb1-4(6S)Glcb-Sp0
Neu5Aca2-3Galb1-4GlcNAcb1-3Galb1-4GlcNAcb-Sp0
GlcAb1-3GlcNAcb-Sp8
KDNa2-3Galb1-4Glc-Sp0
Fuca1-2Galb1-4GlcNAcb1-2Mana1-6(Fuca1-2Galb1-4GlcNAcb1-2Mana1-3)Manb1-4GlcNAcb1-4GlcNAcb-Sp20
Neu5Aca2-6GlcNAcb1-4GlcNAcb1-4GlcNAc-Sp21
Fuca1-2Galb1-4GlcNAcb1-3GalNAca-Sp14
Galb1-4(Fuca1-3)GlcNAcb1-3GalNAca-Sp14
Gala1-3Galb1-3GlcNAcb1-3GalNAc-Sp14
Fuca1-2Galb1-4GlcNAcb1-6(Fuca1-2Galb1-4GlcNAcb1-3)GalNAc-Sp14

Neu5Aca2-6Galb1-4GlcNAcb1-6GalNAca-Sp14
Galb1-4GlcNAcb1-2 Mana1-6(Galb1-4GlcNAcb1-4)(Galb1-4GlcNAcb1-2Mana1-3)Manb1-4GlcNAcb1-4(Fuca1-6)GlcNAc-Sp
Fuca1-4(Galb1-3)GlcNAcb1-2 Mana-Sp0
Galb1-3GlcNAcb1-6(Galb1-3)GalNAc-Sp14
Rhaa-Sp8
Fuca1-2Galb1-4(Fuca1-3)GlcNAcb1-3Galb1-4(Fuca1-3)GlcNAcb-Sp0
Galb1-3(Fuca1-4)GlcNAc-Sp0
Neu5Aca2-3Galb1-3(Fuca1-4)GlcNAcb1-3Galb1-4(Fuca1-3)GlcNAcb-Sp0
Neu5Aca2-3Galb1-3(6S)GalNAca-Sp8
Neu5Aca2-6GalNAcb1-4GlcNAcb-Sp0
Neu5Aca2-6Galb1-4GlcNAcb-Sp8
Neu5,9Ac2a2-3Galb1-3GlcNAcb-Sp0
Neu5Aca2-6Galb1-4GlcNAcb1-2Mana1-6Manb1-4GlcNAcb1-4GlcNAc-Sp12
KDNa2-6Galb1-4GlcNAc-Sp0
Fuca1-4(Fuca1-2Galb1-3)GlcNAcb1-2Mana1-3(Fuca1-4(Fuca1-2Galb1-3)GlcNAcb1-2Mana1-3)Manb1-4GlcNAcb1-4GlcNAc
Galb1-4GlcNAcb1-6(Galb1-4GlcNAcb1-2)Mana1-6(Galb1-4GlcNAcb1-4(Galb1-4GlcNAcb1-2)Mana1-3)Manb1-4GlcNAcb1-
GlcNAcb1-2Mana1-6(GlcNAcb1-4)(GlcNAcb1-4(GlcNAcb1-2)Mana1-3)Manb1-4GlcNAcb1-4GlcNAc-Sp21
Neu5Aca2-3Galb1-4GlcNAcb1-3Galb-Sp8
Gala1-3(Fuca1-2)Galb1-3GalNAcb-Sp8
Gala1-3Galb1-3GlcNAcb1-6GalNAca-Sp14
GalNAca1-3(Fuca1-2)Galb1-4 GlcNAcb1-2Mana-Sp0
Galb1-4GlcNAcb1-3Galb1-4GlcNAcb1-3Galb1-4GlcNAcb1-3Galb1-4GlcNAcb1-6(Galb1-4GlcNAcb1-3Galb1-4GlcNAcb1-3G
(6P)Mana-Sp8
Fuca1-2Galb1-3GlcNAcb1-3Galb1-4Glcb-Sp8
GalNAca1-3Galb-Sp8
Gala1-2Galb-Sp8
Galb1-4(Fuca1-3)GlcNAcb-Sp8
GlcNAcb1-4GlcNAcb1-4GlcNAcb1-4GlcNAcb1-4GlcNAcb1-Sp8
KDNa2-3Galb1-3GlcNAcb-Sp0
(3S)Galb1-4(Fuca1-3)(6S)GlcNAcb-Sp8
Neu5Aca2-6GalNAca-Sp8
Neu5Aca2-3Galb1-4GlcNAcb1-3Galb1-3GlcNAcb-Sp0
Neu5Aca2-3Galb1-4GlcNAcb1-2Mana1-6(Neu5Aca2-6Galb1-4GlcNAcb1-2Mana1-3)Manb1-4GlcNAcb1-4GlcNAcb-Sp12
Fuca1-2Galb1-4(Fuca1-3)GlcNAcb1-2Mana1-6(Fuca1-2Galb1-4(Fuca1-3)GlcNAcb1-2Mana1-3)Manb1-4GlcNAcb1-4GlcNAb
Galb1-4(Fuca1-3)GlcNAcb1-6(Fuca1-2Galb1-4GlcNAcb1-3)Galb1-4Glc-Sp21
GlcNAcb1-2Mana1-6(GlcNAcb1-4(GlcNAcb1-2)Mana1-3)Manb1-4GlcNAcb1-4GlcNAc-Sp21
Galb1-3GlcNAcb1-6(Galb1-3GlcNAcb1-2)Mana1-6(Galb1-3GlcNAcb1-2Mana1-3)Manb1-4GlcNAcb1-4GlcNAcb-Sp19
GlcNAcb1-2Mana1-6(GlcNAcb1-4)(GlcNAcb1-2Mana1-3)Manb1-4GlcNAcb1-4GlcNAc-Sp21
GalNAcb1-4Galb1-4Glcb-Sp0
Neu5Aca2-6Galb1-4GlcNAcb1-6(Neu5Aca2-6Galb1-4GlcNAcb1-2)Mana1-6(GlcNAcb1-4)(Neu5Aca2-6Galb1-4GlcNAcb1-2M
Neu5Aca2-3Galb1-3GlcNAcb1-6GalNAca-Sp14
Galb1-3GlcNAcb1-2Mana-Sp0
GlcNAcb1-3Galb1-4GlcNAcb1-3Galb1-4GlcNAcb1-6(GlcNAcb1-3Galb1-4GlcNAcb1-3Galb1-4GlcNAb1-2)Mana1-6(GlcNAcb
(3S)Galb1-4(Fuca1-3)GlcNAc-Sp0
(3S)Galb1-4(6S)GlcNAcb-Sp0
Fuca1-2Galb1-3(Fuca1-4)GlcNAcb-Sp8
Gala1-4Galb1-4GlcNAcb-Sp0
Galb1-3GlcNAcb1-3Galb1-4(Fuca1-3)GlcNAcb-Sp0

Galb1-3GalNAcb-Sp8
Galb1-3GalNAcb1-3Gala1-4Galb1-4Glcb-Sp0
Galb1-4GlcNAcb1-3Galb1-4(Fuca1-3)GlcNAcb1-3Galb1-4(Fuca1-3)GlcNAcb-Sp0
Galb1-4GlcNAcb1-3Galb1-4Glcb-Sp8
Galb1-4GlcNAcb1-6(Galb1-3)GalNAca-Sp8
Glca1-4Glca-Sp8
Neu5Aca2-3Galb1-4(Fuca1-3)GlcNAcb1-3Galb1-4(Fuca1-3)GlcNAcb1-3Galb1-4(Fuca1-3)GlcNAcb-Sp0
Galb1-4(Fuca1-3)(6S)GlcNAcb-Sp0
GalNAca1-3(Fuca1-2)Galb1-4GlcNAcb1-3Galb1-4GlcNAcb1-3Galb1-4GlcNAcb-Sp0
Neu5Aca2-3Galb1-4GlcNAcb1-6(Neu5Aca2-3Galb1-4GlcNAcb1-2)Mana1-6(GlcNAcb1-4)(Neu5Aca2-3Galb1-4GlcNAcb1-2N
Neu5Aca2-6Galb1-4GlcNAcb1-6(Neu5Aca2-6Galb1-4GlcNAcb1-2)Mana1-6(GlcNAcb1-4)(Neu5Aca2-6Galb1-4GlcNAcb1-4([illegible]
Galb1-4(Fuca1-3)GlcNAcb1-2Mana-Sp0
(6P)Galb1-4GlcNAcb-SP0
Gala1-3(Fuca1-2)Galb1-4GlcNAcb1-2Mana-Sp0
Gala1-3Galb1-3GlcNAcb1-2Mana-Sp0
Neu5Aca2-3Galb1-3GalNAcb1-4Galb1-4Glcb-Sp0
Galb1-4GlcNAcb1-3Galb1-4GlcNAcb1-3Galb1-4GlcNAcb1-3Galb1-4GlcNAcb1-2Mana1-6(Galb1-4GlcNAcb1-3Galb1-4GlcN
Gala1-3(Fuca1-2)Galb-Sp8
Galb1-3(Fuca1-4)GlcNAcb1-3Galb1-4(Fuca1-3)GlcNAcb-Sp0
GlcNAcb1-4GlcNAcb1-4GlcNAcb-Sp8
Neu5Aca2-3Galb1-3GalNAca-Sp14
GalNAcb1-4(Neu5Aca2-8Neu5Aca2-3)Galb1-4Glcb-Sp0
Neu5Aca2-8Neu5Aca2-8Neu5Aca-Sp8
Neu5Aca2-6Galb1-4GlcNAcb1-3Galb1-4(Fuca1-3)GlcNAcb1-3Galb1-4(Fuca1-3)GlcNAcb-Sp0
(6S)Galb1-4(6S)GlcNAcb-Sp0
Mana1-2Mana1-6(Mana1-3)Mana1-6(Mana1-2Mana1-2Mana1-3)Mana-Sp9
Galb1-3GlcNAcb1-2Mana1-6(Galb1-3GlcNAcb1-2Mana1-3)Manb1-4GlcNAcb1-4GlcNAcb-Sp19
GlcNAca1-4Galb1-4GlcNAcb1-3Galb1-4GlcNAcb1-3Galb1-4GlcNAcb-Sp0
GlcNAca1-4Galb1-3GlcNAcb-Sp0
Galb1-4GlcNAcb1-2Mana1-6(Galb1-4GlcNAcb1-4(Galb1-4GlcNAcb1-2)Mana1-3)Manb1-4GlcNAcb1-4GlcNAc-Sp21
Neu5Aca2-6GalNAcb1-4(6S)GlcNAcb-Sp8
Galb1-3GalNAcb1-4(Neu5Aca2-8Neu5Aca2-8Neu5Aca2-3)Galb1-4Glcb-Sp21
GlcNAcb1-6(GlcNAcb1-4)(GlcNAcb1-3)GlcNAc-Sp8
Fuca1-2Galb1-3GalNAcb1-4(Neu5Aca2-3)Galb1-4Glcb-Sp9
Fuca1-2Galb1-4(Fuca1-3)GlcNAcb-Sp0
Fucb1-3GlcNAcb-Sp8
Galb1-3Galb-Sp8
GlcNAcb1-6(GlcNAcb1-3)Galb1-4GlcNAcb-Sp8
GlcNAcb1-3Galb1-4GlcNAcb1-3Galb1-4GlcNAcb-Sp0
GlcAb1-3Galb-Sp8
Mana1-2Mana1-6(Mana1-2Mana1-3)Mana1-6(Mana1-2Mana1-2Mana1-3)Manb1-4GlcNAcb1-4GlcNAcb-Sp12
Neu5Aca2-8Neu5Aca2-8Neu5Aca2-3Galb1-4Glcb-Sp0
Neu5Aca2-6(Neu5Aca2-3Galb1-3)GalNAca-Sp14
Neu5Aca2-3Galb1-3GlcNAcb-Sp0
Neu5Aca2-3Galb1-4GlcNAcb-Sp0
Neu5Aca2-6Galb1-4GlcNAcb-Sp0
Mana1-6(Galb1-4GlcNAcb1-2Mana1-3)Manb1-4GlcNAcb1-4GlcNAcb-Sp12
Galb1-4GlcNAcb1-2Mana1-6(GlcNAcb1-2Mana1-3)Manb1-4GlcNAcb1-4GlcNAc-Sp12

Galb1-4(Fuca1-3)GlcNAcb1-2Mana1-6(Galb1-4(Fuca1-3)GlcNAcb1-2Mana1-3)Manb1-4GlcNAcb1-4(Fuca1-6)GlcNAcb-Sp2[illegible]
Neu5Aca2-6Galb1-4GlcNAcb1-2Mana1-6(GlcNAcb1-4)(Neu5Aca2-6Galb1-4GlcNAcb1-2Mana1-3)Manb1-4GlcNAcb1-4Glc[illegible]
Gala1-3(Fuca1-2)Galb1-4GlcNAcb1-6GalNAca-Sp14
GlcNAcb1-3Galb1-4GlcNAcb1-6(GlcNAcb1-3)Galb1-4GlcNAc-Sp0
GlcNAcb1-3Galb1-4GlcNAcb1-2Mana1-6(GlcNAcb1-3Galb1-4GlcNAcb1-2Mana1-3)Manb1-4GlcNAcb1-4(Fuca1-6)GlcNAc[illegible]
(3S)Galb1-4(6S)GlcNAcb-Sp8
(6S)Galb1-4Glcb-Sp8
(6S)Galb1-4GlcNAcb-Sp8
Neu5Aca2-6Galb1-4GlcNAcb1-2Mana1-6(Neu5Aca2-6Galb1-4GlcNAcb1-2Mana1-3)Manb1-4GlcNAcb1-4GlcNAcb-Sp12
Neu5Aca2-6Galb1-4GlcNAcb1-2Mana1-6(Neu5Aca2-6Galb1-4GlcNAcb1-2Man-a1-3)Manb1-4GlcNAcb1-4GlcNAcb-Sp21
Fuca1-2Galb1-4(Fuca1-3)GlcNAcb1-3Galb1-4(Fuca1-3)GlcNAcb1-3Galb1-4(Fuca1-3)GlcNAcb-Sp0
Gala1-3(Fuca1-2)Galb1-3GlcNAcb-Sp8
Gala1-3(Fuca1-2)Galb1-4(Fuca1-3)GlcNAcb-Sp0
Gala1-4(Fuca1-2)Galb1-4GlcNAcb-Sp8
Galb1-3(Fuca1-4)GlcNAc-Sp8
Galb1-3GlcNAcb-Sp0
GlcNAcb1-3Galb1-4GlcNAcb-Sp0
Mana1-2Mana1-2Mana1-3Mana-Sp9
Neu5Aca2-3Galb1-4(6S)GlcNAcb-Sp8
Neu5Aca2-3Galb1-4GlcNAcb1-3Galb1-4GlcNAcb1-3Galb1-4GlcNAcb-Sp0
4S(3S)Galb1-4GlcNAcb-Sp0
Gala1-4Galb1-4GlcNAcb1-3Galb1-4Glcb-Sp0
GalNAcb1-3Gala1-4Galb1-4GlcNAcb1-3Galb1-4Glcb-Sp0
KDNa2-3Galb1-4(Fuca1-3)GlcNAc-Sp0
Gala1-3Galb1-4GlcNAcb1-2Mana1-6(Gala1-3Galb1-4GlcNAcb1-2Mana1-3)Manb1-4GlcNAcb1-4GlcNAcb-Sp20
Galb1-4GlcNAcb1-6(Fuca1-4(Fuca1-2Galb1-3)GlcNAcb1-3)Galb1-4Glc-Sp21
Gala1-4Galb1-4GlcNAcb1-2Mana1-6(Gala1-4Galb1-4GlcNAcb1-2Mana1-3)Manb1-4GlcNAcb1-4GlcNAcb-Sp24
GlcNAcb1-6(GlcNAcb1-2)Mana1-6(GlcNAcb1-4)(GlcNAcb1-2Mana1-3)Manb1-4GlcNAcb1-4GlcNAc-Sp21
Galb1-4GlcNAcb1-2Mana1-6(GlcNAcb1-4)(Galb1-4GlcNAcb1-4(Galb1-4GlcNAcb1-2)Mana1-3)Manb1-4GlcNAcb1-4GlcNA[illegible]
Neu5Aca2-6Galb1-4GlcNAcb1-4Mana1-6(GlcNAcb1-4)(Neu5Aca2-6Galb1-4GlcNAcb1-4(Neu5Aca2-6Galb1-4GlcNAcb1-2)[illegible]
Neu5Aca2-6Galb1-4 GlcNAcb1-6(Neu5Aca2-6Galb1-4GlcNAcb1-3)GalNAca-Sp14
Gala1-3Galb1-4GlcNAcb1-6GalNAca-Sp14
GalNAcb1-4(Fuca1-3)(6S)GlcNAcb-Sp8
(3S)GalNAcb1-4(Fuca1-3)GlcNAcb-Sp8
GlcNAcb1-2 Mana1-6(GlcNAcb1-4)(GlcNAcb1-2Mana1-3)Manb1-4GlcNAcb1-4(Fuca1-6)GlcNAc-Sp21
GlcNAcb1-3Galb1-4GlcNAcb1-6(Galb1-3)GalNAca-Sp14
GlcNAcb1-3Galb1-4GlcNAcb1-3Galb1-4GlcNAcb1-3GalNAca-Sp14
Neu5Acb-Sp8
Galb1-4GlcNAcb1-6(Galb1-4GlcNAcb1-3)GalNAc-Sp14
(3S)Galb1-3GalNAca-Sp8
Fuca1-2Galb1-4GlcNAcb1-3Galb1-4GlcNAcb-Sp0
Fuca1-3GlcNAcb-Sp8
GalNAca1-3GalNAcb-Sp8
GalNAca1-4(Fuca1-2)Galb1-4GlcNAcb-Sp8
GalNAcb1-3(Fuca1-2)Galb-Sp8
GalNAcb1-4GlcNAcb-Sp8
Gala1-3(Fuca1-2)Galb1-4Glcb-Sp0
Galb1-2Galb-Sp8

Galb1-4GlcNAcb1-6GalNAca-Sp8
Galb1-4GlcNAcb1-3GalNAca-Sp8
Glca1-6Glca1-6Glcb-Sp8
Mana1-2Mana1-2Mana1-6(Mana1-3)Mana-Sp9
Neu5Aca2-3Galb1-3GalNAcb1-4(Neu5Aca2-3)Galb1-4Glcb-Sp0
Neu5Aca2-8Neu5Aca2-8Neu5Acb-Sp8
GalNAca1-3(Fuca1-2)Galb1-4GlcNAcb1-3Galb1-4GlcNAcb-Sp0
Gala1-3(Fuca1-2)Galb1-4GlcNAcb1-2Mana1-6(Gala1-3(Fuca1-2)Galb1-4GlcNAcb1-2Mana1-3)Manb1-4GlcNAcb1-4GlcNAc
GalNAcb1-3Gala1-6Galb1-4Glcb-Sp8
Galb1-4GlcNAcb1-6(Fuca1-2Galb1-3GlcNAcb1-3)Galb1-4Glc-Sp21
Neu5Aca2-3Galb1-4GlcNAcb1-6GalNAca-Sp14
GalNAca1-3(Fuca1-2)Galb1-3GlcNAcb1-6GalNAca-Sp14
(6S)(4S)GalNAcb1-4GlcNAc-Sp8
Neu5Aca-Sp11
GlcN(Gc)b-Sp8
(3S)Galb1-4GlcNAcb-Sp0
Fuca1-2Galb1-4(Fuca1-3)GlcNAcb-Sp8
Fuca1-4GlcNAcb-Sp8
GlcNAca1-6Galb1-4GlcNAcb-Sp8
GlcNAcb1-6GalNAca-Sp8
GlcNAcb1-6GalNAca-Sp14
GalNAcb1-4(Neu5Aca2-8Neu5Aca2-8Neu5Aca2-3)Galb1-4Glcb-Sp0
Neu5Gca2-6Galb1-4GlcNAcb-Sp0
GlcNAcb1-4GlcNAcb-Sp10
Gala1-3(Fuca1-2)Galb1-4(Fuca1-3)Glcb-Sp21
Fuca1-3GlcNAcb1-6(Galb1-4GlcNAcb1-3)Galb1-4Glc-Sp21
GalNAca1-3(Fuca1-2)Galb1-4GlcNAcb1-6(GalNAca1-3(Fuca1-2)Galb1-4GlcNAcb1-3)GalNAc-Sp14
Mana1-6(Mana1-3)Manb1-4GlcNAcb1-4(Fuca1-6)GlcNAcb-Sp19
(3S)Galb1-3(Fuca1-4)GlcNAcb-Sp0
(6S)GalNAcb1-4GlcNAc-Sp8
Neu5Aca2-3Galb1-4GlcNAcb1-3Galb1-4GlcNAcb1-6(Neu5Aca2-3Galb1-4GlcNAcb1-3Galb1-4GlcNAcb1-3)GalNAca-Sp14
GalNAcb-Sp8
(3S)Galb1-4(6S)Glcb-Sp8
GalNAca1-3(Fuca1-2)Galb-Sp18
Gala1-4(Gala1-3)Galb1-4GlcNAcb-Sp8
GlcNAcb1-6(Galb1-3)GalNAca-Sp8
Neu5Aca2-3Galb1-4GlcNAcb1-6(Galb1-3)GalNAca-Sp14
MurNAcb1-4GlcNAcb-Sp10
Mana1-2Mana1-6(Mana1-2Mana1-3)Mana1-6(Mana1-2Mana1-2Mana1-3)Mana-Sp9
Galb1-4GlcNAcb1-2Mana1-6(Neu5Aca2-6Galb1-4GlcNAcb1-2Mana1-3)Manb1-4GlcNAcb1-4GlcNAcb-Sp12
Neu5Aca2-6Galb1-4GlcNAcb1-2Mana1-6(Mana1-3)Manb1-4GlcNAcb1-4GlcNAc-Sp12
Galb1-3GalNAca1-3(Fuca1-2)Galb1-4Glc-Sp0
GalNAca1-3(Fuca1-2)Galb1-4GlcNAcb1-3GalNAca-Sp14
Gala1-3(Fuca1-2)Galb1-3GlcNAcb1-3GalNAc-Sp14
Galb1-4GlcNAcb1-6(Galb1-4GlcNAcb1-2)Mana1-6(GlcNAcb1-4)(Galb1-4GlcNAcb1-4(Galb1-4GlcNAcb1-2)Mana1-3)Manb1
Neu5Aca2-3Galb1-3GlcNAcb1-2Mana1-6(GlcNAcb1-4)(Neu5Aca2-3Galb1-3GlcNAcb1-2Mana1-3)Manb1-4GlcNAcb1-4GlcI
Neu5Aca2-3Galb1-3GlcNAcb1-2Mana-Sp0
Neu5Aca2-3Galb1-4GlcNAcb1-3Galb1-4GlcNAcb1-6(Galb1-3)GalNAca-Sp14

GlcNAcb1-6(Neu5Aca2-3Galb1-3)GalNAca-Sp14
Galb1-4GlcNAcb1-6(Galb1-4GlcNAcb1-3)GalNAca-Sp8
(3S)Galb1-3(Fuca1-4)GlcNAcb-Sp8
(3S)Galb1-3GlcNAcb-Sp0
Gala1-6Glcb-Sp8
Galb1-4(Fuca1-3)GlcNAcb1-3Galb1-4(Fuca1-3)GlcNAcb1-3Galb1-4(Fuca1-3)GlcNAcb-Sp0
GlcNAcb1-6Galb1-4GlcNAcb-Sp8
GlcAa-Sp8
Mana1-6(Mana1-3)Mana1-6(Mana1-2Mana1-3)Manb1-4GlcNAcb1-4GlcNAcb-Sp12
GalNAcb1-4(Neu5Aca2-3)Galb1-4Glcb-Sp0
Neu5Aca2-6(Neu5Aca2-3Galb1-3)GalNAca-Sp8
Neu5Aca2-6Galb-Sp8
Neu5Aca2-6Galb1-4GlcNAcb1-2Mana1-6(Neu5Aca2-3Galb1-4GlcNAcb1-2Mana1-3)Manb1-4GlcNAcb1-4GlcNAcb-Sp12
Galb1-4(Fuca1-3)GlcNAcb1-2Mana1-6(Galb1-4(Fuca1-3)GlcNAcb1-2Mana1-3)Manb1-4GlcNAcb1-4GlcNAcb-Sp20
Neu5,9Ac2a2-3Galb1-4GlcNAcb-Sp0
GalNAca1-3(Fuca1-2)Galb1-3GlcNAcb1-2Mana1-6(GalNAca1-3(Fuca1-2)Galb1-3GlcNAcb1-2Mana1-3)Manb1-4GlcNAcb1-4
Neu5Aca2-6Galb1-4GlcNAcb1-3GalNAc-Sp14
Galb1-3GlcNAcb1-3Galb1-4GlcNAcb1-6(Galb1-3GlcNAcb1-3)Galb1-4Glcb-Sp0
Gala1-3Galb1-4GlcNAcb1-3GalNAca-Sp14
Neu5Aca2-3Galb1-3GalNAcb1-4(Neu5Aca2-8Neu5Aca2-3)Galb1-4Glcb-Sp0
Galb1-4GlcNAcb1-6(Galb1-4GlcNAcb1-2)Mana1-6(GlcNAcb1-4)(Galb1-4GlcNAcb1-2Mana1-3)Manb1-4GlcNAcb1-4GlcNAc
Galb1-4Galb-Sp10
GalNAcb1-6GalNAcb-Sp8
Neu5Aca2-3Galb1-4GlcNAcb1-6(Neu5Aca2-3Galb1-4GlcNAcb1-2)Mana1-6(GlcNAcb1-4)(Neu5Aca2-3Galb1-4GlcNAcb1-4(
Galb1-4GlcNAcb1-3Galb1-4GlcNAcb1-2Mana1-6(Galb1-4GlcNAcb1-3Galb1-4GlcNAcb1-2Mana1-3)Manb1-4GlcNAcb1-4(F
Galb1-4GlcNAcb1-3Galb1-4GlcNAcb1-3Galb1-4GlcNAcb1-3Galb1-4GlcNAcb1-3Galb1-4GlcNAcb1-2Mana1-6(Galb1-4GlcN
GlcNAcb1-3Galb1-4GlcNAcb1-6(GlcNAcb1-3Galb1-4GlcNAcb1-3)GalNAca-Sp14
Gala-Sp8
Neu5Aca-Sp8
Galb1-3GlcNAcb1-3Galb1-4GlcNAcb-Sp0
Galb1-3GlcNAcb1-3Galb1-4Glcb-Sp10
Galb1-4(6S)Glcb-Sp0
Galb1-4(6S)Glcb-Sp8
Galb1-4Glcb-Sp8
Neu5Aca2-6(Neu5Aca2-3)GalNAca-Sp8
Neu5Aca2-3Galb1-4(Neu5Aca2-3Galb1-3)GlcNAcb-Sp8
Neu5Gca2-3Galb1-3(Fuca1-4)GlcNAcb-Sp0
GlcNAcb1-3Man-Sp10
Mana1-6(Mana1-3)Mana1-6(Mana1-3)Manb-Sp10
(6S)Galb1-3(6S)GlcNAc-Sp0
Neu5Aca2-6Galb1-4GlcNAcb1-6(Galb1-3GlcNAcb1-3)Galb1-4Glcb-Sp21
(3S)GalNAcb1-4GlcNAc-Sp8
Gala1-3(Fuca1-2)Galb1-3GlcNAcb1-6GalNAc-Sp14
GalNAcb1-3GlcNAcb-Sp0
GalNAcb1-4GlcNAcb1-3GalNAcb1-4GlcNAcb-Sp0
(6S)Galb1-4(6S)Glcb-Sp8
Mana1-6(Mana1-3)Manb1-4GlcNAcb1-4GlcNAcb-Sp12
GlcNAcb1-2Mana1-6(GlcNAcb1-2Mana1-3)Manb1-4GlcNAcb1-4GlcNAcb-Sp12

Neu5Aca2-6Galb1-4GlcNAcb1-2Mana1-6(Neu5Aca2-6Galb1-4GlcNAcb1-2Mana1-3)Manb1-4GlcNAcb1-4GlcNAcb-Sp24
Fuca1-2Galb1-3GalNAcb1-3Gala1-4Galb1-4Glcb-Sp9
GalNAca1-3(Fuca1-2)Galb-Sp8
Gala1-3(Fuca1-2)Galb1-3GlcNAcb-Sp0
Galb1-3GlcNAcb-Sp8
Galb1-4GlcNAcb-Sp23
GlcNAcb1-3Galb1-4Glcb-Sp0
GlcNAcb1-4-MDPLys
Neu5Aca2-3Galb1-4(Fuca1-3)GlcNAcb-Sp0
Neu5Aca2-6Galb1-4GlcNAcb1-3Galb1-4GlcNAcb1-3Galb1-4GlcNAcb-Sp0
Galb1-3GlcNAcb1-3GalNAca-Sp14
GlcNAcb1-2Mana1-6(Galb1-4GlcNAcb1-2Mana1-3)Manb1-4GlcNAcb1-4GlcNAc-Sp12
Galb1-3GlcNAcb1-6Galb1-4GlcNAcb-Sp0
Fuca1-2Galb1-3GlcNAcb1-3GalNAc-Sp14
GlcNAcb1-6(GlcNAcb1-2)Mana1-6(GlcNAcb1-4)(GlcNAcb1-4(GlcNAcb1-2)Mana1-3)Manb1-4GlcNAcb1-4GlcNAc-Sp21
Galb1-3GlcNAcb1-6GalNAca-Sp14
Galb1-3GalNAcb1-3Gal-Sp21
Neu5Gca2-8Neu5Gca2-3Galb1-4GlcNAc-Sp0
Neu5Gca2-8Neu5Gca2-6Galb1-4GlcNAc-Sp0
(3S)GlcAb1-3Galb1-4GlcNAcb1-2Mana-Sp0
GlcNAcb1-3Galb1-4GlcNAcb1-3GalNAca-Sp14
GlcNAcb1-3Fuca-Sp21
Glcb-Sp8
(3S)Galb1-4(6S)Glcb-Sp0
(6S)Galb1-4Glcb-Sp0
Neu5Aca2-3(6S)Galb1-4GlcNAcb-Sp8
GlcNAcb1-2Mana1-6(GlcNAcb1-2Mana1-3)Manb1-4GlcNAcb1-4GlcNAcb-Sp13
Galb1-4GlcNAcb1-2Mana1-6(Galb1-4GlcNAcb1-2Mana1-3)Manb1-4GlcNAcb1-4GlcNAcb-Sp12
Fuca1-2Galb1-3GlcNAcb-Sp8
Galb1-3GalNAca-Sp14
Galb1-4GalNAca1-3(Fuca1-2)Galb1-4GlcNAcb-Sp8
Galb1-4GlcNAcb1-3Galb1-4GlcNAcb1-3Galb1-4GlcNAcb-Sp0
Neu5Aca2-3Galb1-4(Fuca1-3)(6S)GlcNAcb-Sp8
Neu5Aca2-6Galb1-4(6S)GlcNAcb-Sp8
Galb1-4GlcNAcb1-3Galb1-3GlcNAcb-Sp0
Galb1-4GlcNAcb1-6(Galb1-4GlcNAcb1-3)Galb1-4GlcNAc-Sp0
GlcNAca1-4Galb1-4GlcNAcb1-3Galb1-4Glcb-Sp0
GlcNAca1-4Galb1-4GlcNAcb1-3Galb1-4GlcNAcb-Sp0
Gala1-3Galb1-4(Fuca1-3)GlcNAcb1-2Mana1-6(Gala1-3Galb1-4(Fuca1-3)GlcNAcb1-2Mana1-3)Manb1-4GlcNAcb1-4GlcNAc
GalNAca1-3GalNAcb1-3Gala1-4Galb1-4Glcb-Sp0
(4S)GalNAcb-Sp10
Galb1-4GlcNAcb1-2 Mana1-6(GlcNAcb1-4)(Galb1-4GlcNAcb1-2Mana1-3)Manb1-4GlcNAcb1-4(Fuca1-6)GlcNAc-Sp21
Galb-Sp8
(3S)Galb1-3GlcNAcb-Sp8
(3S)Galb1-4GlcNAcb-Sp8
Fuca1-2Galb1-3GalNAca-Sp14
GalNAca1-3(Fuca1-2)Galb1-3GlcNAcb-Sp0
GalNAca1-3(Fuca1-2)Galb1-4Glcb-Sp0

Gala1-3Galb1-4Glcb-Sp0
Galb1-4Glcb-Sp0
GlcNAcb1-3GalNAca-Sp14
GlcNAcb1-4Galb1-4GlcNAcb-Sp8
GalNAcb1-4(Neu5Aca2-8Neu5Aca2-8Neu5Aca2-8Neu5Aca2-3)Galb1-4Glcb-Sp0
Neu5Aca2-6Galb1-4Glcb-Sp8
Neu5Aca2-6GlcNAcb1-4GlcNAc-Sp21
Galb1-3GlcNAca1-3Galb1-4GlcNAcb-Sp8
GalNAca1-3(Fuca1-2)Galb1-4GlcNAcb1-6GalNAc-Sp14
Neu5Aca2-8Neu5Gca2-3Galb1-4GlcNAc-Sp0
(3S)GlcAb1-3Galb1-4GlcNAcb1-3Galb1-4Glc-Sp0
GlcNAcb1-3Galb1-4GlcNAcb1-3Galb1-4GlcNAcb1-3Galb1-4GlcNAcb1-2Mana1-6(GlcNAcb1-3Galb1-4GlcNAcb1-3Galb1-4
Mana-Sp8
GlcNAcb-Sp0
(3S)Galb1-4(Fuca1-3)GlcNAc-Sp8
Fuca1-2Galb1-3GalNAcb1-4(Neu5Aca2-3)Galb1-4Glcb-Sp0
Fuca1-2Galb1-4GlcNAcb1-3Galb1-4GlcNAcb1-3Galb1-4GlcNAcb-Sp0
GalNAca1-3(Fuca1-2)Galb1-4GlcNAcb-Sp8
Gala1-3GalNAca-Sp8
GlcNAcb1-3Galb-Sp8
Mana1-2Mana1-6(Mana1-2Mana1-3)Mana-Sp9
Neu5Aca2-3Galb1-3GalNAca-Sp8
Neu5Aca2-6Galb1-4GlcNAcb1-3Galb1-4GlcNAcb-Sp0
Galb1-3(Fuca1-4)GlcNAcb1-3Galb1-3(Fuca1-4)GlcNAcb-Sp0
(6S)GlcNAcb1-3Galb1-4GlcNAcb-Sp0
Galb1-4GlcNAcb1-2Mana1-6(Mana1-3)Manb1-4GlcNAcb1-4GlcNAcb-Sp12
Gala1-3(Fuca1-2)Galb1-4GlcNAcb1-3GalNAca-Sp14
(3S)GalNAcb1-4(3S)GlcNAc-Sp8
Galb1-4GlcNAcb1-3Galb1-4GlcNAcb1-6(Galb1-4GlcNAcb1-3Galb1-4GlcNAb1-2)Mana1-6(Galb1-4GlcNAcb1-3Galb1-4Glc
Neu5,9Ac2a2-6Galb1-4GlcNAcb-Sp8
Mana1-6(Mana1-3)Manb1-4GlcNAcb1-4GlcNAcb-Sp13
Fuca1-2Galb1-3GlcNAcb-Sp0
Neu5Aca2-6(Galb1-3)GalNAca-Sp14
Galb1-4GlcNAcb-Sp0
Mana1-6(Mana1-3)Mana-Sp9
Neu5Aca2-3GalNAcb1-4GlcNAcb-Sp0
Neu5Aca2-3Galb1-3(Fuca1-4)GlcNAcb-Sp8
Neu5Aca2-3Galb-Sp8
Neu5Acb2-6GalNAca-Sp8
Neu5Acb2-6Galb1-4GlcNAcb-Sp8
Neu5Aca2-3Galb1-4GlcNAcb1-6(Neu5Aca2-3Galb1-3)GalNAca-Sp14
Gala1-3(Fuca1-2)Galb1-4GlcNAcb1-6(Gala1-3(Fuca1-2)Galb1-4GlcNAcb1-3)GalNAc-Sp14
Neu5Aca2-6Galb1-4GlcNAcb1-6(Fuca1-2Galb1-4(Fuca1-3)GlcNAcb1-3)Galb1-4Glc-Sp21
Fuca1-2(6S)Galb1-3GlcNAcb-Sp0
GalNAcb1-4(6S)GlcNAc-Sp8
GlcNAcb1-3Galb1-4GlcNAcb1-3Galb1-4GlcNAcb1-3Galb1-4GlcNAcb1-3Galb1-4GlcNAcb1-6(GlcNAcb1-3Galb1-4GlcNAcb
Neu5Aca2-3Galb1-4GlcNAcb1-3Galb1-4GlcNAcb1-2Mana1-6(Neu5Aca2-3Galb1-4GlcNAcb1-3Galb1-4GlcNAcb1-2Mana1-
Neu5Aca2-6Galb1-4GlcNAcb1-3Galb1-4GlcNAcb1-6(Neu5Aca2-6Galb1-4GlcNAcb1-3Galb1-4GlcNAcb1-3)GalNAca-Sp14

Fuca1-2Galb1-3GalNAca-Sp8
Gala1-3(Fuca1-2)Galb1-4GlcNAc-Sp0
GlcNAcb1-6(GlcNAcb1-4)GalNAca-Sp8
Manb1-4GlcNAcb-Sp0
GlcNAcb1-6(Galb1-4GlcNAcb1-3)Galb1-4GlcNAc-Sp0
Mana1-6Manb-Sp10
Galb1-4GlcNAcb1-2Mana1-3Manb1-4GlcNAcb1-4GlcNAc-Sp12
Neu5Aca2-6Galb1-4GlcNAcb1-6(Fuca1-2Galb1-3GlcNAcb1-3)Galb1-4Glc-Sp21
Fuca1-2Galb1-4GlcNAcb1-2Mana-Sp0
GlcNAcb1-3Galb1-4GlcNAcb1-2Mana1-6(GlcNAcb1-3Galb1-4GlcNAcb1-2Mana1-3)Manb1-4GlcNAcb1-4GlcNAcb-Sp12
Neu5Aca2-8Neu5Aca2-3Galb1-3GalNAcb1-4(Neu5Aca2-3)Galb1-4Glc-Sp21
(3S)Galb1-4Glcb-Sp8
Gala1-4Galb1-4Glcb-Sp0
GlcNAca1-3Galb1-4GlcNAcb-Sp8
Mana1-2Mana1-3Mana-Sp9
GalNAcb1-4(Neu5Aca2-3)Galb1-4GlcNAcb-Sp8
Neu5Aca2-3Galb1-3GlcNAcb1-3Galb1-4GlcNAcb-Sp0
Galb1-4(Fuca1-3)(6S)Glcb-Sp0
Neu5Aca2-6Galb1-4GlcNAcb1-2Mana1-6(Galb1-4GlcNAcb1-2Mana1-3)Manb1-4GlcNAcb1-4GlcNAcb-Sp12
GlcNAcb1-4GlcNAcb-Sp12
Galb1-4GlcNAcb1-2Mana1-6(GlcNAcb1-4)(Galb1-4GlcNAcb1-2Mana1-3)Manb1-4GlcNAcb1-4GlcNAc-Sp21
Neu5Gca2-8Neu5Aca2-3Galb1-4GlcNAc-Sp0
GlcNAcb1-3Galb1-4GlcNAcb1-3Galb1-4GlcNAcb1-3Galb1-4GlcNAcb1-6(GlcNAcb1-3Galb1-4GlcNAcb1-3Galb1-4GlcNAcb[illegible]
Neu5Aca2-3Galb1-4GlcNAcb1-3Galb1-4GlcNAcb1-3GalNAca-Sp14
GalNAcb1-4(Fuca1-3)GlcNAcb-Sp0
GalNAcb1-4GlcNAcb-Sp0
GlcNAcb1-3Galb1-4GlcNAcb-Sp8
Neu5Aca2-3Galb1-3(6S)GlcNAc-Sp8
Galb1-4(Fuca1-3)GlcNAcb1-6(Galb1-3GlcNAcb1-3)Galb1-4Glc-Sp21
Galb1-3GlcNAcb1-3Galb1-4(Fuca1-3)GlcNAcb1-6(Galb1-3GlcNAcb1-3)Galb1-4Glc-Sp21
Galb1-4GlcNAcb1-3Galb1-4GlcNAcb1-3Galb1-4GlcNAcb1-3Galb1-4GlcNAcb1-3Galb1-4GlcNAcb1-3Galb1-4GlcNAcb1-2M
Fuca-Sp8
(6S)(4S)Galb1-4GlcNAcb-Sp0
Fuca1-2Galb-Sp8
Neu5Acb2-6(Galb1-3)GalNAca-Sp8
GlcNAcb1-3Galb1-4GlcNAcb1-6(GlcNAcb1-3Galb1-3)GalNAca-Sp14
Neu5Aca2-6Galb1-4GlcNAcb1-3Galb1-4GlcNAcb1-6(Galb1-3)GalNAca-Sp14
Galb1-3GalNAca-Sp8
Neu5Aca2-3Galb1-4(Fuca1-3)GlcNAcb-Sp8
Neu5Aca2-3Galb1-4GlcNAcb-Sp8
Neu5Aca2-6Galb1-4Glcb-Sp0
Neu5Aca2-6Galb1-4GlcNAcb1-3Galb1-3GlcNAcb-Sp0
Mana1-6(Neu5Aca2-6Galb1-4GlcNAcb1-2Mana1-3)Manb1-4GlcNAcb1-4GlcNAc-Sp12
Galb1-4(Fuca1-3)GlcNAcb1-6(Fuca1-4(Fuca1-2Galb1-3)GlcNAcb1-3)Galb1-4Glc-Sp21
GalNAca1-3GalNAcb1-3Gala1-4Galb1-4GlcNAcb-Sp0
Galb1-3GalNAcb1-4Galb1-4Glcb-Sp8
Galb1-4GlcNAcb1-3GalNAc-Sp14
Galb1-4GlcNAcb-Sp8

KDNa2-3Galb1-3GalNAca-Sp14
GalNAca1-3(Fuca1-2)Galb1-4GlcNAcb1-2Mana1-6(GalNAca1-3(Fuca1-2)Galb1-4GlcNAcb1-2Mana1-3)Manb1-4GlcNAcb1-[illegible]
Galb1-4GlcNAcb1-3Galb1-4GlcNAcb1-3Galb1-4GlcNAcb1-2Mana1-6(Galb1-4GlcNAcb1-3Galb1-4GlcNAcb1-3Galb1-4GlcN[illegible]
GlcNAcb1-3Galb1-4GlcNAcb1-3Galb1-4GlcNAcb1-3Galb1-4GlcNAcb1-3Galb1-4GlcNAcb1-2Mana1-6(GlcNAcb1-3Galb1-4[illegible]
Galb1-4GalNAcb1-3(Fuca1-2)Galb1-4GlcNAcb-Sp8
Mana1-6(Mana1-2Mana1-3)Mana1-6(Mana1-2Mana1-3)Manb1-4GlcNAcb1-4GlcNAcb-Sp12
Neu5Aca2-3Galb1-4(Fuca1-3)GlcNAcb1-3Galb-Sp8
Neu5Aca2-3Galb1-3GlcNAcb1-2Mana1-6(Neu5Aca2-3Galb1-3GlcNAcb1-2Mana1-3)Manb1-4GlcNAcb1-4GlcNAc-Sp19
GlcNAcb1-6(GlcNAcb1-2)Mana1-6(GlcNAcb1-4)(GlcNAcb1-4(GlcNAcb1-2)Mana1-3)Manb1-4GlcNAcb1-4(Fuca1-6)GlcNAc-[illegible]
GalNAca-Sp8
(3S)Galb-Sp8
Gala1-3GalNAca-Sp16
Neu5Aca2-3GalNAca-Sp8
GlcNAca1-4Galb1-4GlcNAcb-Sp0
Neu5Aca2-3Galb1-4GlcNAcb1-2Mana1-6(GlcNAcb1-4)(Neu5Aca2-3Galb1-4GlcNAcb1-2Mana1-3)Manb1-4GlcNAcb1-4Glc[illegible]
Galb1-3(6S)GlcNAcb-Sp8
Gala1-4Galb1-4GlcNAcb-Sp8
Galb1-4GlcNAcb1-6(Galb1-3)GalNAc-Sp14
Galb1-4GlcNAcb1-6(Galb1-4GlcNAcb1-2)Mana1-6(GlcNAcb1-4)Galb1-4GlcNAcb1-4(Gal b1-4GlcNAcb1-2)Mana1-3)Manb1[illegible]
Gala1-3Galb1-3GlcNAcb-Sp0
Galb1-3GalNAcb1-4(Neu5Aca2-3)Galb1-4Glcb-Sp0
GalNAcb1-4(Neu5Aca2-3)Galb1-4GlcNAcb-Sp0
Neu5Aca2-8Neu5Aca2-3Galb1-4Glcb-Sp0
Manb-Sp8
Neu5Aca2-3Galb1-4Glcb-Sp0

Average	STDEV	%CV
94	4	4
81	15	19
64	9	13
56	3	5
48	21	44
47	2	5
44	2	6
41	15	38
38	2	6
35	2	6
34	19	56
33	3	8
33	3	10
33	5	15
32	5	15
31	4	13
31	6	20
30	2	5
30	5	16
30	5	16
29	22	75
29	4	14
29	11	39
29	5	18
29	8	29
29	2	6
29	2	8
28	10	37
27	4	15
27	8	29
27	3	12
27	2	6
27	2	9
27	1	5
27	3	12
27	1	3
27	7	25
27	3	12
27	2	6
27	3	13
26	7	28
26	10	38
26	2	9
26	5	20
26	3	12
26	3	11
26	4	15

26	2	8
25	4	16
25	4	17
25	4	17
25	1	6
25	3	11
25	2	9
25	5	21
25	3	13
25	2	8
25	2	8
25	2	9
25	3	12
25	5	19
25	6	24
25	4	14
25	4	15
24	1	4
24	6	24
24	4	15
24	6	25
24	1	3
24	5	20
24	5	21
24	2	6
24	3	13
23	8	33
23	4	18
23	2	8
23	3	11
23	3	11
23	3	12
23	1	5
23	3	12
23	3	12
23	2	8
23	4	17
23	4	17
23	4	17
23	5	23
23	8	33
23	2	9
23	3	12
23	2	10
23	2	10
23	2	8
23	4	19
23	1	6

23	1	4
23	3	15
23	3	13
23	2	9
23	1	6
23	2	11
23	5	21
22	2	8
22	4	16
22	12	55
22	3	15
22	2	8
22	2	9
22	2	11
22	12	55
22	4	16
22	3	16
22	4	19
22	1	6
22	1	5
22	3	13
22	3	14
22	3	14
22	2	7
22	4	20
22	2	10
22	7	30
22	3	15
22	4	18
22	4	17
22	2	7
22	4	19
22	3	16
22	3	13
22	4	20
22	4	19
22	1	4
22	2	9
22	3	12
22	2	11
22	4	18
22	3	12
22	2	10
22	5	24
22	2	11
22	1	6
22	3	16
22	4	21

22	1	6
21	1	5
21	4	16
21	3	16
21	2	10
21	4	20
21	5	23
21	3	13
21	2	10
21	3	12
21	2	10
21	4	19
21	1	4
21	5	23
21	9	42
21	6	31
21	3	13
21	2	9
21	2	8
21	2	10
21	3	14
21	3	16
21	3	14
21	3	14
21	2	10
21	4	17
21	4	17
21	4	21
21	3	13
21	2	9
21	3	15
21	1	6
21	4	17
21	1	5
21	3	13
21	1	2
21	3	16
21	3	13
21	2	11
21	2	11
21	2	10
21	2	11
21	2	8
21	2	10
21	2	8
21	2	10
21	2	11
21	2	10

21	3	15
21	4	19
21	3	12
21	4	21
21	3	15
21	3	15
21	2	8
21	3	15
21	4	22
21	3	15
21	3	12
21	4	19
21	1	5
21	4	18
21	1	6
21	4	18
21	1	6
21	4	21
21	2	8
21	2	8
21	2	10
21	2	8
20	2	11
20	3	15
20	1	6
20	3	13
20	3	14
20	2	8
20	1	6
20	3	15
20	2	11
20	3	14
20	2	11
20	5	24
20	2	8
20	3	13
20	1	6
20	2	9
20	4	21
20	1	5
20	2	7
20	2	7
20	3	15
20	2	12
20	1	7
20	5	24
20	5	23
20	3	15

20	1	7
20	2	9
20	1	4
20	2	11
20	2	12
20	3	14
20	2	11
20	1	7
20	3	16
20	3	14
20	3	13
20	1	4
20	2	9
20	3	13
20	3	14
20	3	16
20	2	9
20	1	3
20	5	26
20	1	5
20	2	9
20	2	10
20	4	19
20	2	10
20	2	9
20	2	8
20	4	19
20	1	3
20	3	13
20	3	17
20	2	12
20	1	3
20	4	23
20	3	18
20	4	21
20	2	11
20	3	13
20	3	13
20	2	11
20	1	3
20	3	16
20	3	15
20	4	18
20	2	12
20	2	12
20	3	13
20	1	7
20	4	21

20	4	22
20	3	17
20	3	16
20	3	16
20	4	20
19	1	5
19	3	15
19	2	8
19	2	11
19	3	13
19	3	17
19	2	8
19	4	22
19	1	7
19	1	7
19	5	26
19	3	16
19	2	12
19	2	9
19	2	11
19	3	14
19	4	20
19	2	12
19	2	12
19	2	8
19	3	15
19	4	21
19	2	11
19	2	12
19	1	5
19	3	15
19	3	17
19	1	7
19	3	16
19	4	23
19	2	11
19	3	15
19	1	7
19	1	6
19	2	9
19	4	19
19	1	7
19	2	11
19	1	7
19	3	14
19	3	14
19	2	13
19	3	15

19	3	17
19	3	18
19	3	14
19	2	13
19	3	14
19	3	14
19	1	4
19	2	11
19	4	19
19	1	7
19	4	21
19	1	6
19	3	15
19	3	16
19	3	14
19	3	14
19	2	11
19	2	11
19	2	12
19	2	10
19	3	17
19	3	16
19	1	7
19	2	8
19	3	17
19	4	21
19	1	7
19	1	5
19	2	9
19	2	10
19	3	14
19	4	21
19	3	14
19	4	24
19	2	9
19	3	14
19	1	5
19	2	11
19	3	14
19	2	9
19	3	14
19	2	10
19	3	14
19	3	14
19	3	14
19	2	13
19	3	18
19	2	9

19	1	5
18	2	13
18	3	15
18	1	3
18	4	23
18	3	16
18	1	3
18	3	14
18	2	12
18	4	19
18	3	15
18	4	22
18	1	5
18	2	12
18	2	8
18	5	28
18	5	25
18	4	24
18	1	7
18	3	17
18	2	13
18	2	12
18	2	8
18	1	7
18	3	14
18	5	26
18	2	9
18	1	8
18	1	5
18	2	12
18	3	16
18	3	16
18	1	8
18	1	5
18	2	10
18	2	14
18	2	12
18	1	6
18	4	21
18	4	20
18	2	12
18	2	14
18	3	19
18	2	12
18	2	11
18	2	12
18	3	14
18	3	18

18	2	10
18	2	8
18	2	10
18	4	22
18	1	5
18	3	16
18	1	7
18	3	15
18	4	24
18	2	8
18	1	7
18	4	21
18	2	12
18	2	10
18	3	14
18	6	32
18	2	10
18	2	8
18	3	16
18	1	5
18	3	19
18	1	3
18	4	24
18	5	30
18	2	11
18	5	26
18	2	10
18	4	22
18	1	3
18	4	20
18	6	31
18	2	14
18	8	43
18	2	10
18	1	6
18	3	15
18	3	18
18	1	7
18	2	14
18	2	11
18	2	11
18	2	14
17	2	10
17	3	19
17	2	11
17	2	9
17	1	7
17	2	10

17	3	17
17	3	16
17	2	10
17	3	20
17	2	13
17	1	6
17	2	13
17	3	20
17	2	12
17	3	14
17	3	17
17	2	9
17	3	15
17	4	21
17	3	19
17	1	5
17	3	16
17	2	14
17	2	13
17	2	14
17	2	14
17	2	10
17	2	10
17	5	29
17	4	25
17	5	27
17	4	21
17	1	8
17	1	8
17	3	16
17	2	13
17	2	12
17	1	6
17	2	13
17	1	6
17	3	20
17	3	17
17	2	9
17	2	10
17	3	17
17	3	16
17	1	6
17	2	9
17	2	9
17	2	9
17	3	16
17	1	6
17	2	10

17	2	14
17	1	6
17	1	8
17	2	12
17	1	8
17	2	10
17	2	12
17	1	3
17	3	15
17	1	6
17	2	14
16	1	6
16	1	8
16	1	3
16	1	6
16	2	9
16	2	13
16	3	20
16	1	6
16	4	22
16	2	11
16	2	9
16	2	11
16	2	13
16	2	13
16	1	5
16	2	10
16	3	18
16	1	9
16	3	16
16	1	5
16	4	23
16	3	18
16	3	21
16	2	13
16	3	20
16	3	18
16	3	19
16	3	21
16	2	12
16	2	11
16	2	13
16	3	17
16	1	8
16	2	11
15	3	22
15	2	11
15	2	10

15	3	21
15	1	8
15	3	22
15	3	16
15	2	12
15	1	8
15	2	16
15	3	18
15	2	12
15	3	20
15	3	19
15	2	15
15	1	6
15	4	28
15	1	6
15	2	16
14	3	22
14	1	9
14	1	6
14	1	4
14	2	11
14	1	7
14	2	13
13	1	9
13	2	16
